# Supplementary material for: Molecular insights into the catalytic promiscuity of a bacterial diterpene synthase
Source: Nat Commun. 2023 Jul 6;14:4001. doi: 10.1038/s41467-023-39706-9 (PMC10325987; doi:10.1038/s41467-023-39706-9)
Supplement: Supplementary file 1 — Supplementary Information [file 41467_2023_39706_MOESM1_ESM.pdf]

Supplementary Information

Molecular insights into the catalytic promiscuity of a bacterial diterpene synthase

Zhong Li[1#], Lilan Zhang[2#], Kangwei Xu[3#], Yuanyuan Jiang[1], Jieke Du[1], Xingwang Zhang[1], Ling-Hong Meng[4,5], Qile Wu[1], Lei Du[1], Xiaoju Li[1], Yuechan Hu[2], Zhenzhen Xie[2], Xukai Jiang[1], Ya-Jie Tang[1], Ruibo Wu[3*], Rey-Ting Guo[2*], Shengying Li[1,5*]

[1]State Key Laboratory of Microbial Technology, Shandong University, No. 72 Binhai Road, Qingdao, Shandong 266237, China

[2]State Key Laboratory of Biocatalysis and Enzyme Engineering, Hubei Hongshan Laboratory, Hubei Collaborative Innovation Center for Green Transformation of Bio-Resources, Hubei Key Laboratory of Industrial Biotechnology, School of Life Sciences, Hubei University, Wuhan, 430062, China

[3]School of Pharmaceutical Sciences, Sun Yat-sen University, Guangzhou 510006, China.

[4]Key Laboratory of Experimental Marine Biology, Institute of Oceanology, Chinese Academy of Sciences, Nanhai Road 7, Qingdao, 266071, China

[5]Laboratory for Marine Biology and Biotechnology, Qingdao National Laboratory for Marine Science and Technology, Qingdao, Shandong 266237, China

[#]These authors contributed equally.

E-mail: lishengying@sdu.edu.cn (S. L.); guoreyting@hubu.edu.cn (R.-T. G.); wurb3@mail.sysu.edu.cn (R. W.)

Table of Contents

Supplementary Methods 3
Supplementary Tables 10
Supplementary Figures 25
Supplementary References 94

Supplementary Methods

Analytical methods. GC analysis was conducted using an Agilent HP-5 column (30 m × 0.32 mm × 0.25 μm) on an Agilent 7890B GC instrument with the following temperature program: 40 °C for 5 min, 40 to 300 °C at 10 °C/min, and 300 °C for 2 min. GC-MS analysis was performed using a Thermo DB-5MS Ultra Inert GC column (30 m × 0.25 mm × 0.25 μm) on Thermo Scientific Q Exactive™ GC Orbitrap™ high resolution GC-MS/MS with the temperature program as follow: 60 °C for 5 min, 60 to 300 °C at 10 °C/min, and 300 °C for 2 min. The low-resolution GC mass spectra of terpenoids were recorded on an Agilent Quadruple GC-MS instrument with the following program: 40 °C for 5 min, 40—300 °C at 10 °C/min, and 300 °C for 2 min. For the separation of **11** and **12** (Supplementary Fig. 47), the GC analysis was conducted using an Agilent HP-5 column (30 m × 0.32 mm × 0.25 μm) on an Agilent 7890B GC instrument with the following temperature program: 150 °C for 5 min, 150 to 200 °C at 2 °C/min, and 200 °C for 2 min. The GC, GC-MS and HPLC data collection and analysis were carried out using Agilent Openlab Control Panel, Thermo Scientific Xcalibur 4.2 SP1 and Thermo Chromeleon Console, respectively. The nuclear magnetic resonance (NMR) spectra were recorded on a Bruker Ascend™ 600 MHz spectrometer using Bruker TopSpin software. The X-ray single crystal diffraction was performed on a Rigaku XtaLAB Synergy Single Crystal X-ray Diffractometer using Rigaku CrysAlis Pro 40 and the data was output by CIF editor (publCIF) from the IUCr.

Bioinformatics analysis. Evolutionary analysis was conducted using MEGA-X software[1]. Protein sequence alignment was performed using ClustalW (http://www.clustal.org/) and the results were output by ESPript 3.0 (http://espript.ibcp.fr/ESPript/ESPript/). Sequence logos of conserved residues of TPSs were generated using the WebLogo online server (http://weblogo.threeplusone.com/). The sequence similarity network (SSN) was generated by an online service (https://efi.igb.illinois.edu/efi-est/) and output by Cytoscape. The structure of Bnd4 was predicted by the AlphaFold2[2] and the PP_i-$(Mg^{2+})_3$-bound form of VenA (PDB ID: 7Y9G) was used as the template to model the PP_i group and trinuclear magnesium cluster into Bnd4 (the protein sequence identity of VenA and Bnd4 is 30.0%).

Construction of protein expression vectors. To construct the plasmids for protein expression, the DNA fragment of *venA* was amplified from pET28b-*venA* using the primer pair of 28a-VenA-F/28a-VenA-R and the linearized pET-28a-*sumo* was amplified using the primers 28a-sumo-F/28a-sumo-R (Supplementary Table 15). The *venA* fragment was inserted into the linearized pET-28a-*sumo* to generate pET-28a-*sumo*-*venA*. To construct the plasmid for expressing the

N-terminal 15-residue truncated VenA (VenA$^{\Delta 1\text{-}15}$), the pET-28a-*sumo*-*venA* was amplified using the primers N-VenA-F/28a-VenA-R to remove the *N*-terminal 1-15 aa to generate pET-28a-*sumo*-*venA*$^{\Delta 1\text{-}15}$ by inserting into the linearized pET-28a-*sumo*.

For the site-directed mutagenesis of VenA, the linearized expression vectors for VenA mutants were PCR amplified from pET28b-*venA*^{WT} or pET28b-*venA*^{mutant} using the specific primer pairs as shown in Supplementary Table 15. Then, these linear fragments were self-ligated to generate recombinant vectors for the expression of mutated *venA* genes. To construct the VenA mutant and VenD co-expression vectors for terpenoids production in Eco-P (Supplementary Table 4), the DNA fragment of *venD*, including the coding sequence and the T7 promoter and terminator regions, was PCR amplified from pET28b-*venD* using the primer pair of T7VenD-F/T7VenD-R (Supplementary Table 15). Subsequently, this fragment was inserted into the linear mutant pET28b-*venA* to generate the co-expression vector (pET28b-*venA*^{mutant}*D*). All resulting plasmids were verified by DNA sequencing before use.

Protein expression and purification. A single transformant of BL21(DE3) containing pET28b-*venA*^{WT}, pET28b-*venA*^{mutant}, pET-28a-*sumo*-*venA*^{WT} or pET-28a-*sumo*-*venA*$^{\Delta 1\text{-}15}$ were grown overnight at 37 °C in LB (tryptone 10 g/L, yeast extract 5 g/L and NaCl 10 g/L) media containing 50 μg/mL kanamycin. Then, the seed culture was used to inoculate 0.5 L of TB medium (tryptone 12 g/L, yeast extract 24 g/L, K_2HPO_4 9.4 g/L, KH_2PO_4 2.2 g/L, and glycerol 40 g/L) containing 50 μg/mL of kanamycin at a ratio of 1:100 and shaking cultured at 37 °C, 220 rpm. When cell density reached an OD_{600} of 0.6, the culture temperature was lowered to 16 °C and subjected to induction with 0.2 mM isopropyl *β*-D-1-thiogalactopyranoside (IPTG) for 18 h. The cells were collected by centrifugation at 7,000 × *g* for 15 min at 4 °C and then suspended in 100 mL of lysis buffer containing 25 mM HEPES, 150 mM NaCl and 10 mM imidazole (pH = 7.5). The solution was passed through a French press (GuangZhou JuNeng Biology and Technology Co. Ltd, Guangzhou, China) to disrupt the cells, and the lysate was centrifuged at 35,000 × *g* for 1 h to remove cell debris. The supernatant solution was loaded onto a Ni-NTA column pre-equilibrated with 100 mL lysis buffer until the A_{280} reached baseline. The recombinant protein was eluted using a 10-500 mM imidazole gradient, and the target protein-containing fractions were identified by SDS-PAGE analysis and pooled. Finally, the purified proteins were flash frozen by liquid nitrogen and stored at −80 °C for later use.

The recombinant proteins Sumo-VenA^{WT} and Sumo-VenA$^{\Delta 1\text{-}15}$ were then reacted with SUMO protease overnight at 4 °C when dialyzed against a buffer containing 25 mM HEPES, 150 mM NaCl (pH = 7.5) to remove imidazole. The resulting solution was loaded on a Ni-NTA column again and the flow-through fractions were collected. The protein concentration was

determined by NanoDrop (Thermo Scientific). Prior to crystallization trials, protein solutions were stored at -80 °C.

Structure determination of 4, 5, 7, 13, 14, 15, 16, 17 and 18*. The identities of *β*-farnesene (**4**), germacrene D (**5**), geranyllinalool (**13**), geranylgeraniol (**14**), *α*-farnesene (**15**), nerolidol (**16**), and farnesol (**17**) were confirmed by comparing the retention time and mass spectra with those of the corresponding authentic standards (Supplementary Figs. 91−97). The structures of *β*-springene (**7**, retention index = 1917) and shyobunol (**18***, retention index = 1525) were determined based on the comparison of the experimental mass spectra and retention indexes with those of the authentic standards in the NIST library and literature (Supplementary Figs. 27 and 98)[3, 4] . The injected germacradien-6-ol (**18**) was converted into **18*** by Cope rearrangement during GC-MS analysis due to the flexible *trans*, *trans*-cyclodeca-1(10),4(5)-diene ring system according to the previous report (Supplementary Figs. 4-5)[5]. The numbering of carbon atoms in **18** was performed according to the previous literature[5].

Isolation, purification, and characterization of 8, 9, 10, 11, 12 and 19. A single colony of Eco-A^{Y88A}D, Eco-A^{W107A}D, Eco-A^{V111W}D or Eco-A^{F185A}D (Supplementary Table 4) was picked and grown in 100 mL LB medium containing 50 μg/mL of kanamycin, 25 μg/mL of chloramphenicol, and 100 μg/mL of ampicillin at 37 °C, 220 rpm for 12 h. Then, the seed culture was used to inoculate 10-50 L TB medium containing 50 μg/mL of kanamycin, 25 μg/mL of chloramphenicol, and 100 μg/mL of ampicillin at a ratio of 1:100 at 37 °C, 220 rpm. When OD_{600} reached 1.0-1.5, the protein overexpression for terpene/terpenoid production was initiated by adding IPTG to the final concentration of 0.2 mM. After additional cultivation at 18 °C for 96 h, 500 μL fermentation broth of engineered *E. coli* was extracted by an equal volume of ethyl acetate, vortexed for 10 min, and centrifuged at 14,000 × *g* for 10 min. Then, the supernatant was directly used for GC/GC-MS analysis to monitor the terpene/terpenoid production. The yield of **9** was calculated by comparing the peak areas of two independent fermentation samples with that of an authentic standard with known concentration during GC analysis. Finally, the fermentation broth of engineered *E. coli* was extracted with an equal volume of ethyl acetate three times and the organic extracts were combined and concentrated by vacuum rotary evaporation. The crude extracts were further extracted using 50 mL *n*-hexane four times, which were concentrated *in vacuo* and re-dissolved in acetonitrile. For the isolation of **8**, the concentrated extract of Eco-A^{Y88A}D was purified by semi-preparative HPLC on a YMC C-18 column (10 × 250 mm, 5 μm) with 100% acetonitrile over 30 min at a flow rate of 3 mL/min. For the isolation of **9** and **10**, the concentrated extracts of Eco-A^{W107A}D and Eco-A^{V111W}D were

respectively purified by semi-preparative HPLC on a YMC C-18 column (10 × 250 mm, 5 μm) with 100% methanol over 30 min at a flow rate of 3 mL/min. For the isolation of **11** and **12**, the concentrated extract of Eco-A^{F185A}D was purified by semi-preparative HPLC according to their retention time at 32−33 min (**11**) and 31−32 min (**12**) on a YMC C-18 column (10 × 250 mm, 5 μm) with 100% acetonitrile using the procedure as follow: 0−17 min, the flow rate was 1.5 mL/min; 17.5−23 min, the flow rate was 2.0 mL/min; 23.5−29 min, the flow rate was 2.5 mL/min; and 29.5−35 min, the flow rate was 3 mL/min.

For *in vitro* preparation of **19**, a 600 mL reaction mixture containing 10 μM $VenA^{Y88A/W107A/V111A/T112A/F215A/F219A}$, 10 mM $MgCl_2$, and 100 μM GFPP in Tris-HCl buffer (50 mM, 10% glycerol, pH = 7.4) was incubated at 30 °C for 2 h. The enzyme reaction mixture was extracted using 600 mL *n*-hexane for three times, which were combined and concentrated *in vacuo* and re-dissolved in acetonitrile. For the isolation of **19**, the concentrated extract was purified by semi-preparative HPLC on a YMC C-18 column (10 × 250 mm, 5 μm) with 100% methanol over 35 min at a flow rate of 3 mL/min.

ECD analysis. The ECD spectra were recorded on a JASCO J-1500 spectropolarimeter (JASCO Corporation, Tokyo, Japan) using the SpectraManager software. ECD spectra were generated using the program SpecDis21 by applying a Gaussian band shape with 0.3 eV width for **8** and 0.22 eV width for **10** from dipole length rotational strengths (Supplementary Figs. 35 and 73). The spectra of the conformers were combined using Boltzmann weighting with the lowest-energy conformations accounting for about 99% of the weights. The calculated spectra were shifted by 3 nm for **8**, and 13 nm for **10** to facilitate comparisons to the experimental data.

Structure elucidation of 8, 9, 10, 11, 12 and 19. The purified **8** was colorless oil with $[\alpha]_D^{20}$ = +7.7 (*c* 0.117, CH_2Cl_2), UV_{max} = 210 nm; and its structure was determined to be a known diterpene (*S*)-(+)-cembrene A by ECD analysis, and comparing the NMR and optical rotation data with the published data from the previous literature[6] (Supplementary Table 5, Supplementary Figs. 28-35).

The purified **9** was colorless oil with $[\alpha]_D^{20}$ = −27.5 (*c* 0.417, CH_2Cl_2), UV_{max} = 210 nm, retention index = 1956; and its structure was determined to be a new diterpene (named as (1*S*,3*E*,7*E*,11*R*,12*S*)-3,7,18-dolabellatriene) with a 5-11 skeleton based on GC-MS, NMR and X-ray single crystal analyses (Supplementary Table 6, Supplementary Figs. 36-44). The planar structure of **9** was determined from ^1H-^1H and ^1H-^{13}C correlations. Briefly, the five-membered ring was deduced from the ^1H-^1H correlations of H11–H12–H13–H14 and the ^1H-^{13}C

correlations between C1 and H11, H14. The eleven-membered ring was built based on the ^{1}H-^{1}H correlations of H2–H3, H5–H6–H7 and H9-H10-H11, and the ^{1}H-^{13}C correlations from H15 to C1, C2, C11, from H17 to C7, C8 and C9, and from H16 to C3, C4 and C5. The absolute configuration of **9** was assigned as 1*S**, 11*R**, 12*S** based on the X-ray single crystal analysis (Supplementary Fig. 37).

The purified **10** was colorless oil with $[\alpha]_D^{20}$ = −67.0 (*c* 0.125, CH_2Cl_2), UV_{max} = 210 nm, retention index = 1905; and its structure was identified as a new prenylated-guaiane diterpene based on GC-MS, NMR and electronic circular dichroism (ECD) analyses, and named as dictytriene C, following dictytrienes A and B with the same 5-7 skeleton isolated from the alga *Dictyota dichotoma*[7] (Supplementary Table 9, Supplementary Figs. 65-73 and 99). The planar structure of **10** was determined from ^{1}H-^{1}H and ^{1}H-^{13}C correlations as follows: the five-membered ring was deduced from ^{1}H-^{1}H correlations of H2−H3−H4−H5−H6; the seven-membered ring was deduced from ^{1}H-^{1}H correlations of H1−H2−H6 and H8−H9, as well as the ^{1}H-^{13}C correlations from H19 to C6, C7 and C8, H9 to C10, and H1 to C10. Finally, the absolute configuration of **10** was assigned as 2*R**, 3*R**, 6*S**, 11*S** based on the ECD analysis (Supplementary Fig. 73).

The purified **11** was colorless oil with $[\alpha]_D^{20}$ = −51.1 (*c* 0.083, CH_3CN), retention index = 2254; and its structure was determined to be a new diterpenoid (named as venezuelaenol A) with a 5-5-6-7 skeleton based on GC-MS, NMR and X-ray single crystal analyses (Supplementary Table 7, Supplementary Figs. 48-55). The planar structure of **11** was determined from ^{1}H-^{1}H and ^{1}H-^{13}C correlations as follows: the seven-membered ring was deduced from the ^{1}H-^{1}H correlations of H8−H9−H10−H1−H2 and the ^{1}H-^{13}C correlations between H6, H8 and C7. The six-membered ring was built based on the ^{1}H-^{1}H correlations of H1−H10−H11−H12−H13−H14. The ^{1}H-^{1}H correlation signals of H2−H1−H14 and H4−H5−H6−H2 as well as the ^{1}H-^{13}C correlations from H16 to C3, C14 and C15, from H2 to C1, C3, C6 and C20, and from H20 to C3, C4 and C15 revealed the two merged five-membered rings. Finally, the absolute configuration of **11** was assigned as 1*R**, 2*R**, 3*R**, 6*R**, 7*R**, 10*S**, 11*R**, 14*S** based on its crystal structure (Supplementary Fig. 49).

The purified **12** was colorless oil with $[\alpha]_D^{20}$ = −24.6 (*c* 0.133, CH_3CN), retention index = 2255; and its structure was determined to be a new diterpenoid (named as venezuelaenol B) with a novel 6-5-6-6 skeleton based on GC-MS and NMR analyses (Supplementary Table 8, Supplementary Figs. 56-63). The planar structure of **12** was determined from ^{1}H-^{1}H and ^{1}H-^{13}C correlations as follows: the five-membered ring was deduced from the ^{1}H-^{1}H correlations of H1−H2−H14, as well as the ^{1}H-^{13}C correlations from H14 to C15, H20 to C3 and C15, and H16

and H17 to C3, C14 and C15; the ^1H-^1H correlations of H1−H10−H11−H12−H13−H14, H2−H1−H10−H9−H8, H4−H5−H6 and ^1H-^{13}C correlations between H2, H6, H8 and C7 revealed the three merged six-membered rings. Similar with the previous report[8], the absolute configuration of **12** was assigned as 1*R**, 2*S**, 3*R**, 6*R**, 7*R**, 10*S**, 11*R**, 14*S** based on the NOESY spectrum and the absolute configuration of intermediate **F** (Fig. 5), which is the biosynthetic intermediate of **1**, **11** and **12**.

The purified **19** was colorless oil with $[\alpha]_D^{20}$ = −20.1 (*c* 0.108, CH_3CN), UV_{max} = 206 nm; and its structure was determined to be a known sesterterpene (*S*)-cericerene by comparing the NMR data and CD spectrum with the published data from the previous literature[9] (Supplementary Table 11, Supplementary Figs. 79-87).

Determination of relative products ratios and catalytic efficiencies of VenA wild-type and mutants. The concentrations of **1−6** and **8−17** in the enzymatic assays of VenA wild-type and mutants were determined by comparing each compound with its authentic standard during GC-MS analysis. Similar to the previous reports[10], the percentage concentrations of **7**, **18** and unassigned products were calculated from their peak areas relative to the peak areas of the homologues with known concentrations during GC−MS analysis. The conversion ratio of an enzymatic assay was calculated by comparing each product with its authentic standard or homologues during GC analysis. The conversion ratio of VenA wild type toward GGPP was assigned as 100%. The triplicated data (mean ± SD) were used to compare the relative catalytic efficiencies between VenA wild type and mutants toward GGPP, FPP and GPP.

X-ray single crystal analysis of 9 and 11. About 8 mg of purified (1*S*,3*E*,7*E*,11*R*,12*S*)-3,7,18-dolabellatriene (**9**) was dissolved in 0.5 mL acetonitrile and the crystal was obtained under 4 °C. Selected key X-ray data: crystal size, 0.12 × 0.10 × 0.06 mm; $C_{20}H_{32}$; Mr = 272.45; orthorhombic, space group $P2_12_12$, *a* =6.5547 (1) Å, *b* = 12.5564 (1)Å, *c* = 20.8095 (2) Å, *V* = 1712.69 (3) $Å^3$, *D*calc. = 1.057 g/cm^3, *Z* = 4, *T* = 293 K, *F* (000) = 608, μ = 0.425 mm^{-1}, Cu Kα radiation, λ = 1.54184 Å. 16065 reflections measured (8.224° ≤ 2θ ≤ 153.028°), 3464 independent (Rint = 0.0486, Rsigma = 0.0311) which were used in all calculations. Final R indexes ($I > 2\sigma(I)$): R_1 = 0.0388, wR_2 = 0.0958; final R indexes [all data]: R_1 = 0.0421, wR_2 = 0.0987. Crystallographic data (including structure factors) for **9** (CCDC 2163519) were deposited in the Cambridge Crystallographic Data Center.

About 2 mg of purified venezuelaenol A (**11**) was dissolved in 0.2 mL acetonitrile and the crystal was obtained under 4 °C. Selected key X-ray data: crystal size, 0.11 × 0.1 × 0.08 mm; $C_{20}H_{34}O$; Mr = 290.47; monoclinic, space group $P2_1$, *a* = 16.33139(12)Å, *b* = 10.48287(7) Å, *c*

= 21.11296(15) Å, V = 3546.85(4) Å^3, Dcalc. = 1.088 g/cm^3, Z = 8, T = 100 K, F (000) = 1296.0, μ= 0.479 mm^{-1}, Cu Kα radiation, λ = 1.54184 Å. 61634 reflections measured (4.266° ≤ 2θ ≤ 132.002°), 12327 independent (Rint = 0.0300, Rsigma = 0.0208) which were used in all calculations. Final R indexes (I > 2σ(I)): R_1 = 0.0565, wR_2 = 0.1453; final R indexes [all data]: R_1 = 0.0810, wR_2 = 0.1579. Crystallographic data (including structure factors) for **11** (CCDC 2184782) were deposited in the Cambridge Crystallographic Data Center.

Antimicrobial assays. The antimicrobial activities of **1**, **9**-**12**, and the positive control (kanamycin) against seven Gram-positive bacteria (Ampicillin-resistant *Bacillus subtilis*, *B. subtilis* RIK1285, *B. subtilis* 168, *Bacillus cereus*, *Clavibacter michiganense* subsp. *sepedonicus*, *Staphylococcus aureus* ATCC 25923, and *Mycolicibacterium smegmatis* MC155) and four Gram-negative plant-pathogenic bacteria (*Pectobacterium carotovorum* subsp. carotovorum, *Pseudomonas syringae* pv. *lachrymans*, *Xanthomonas campestris* pv. *campestris*, and *Xanthomonas oryzae* pv. *oryzicola* RS105) were tested by the broth microdilution method[11]. Briefly, the tested strains were cultivated in LB broth at 30 °C. Compounds **1**, **9**-**12**, and kanamycin were individually dissolved in DMSO and diluted from 256 μg/mL to 0.125 μg/mL in 96-well plates using the serial 2-fold dilution method. The minimum inhibitory concentrations (MICs, the lowest concentration of the tested compounds when no microbial growth can be detected) were recorded (Supplementary Table 14).

Binding energy calculation. To calculate the relative binding energies, we pairwise extracted the structures of several important residues and intermediates from the QM/MM scan trajectories. The residues were truncated at the *α*-carbon and saturated with hydrogen atoms. The relative binding energies were computed using Gaussian 16 with the same method and basis set used in the QM region of the QM/MM calculation. To avoid basis set superposition errors, we employed counterpoise correction method developed by Boys and Bernardi[12].

Supplementary Tables

Supplementary Table 1. X-ray diffraction data and structural refinement statistics of VenA crystal structures.

	VenA-nat	VenA-PP_i
**Data collection**		
Space group	$P3_2$	$P3_2$
**Unit cell**		
a/*b*/*c* [Å]	94.0/94.0/80.8	92.8/92.8/81.1
α /*β* /*γ* (°)	90/90/120	90/90/120
Resolution [Å]	36.35-2.03 (2.06-2.03)	36.00-2.18 (2.21-2.18)
Unique reflections	51568 (2194)	40768 (1690)
Redundancy	4.3 (3.1)	4.5 (3.4)
Completeness [%]	99.9 (99.4)	99.9 (100)
Average I/σ (I)	13.2 (2.2)	10.4 (2.1)
R_{merge} [%][a]	6.3 (37.6)	6.5 (41.4)
CC1/2	0.998	0.998
**Refinement**		
No. of reflections	48888 (3578)	36822 (2375)
R_{work} [%]	16.0 (23.2)	17.5 (25.6)
R_{free} [%]	20.6 (24.1)	23.8 (30.1)
r.m.s.d. bonds [Å]	0.010	0.008
r.m.s.d. angles [º]	1.54	1.46
Dihedral angles		
Most favored [%]	99.6	99.8
Allowed [%]	0.4	0.2
Disallowed [%]	0	0
**No. of non-H atoms / Average B [Å^2]**		
Protein	5282/31.7	5161/42.6
Water	635/38.0	462/44.3
Ligand	45/57.6	118/58.7
PDB ID code	7Y9H	7Y9G

Values in parentheses are for the highest resolution shell.

[a] $R_{merge} = \sum_{hkl}\sum_i |I_i(hkl) - \langle I(hkl)\rangle| / \sum_{hkl}\sum_i I_i(hkl)$.

Supplementary Table 2. The relative percentages of di-, sesqui- and monoterpenoid products of VenA wild type and mutants. u.p. stands for the unidentified products. n.d. stands for no products detected under the conditions of the enzymatic assays used in this study.

VenA	Di-	Sesqui-	Mono-
**WT**	**1**: 92.1%; u.p.: 7.9%	**2**: 43.8%; **3**: 13.0%; **4**: 9.9%; **5**: 7.9%; u.p.: 25.4%	**6**: 100%
**Y88A**	**8**: 68.1%; **7**: 18.6%; u.p.: 13.3%	**4**: 53.1%; **15**: 16.1%; **17**: 14.7%; **16**: 3.7%; **3**: 2.7%; u.p.: 9.7%	**6:** 100%
**Y88W**	n.d.	n.d.	**6:** 100%
**W107A**	**9**: 52.1%; **1**:41.1%; u.p.: 6.8%	**2**: 27.7%; **4**: 20.8%; **3**: 13.2%; **17**: 10.0% **16**: 3.4%; u.p.: 24.9%	**6**: 100%
**V111W**	**1**: 37.2%; **10:** 26.5%; **9**: 7.0%; u.p.: 29.3%	**2**: 62.2% **3**: 8.0% **4**: 4.3% **5**: 2.8%; u.p.: 22.7%	**6**: 100%
**T112W**	n.d.	**18**: 46.1%; **5**: 43.3%; u.p.: 10.6%	n.d.
**F185A**	**12**: 40.7%; **1**: 25.8 %; **11**: 21.4%; u.p.: 12.1%	n.d.	n.d.
**F215A**	**10:** 12.5%; **9**: 9.0%; **1**: 5.3%; u.p.: 73.2%	**4**: 39.2%; **17**: 24.4%; **16**: 14.7%; **2**: 8.8%; **3**: 6.8%; u.p.: 6.1%	**6**: 100%
**F219A**	**1**: 66.0%; **9**: 27.3%; u.p.: 6.7%	**17**: 47.6%; **2**: 13.5%; **4**: 11.6%; **3**: 7.0%; **16**: 10.2%; u.p.: 10.1%	**6**: 100%
**R344K**	**1**: 100%	**2**: 56.8%; **18**: 25.5%; **3**: 4.5%; **4**: 3.4%; u.p.: 9.8%	n.d.
**Y345F**	**1**: 11.5%; u.p.: 88.5%	**4**: 42.0%; **2**: 28.6%; **3**: 26.8%; u.p.: 2.6%	n.d.
**W107A /F219A**	**1:** 37.0%**;** **9:** 11.4%; **12:** 11.2%; **13**: 19.1%; u.p.: 21.3%	**17**: 70.1%; **16**: 18.2%; **4**: 9.3%; **2**: 2.4%	**6**: 100%

Supplementary Table 3. The calculated relative binding energies.

Intermediates	Amino acids (relative binding energy in kcal/mol)
**A**	Phe215 (-7.0)
**B**	Tyr88 (-9.7); Phe215 (-6.6)
**C**	Tyr88 (-9.6); Phe215 (-6.8)
**D**	Phe215 (-5.7); Tyr88 (-2.7)
**E**	F215 (-7.3)
**F**	Phe215 (-10.9); F185 (-0.7)
**G**	Phe215 (-10.3); F185 (-1.4)

Supplementary Table 4. The strains used in this study. Abbreviations: Kan, kanamycin; Amp, ampicillin; and Chl, chloramphenicol.

Strains	Characteristics	Resistance	References
Escherichia coli			
DH5a	Cloning host	-	[13]
BL21 (DE3)	Protein expression host	-	[14]
Eco-P	BL21(DE3)/pACYC-*mavEmavS* & pTrc-*low* for improved production of IPP and DMAPP	Amp, Chl	[15]
Eco-A^{Y88A}D	Eco-P/pET28b-*venA*^{Y88A}*D*	Kan, Amp, Chl	This study
Eco-A^{W107A}D	Eco-P/pET28b-*venA*^{W107A}*D*	Kan, Amp, Chl	This study
Eco-A^{F185A}D	Eco-P/pET28b-*venA*^{F185A}*D*	Kan, Amp, Chl	This study
Eco-A^{F215A}D	Eco-P/pET28b-*venA*^{F215A}*D*	Kan, Amp, Chl	This study
Eco-A^{V111W}D	Eco-P/pET28b-*venA*^{V111W}*D*	Kan, Amp, Chl	This study

Supplementary Table 5. NMR data of (*S*)-(+)-cembrene A (**8**).

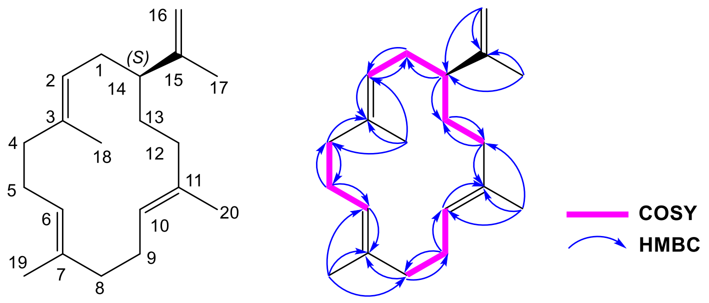

Key ^{1}H-^{1}H COSY, and HMBC correlations of **8**.

^{1}H NMR (600 MHz, $CDCl_3$) and ^{13}C NMR (151 MHz, $CDCl_3$) data

Position	δ_C, type	δ_H, mult., (*J* in Hz)
1	32.4, CH_2	1.95, m; 2.00, m
2	124.1, CH	5.19, m
3	134.8, C	
4	39.0, CH_2	2.17, m; 2.12, m
5	24.9, CH_2	2.26, m; 2.17, m
6	125.9, CH	4.98, m
7	133.5, C	
8	39.4, CH_2	2.06, m
9	23.8, CH_2	2.12, m
10	121.9, CH	5.06, m
11	133.9, C	
12	34.0, CH_2	1.94, m; 1.78, m
13	28.2, CH_2	1.67, m; 1.38, m
14	46.0, CH	2.03, m
15	149.3, C	
16	110.1, CH_2	4.71, m; 4.65, m
17	19.3, CH_3	1.66, s
18	15.5, CH_3	1.57, s
19	15.3, CH_3	1.59, s
20	18.0, CH_3	1.56, s

Supplementary Table 6. NMR data of (1*R*,3*E*,7*E*,11*S*,12*S*)-3,7,18-dolabellatriene (**9**). The carbon atoms are numbered according to the previous literature[16].

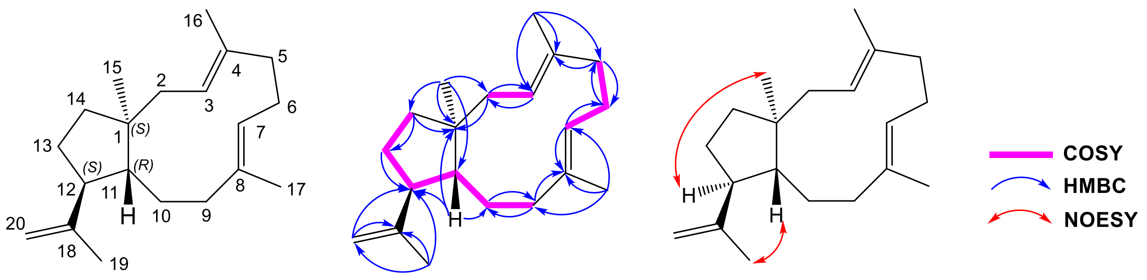

Key ^{1}H-^{1}H COSY, HMBC and NOESY correlations of **9**.

^{1}H NMR (600 MHz, $CDCl_3$) and ^{13}C NMR (151 MHz, $CDCl_3$) data.

Position	δ_C, type	δ_H, mult., (*J* in Hz)
1	44.9, C	
2	40.5, CH_2	2.12, m; 1.80, m
3	124.3, CH	4.98, m
4	133.9, C	
5	39.6, CH_2	2.09, m
6	24.8, CH_2	2.14 m, 2.11 m
7	125.0, CH	4.86, t, (7.5)
8	136.0, C	
9	37.3, CH_2	2.00, m; 1.81, m
10	30.7, CH_2	1.44, m; 1.26, m
11	44.4, CH	1.49, m
12	57.9, CH	2.24, m
13	28.9, CH_2	1.60, m; 1.50, m
14	41.5, CH_2	1.37 m, 1.59 m
15	23.2, CH_3	0.92, s
16	16.0, CH_3	1.52, s
17	17.4, CH_3	1.51, s
18	148.6, C	
19	19.0, CH_3	1.73, s
20	110.9, CH_2	4.78, d (2.5); 4.66, dd (2.5, 1.4)

Supplementary Table 7. ^{1}H NMR (600 MHz, $CDCl_3$) and ^{13}C NMR (151 MHz, $CDCl_3$) data of venezuelaenol A (**11**).

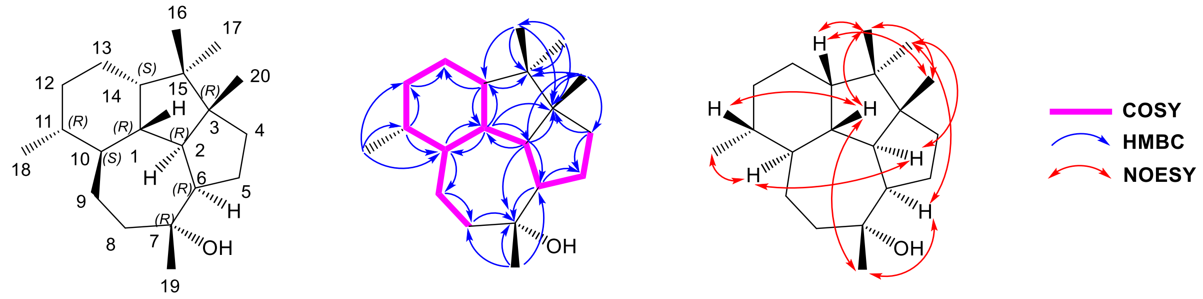

Key ^{1}H-^{1}H COSY, HMBC and NOESY correlations of **11**.

^{1}H NMR (600 MHz, CD_3CN) and ^{13}C NMR (151 MHz, CD_3CN) data.

Position	δ_C, type	δ_H, mult., (J in Hz)
1	44.1, CH	1.55, m
2	55.6, CH	2.30, m
3	55.8, C	
4	32.4, CH_2	1.35, m; 1.16, m
5	32.8, CH_2	2.07, m; 1.98, m
6	47.1, CH	2.07, m
7	75.0, C	
8	49.1, CH_2	1.78 m, 1.51, m
9	32.1, CH_2	1.81, m; 0.96, m
10	44.7, CH	0.83, m
11	32.5, CH	1.16, m
12	33.0, CH_2	1.64, m; 1.19, m
13	20.8, CH_2	1.26, m
14	53.2, CH	1.87, m
15	41.4, C	
16	26.9, CH_3	0.91, s
17	22.1, CH_3	0.84, s
18	20.8, CH_3	0.85, d, (6.5)
19	23.7, CH_3	1.18, s
20	19.6, CH_3	0.90, s

Supplementary Table 8. ^{1}H NMR (600 MHz, $CDCl_3$) and ^{13}C NMR (151 MHz, $CDCl_3$) data of venezuelaenol B (**12**).

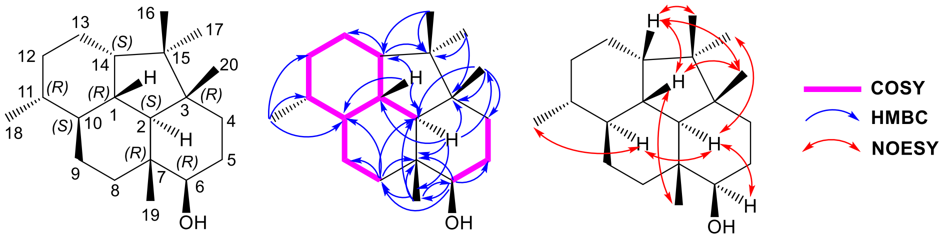

Key ^{1}H-^{1}H COSY, HMBC and NOESY correlations of **12**.

^{1}H NMR (600 MHz, CD_3CN) and ^{13}C NMR (151 MHz, CD_3CN) data.

**Position**	**δ_C, type**	**δ_H, mult., (J in Hz)**
1	39.5, CH	1.40, m
2	56.5, CH	1.22, m
3	45.5, C	
4	31.8, CH_2	1.38, m; 1.23, m
5	29.8, CH_2	1.66, m; 1.51, m
6	82.0, CH	3.15, m
7	40.6, C	
8	44.1, CH_2	1.76, m; 1.05, m
9	28.9, CH_2	1.69, m; 0.90, m
10	46.6, CH	0.80, m
11	32.3, CH	1.26, m
12	32.4, CH_2	1.62, m; 1.30, m
13	19.6, CH_2	1.31, m; 1.19, m
14	48.5, CH	1.76, m
15	46.7, C	
16	25.3, CH_3	0.86, s
17	22.6, CH_3	0.78, s
18	21.1, CH_3	0.89, d, (6.8)
19	14.5, CH_3	0.91, s
20	17.0, CH_3	0.86, s

Supplementary Table 9. NMR data of dictytriene C (**10**).

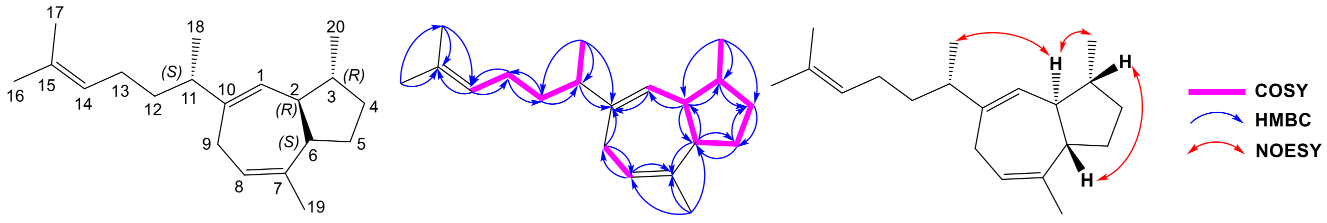

Key ^{1}H-^{1}H COSY, HMBC and NOESY correlations of **10**.

^{1}H NMR (600 MHz, $CDCl_3$) and ^{13}C NMR (151 MHz, $CDCl_3$) data.

**Position**	**δ_C, type**	**δ_H, mult., (*J* in Hz)**
1	120.4, CH	5.39, m
2	43.5, CH_2	2.88, m
3	38.5, CH	1.99, m
4	29.3, CH_2	1.72, m; 1.25, m
5	28.8, CH_2	1.88, m; 1.35, m
6	48.7, CH	2.27, m
7	138.7, C	
8	120.4, CH	5.16, m
9	27.2, CH_2	2.92, m; 2.14, m
10	150.3, C	
11	41.7, CH	2.09, m
12	34.6, CH_2	1.35, m; 1.23, m
13	25.9, CH_2	1.88, m; 1.83, m
14	125.0, CH	5.08, m
15	131.0, C	
16	25.7, CH_3	1.67, s
17	17.7, CH_3	1.56, s
18	19.4, CH_3	0.95, s
19	15.8, CH_3	1.04, s
20	24.5, CH_3	1.55, s

Supplementary Table 10. The calculated volumes of hydrophobic pockets for accepting the oligoprenyl side chains of substrates based on closed forms of the type I di-, sesqui- and monoterpene synthases. *Note*: PaFS* is the cyclase domain of PaFS.

TPSs	PDB ID code	Volume (Å^3)	Reference
**Diterpene synthase**			
Taxadiene synthase	3P5R	630.3	[17]
PaFS*	5ER8	467.5	[18]
CotB2	6GGI	489.5	[19]
VenA	7Y9G	516.1	This study
**Average Value**		**525.9**	
**Sesquiterpene synthase**			
Pentalenene synthase	6WKD	443.4	[20]
EIZS	7KJ9	427.5	[21]
Selinadiene synthase	4OKZ	499.7	[10]
5-*epi*-aristolochene synthase	5EAT	437.8	[22]
SaSQS1	6O9P	389.6	[23]
Trichodiene synthase	2Q9Y	430.1	[24]
**Average Value**		**438.0**	
**Monoterpene synthase**			
(*S*)-(−)-limonene synthase	2ONG	357.9	[25]
(*R*)-(+)-limonene synthase	5UV1	384.5	[26]
1,8-cineole synthase	5NX7	372.7	[27]
**Average Value**		**371.7**	

Supplementary Table 11. NMR data of **19**.

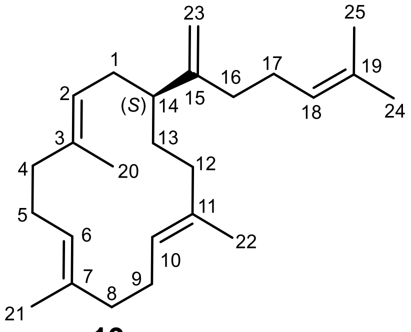

19

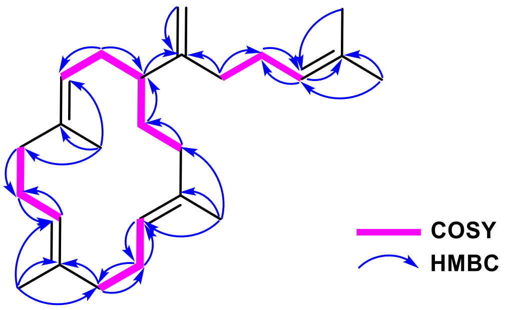

Key ^{1}H -^{1}H COSY, and HMBC correlations of **19**.

^{1}H NMR (600 MHz, CD_3CN) and ^{13}C NMR (151 MHz, CD_3CN) data

Position	δ_C, type	δ_H, mult., (*J* in Hz)	Position	δ_C, type	δ_H, mult., (*J* in Hz)
1	33.7, CH_2	1.91, m; 2.02, m	14	45.3, CH	2.03, m
2	125.2, CH	5.21, m	15	154.7, C	
3	135.6, C		16	35.6, CH_2	1.99, m
4	39.6, CH_2	2.15, m; 2.12, m	17	27.6, CH_2	2.09, m
5	25.6, CH_2	2.26, m; 2.16, m	18	125.3, CH	5.14, m
6	126.8, CH	4.97, m	19	132.3, C	
7	134.2, C		20	15.6, CH_3	1.56, s
8	40.2, CH_2	2.03, m	21	15.4, CH_3	1.58, s
9	24.4, CH_2	2.12, m	22	18.0, CH_3	1.54, s
10	122.9, CH	5.08, m	23	108.7, CH_2	4.74, s; 4.78, s
11	134.6, C		24	25.8, CH_3	1.67, s
12	35.0, CH_2	1.94, m; 1.78, m	25	17.8, CH_3	1.60, s
13	29.7, CH_2	1.70, m; 1.45, m			

Supplementary Table 12. The type I DTSs using GGPP as the substrate, the crystal structures of which have been elucidated. *Note*: TXS stands for taxadiene synthase; PaFS* is the cyclase domain of PaFS; SvS-A2 is a reconstructed ancestor of SvS.

**DTS**	**TXS**	**ErTC-2**	**PaFS***	**CotB2**	**SvS-A2**	**CyS**
**PDB ID code**	3P5R	7S5L	5ER8	6GGI	6TBD	7Y88
**Origin**	*Taxus brevifolia*	*Eleutherobia rubra*	*Phomopsis amygdali*	*Streptomyces melanosporofaciens*	*Streptomyces sp.* CWA1	*Streptomyces cattleya*
**Initial cyclization step for GGPP**	1,14-cy	1,14-cy	1,11-cy	1,11-cy	1,11-cy	1,11-cy
**The ring system of the diterpene**	6-8-6	14	5-8-5	5-8-5	5-5-5-5	5-6-5-5
**Modular architecture**	α-β-γ	α	α	α	α	α
**Reference**	[17]	[28]	[18]	[19, 29]	[30]	[8]

Supplementary Table 13. The predication of the amino acid residues in the active pocket of the select number of identified DTSs using VenA as a reference. The DTSs mediated the initial 1,10/1,11-cyclization of GGPP are marked in red.

	88	107	108	185	215	219	338	Initial cyclization mode of GGPP
VenA	**Y**	W	Y	F	F	F	F	1,10-cy
Bnd4	**W**	Y	T	F	V	M	W	1,10-cy
AlbS	**F**	W	H	Y	A	V	W	1,10-cy
SvS-A2	**F**	W	V	W	A	P	W	1,11-cy
SvS	**F**	W	V	W	A	P	W	1,11-cy
PmS	**F**	W	G	W	A	P	W	1,11-cy
CyS	**Y**	W	C	W	A	A	W	1,11-cy
DtcycA	V	F	L	W	V	L	A	1,14-cy
CAS	V	F	Y	F	S	A	W	1,14-cy
Rxyl_0493	L	W	C	E	G	V	W	1,14-cy
CwWS	I	Y	T	V	S	Y	W	1,14-cy
CpPS	I	Y	A	V	S	Y	W	1,14-cy
McS	A	L	F	H	S	V	W	1,15-cy

Supplementary Table 14. The antibacterial activities of 1 and 9-12.

**Strains**	**MIC (µg/mL)**					
	**1**	**9**	**10**	**11**	**12**	Kanamycin
Ampicillin-resistant *Bacillus subtilis*	>256	>256	32	>256	>256	4
Bacillus subtilis 168	>256	>256	16	32	>256	2
Bacillus subtilis RIK1285	>256	>256	16	256	>256	1
Bacillus cereus	>256	>256	8	64	>256	8
Clavibacter michiganense subsp. *sepedonicus*	>256	>256	32	256	>256	4
Mycolicibacterium smegmatis MC155	>256	>256	32	>256	>256	4
Staphylococcus aureus ATCC 25923	>256	>256	8	4	>256	2
Pectobacterium carotovorum subsp. *carotovorum**	>256	>256	>256	>256	>256	8
Pseudomonas syringae pv. *lachrymans**	>256	>256	16	4	>256	1
Xanthomonas campestris pv. *campestris**	>256	>256	256	256	>256	4
Xanthomonas oryzae pv. *oryzicola* RS105*	>256	>256	32	256	>256	32

Note: "*" stands for the Gram-negative plant-pathogenic bacteria

Supplementary Table 15. The primers used in this study.

Primers	Sequence (5’-3’)
**28a-VenA-F**	CAGATTGGTGGATCCATGCAGCAACGCCTCCGCC
**28a-VenA-R**	GCTTGTCGACGGAGCTTAAACCAGCGGTCGGGTGG
**28a-sumo-F**	GCTCCGTCGACAAGCTTGCGGCCGCACTCGAGCACCACCAC
**28a-sumo-R**	GGATCCACCAATCTGTTCTCTGTGAGCCTCAATAATATCG
**N-VenA-F**	CAGATTGGTGGATCCGTGATCACCGACGTCGACCT
**N85A-F**	CCGTCAGCGGCCCGTAGATCCCTGCACGCTGACGCAGGAAGAACA
**N85A-R**	GCAGGGATCTACGGGCCGCTGACGG
**Y88A-F**	CGTACGGCACCGTCAGCGGCCCTGCGATCCCGTTACGCTGACGCA
**Y88A-R**	GCAGGGCCGCTGACGGTGCCGTACG
**Y88W-F**	CGTACGGCACCGTCAGCGGCCCCCAGATCCCGTTACGCTGACGCA
**Y88W-R**	TGGGGGCCGCTGACGGTGCCGTACG
**W107A-F**	CGATCACCGTGACGAACTGGTATGCGTCGGCGATGTTCTGGGCCCTGTCC
**W107A-R**	GCATACCAGTTCGTCACGGTGATCG
**Y108A-F**	AGCTGTCGATCACCGTGACGAACTGTGCCCAGTCGGCGATGTTCT
**Y108A-R**	GCACAGTTCGTCACGGTGAT
**S116A-F**	GTGCCGCCTCGTCGGAGACGAACGCGTCGATCACCGTGACGAACT
**S116A-R**	GCGTTCGTCTCCGACGAGGCGGCAC
**V111W-F**	AGACGAAGCTGTCGATCACCGTCCAGAACTGGTACCAGTCGGCGA
**V111W-R**	TGGACGGTGATCGACAGCTTCGTCT
**T112W-F**	CGGAGACGAAGCTGTCGATCACCCAGACGAACTGGTACCAGTCGG
**T112W-R**	TGGGTGATCGACAGCTTCGTCTCCG
**V111A-F**	AGACGAAGCTGTCGATCACCGTCGCGAACTGGTACCAGTCGGCGA
**V111A-R**	GCGACGGTGATCGACAGCTTCGT
**T112A-F**	CGGAGACGAAGCTGTCGATCACCGCGACGAACTGGTACCAGTCGG
**T112A-R**	GCGGTGATCGACAGCTTCGTCTC
**F185A-F**	TCTCCTCCGCGCACCCGCGCAGTGCCGCCTCGAGGGCCGCGACGAGCCG
**F185A-R**	GCACTGCGCGGGTGCGCGGAGGAGA

**F215A-F**	TGAGCAGTTCCAGGAACTCGCACCCTGCGCTGTCGACCCGCACCC
**F215A-R**	GCAGGGTGCGAGTTCCTGGA
**F219A-F**	CCGCGTACTCGGTGAGCAGTTCCAGTGCCTCGCACCCGAAGCTGT
**F219A-R**	GCACTGGAACTGCTCACCGA
**L220A-F**	CCGCGTACTCGGTGAGCAGTTCCGCGAACTCGCACCCGAAGCTGT
**L220A-R**	GCGGAACTGCTCACCGAGTACGC
**F338A-F**	AGTAGCGCGGCGTCAGATACTCTGCCTCCTGGCTTCCGCCGATCA
**F338A-R**	GCAGAGTATCTGACGCCGCGCTACT
**R211K-F**	ACTCGCACCCGAAGCTGTCGACTTTCACCCGCATGCACGCCTCGA
**R211K-R**	AAAGTCGACAGCTTCGGGTGCGAGT
**N256L-F**	CCTTGCGCCACGAGAGCAGGTCCAGGACGAGGATCAGCTGCCGCA
**N256L-R**	CTGGACCTGCTCTCGTGGCGCAAGG
**S260A-F**	GCTGGGCGTACTCCTTGCGCCATGCGAGCAGGTCGTTGACGAGGA
**S260A-R**	GCATGGCGCAAGGAGTACGCCCAGC
**E264L-F**	TCATGGTGTCCCGCTGGGCGTACAGCTTGCGCCACGAGAGCAGGT
**E264L-R**	CTGTACGCCCAGCGGGACACCATGA
**R344K-F**	ACACCGAGCCGTCGCCGAAGTATTTCGGCGTCAGATACTCGAACT
**R344K-R**	AAATACTTCGGCGACGGCTCGGTGT
**Y345F-F**	CCCACACCGAGCCGTCGCCGAAAAAGCGCGGCGTCAGATACTCGA
**Y345F-R**	TTTTTCGGCGACGGCTCGGTGTGGG
**Y345L-F**	CCCACACCGAGCCGTCGCCGAACAGGCGCGGCGTCAGATACTCGA
**Y345L-R**	CTGTTCGGCGACGGCTCGGTGTGGG
**V111A/T112A-F**	CGCCGCGAACTGGTATGCGTCGGCGATGTTCTGGGCCCTGTC
**V111A/T112A-R**	CGCCGACGCATACCAGTTCGCGGCGGTGATCGACAGCTTCGTCTC
**F215A/F219A-F**	GCAGTTCCAGTGCCTCGCACCCTGCGCTGTCGACCCGCACCCGCA
**F215A/F219A-R**	GCAGGGTGCGAGGCACTGGAACTGC

Supplementary Figures

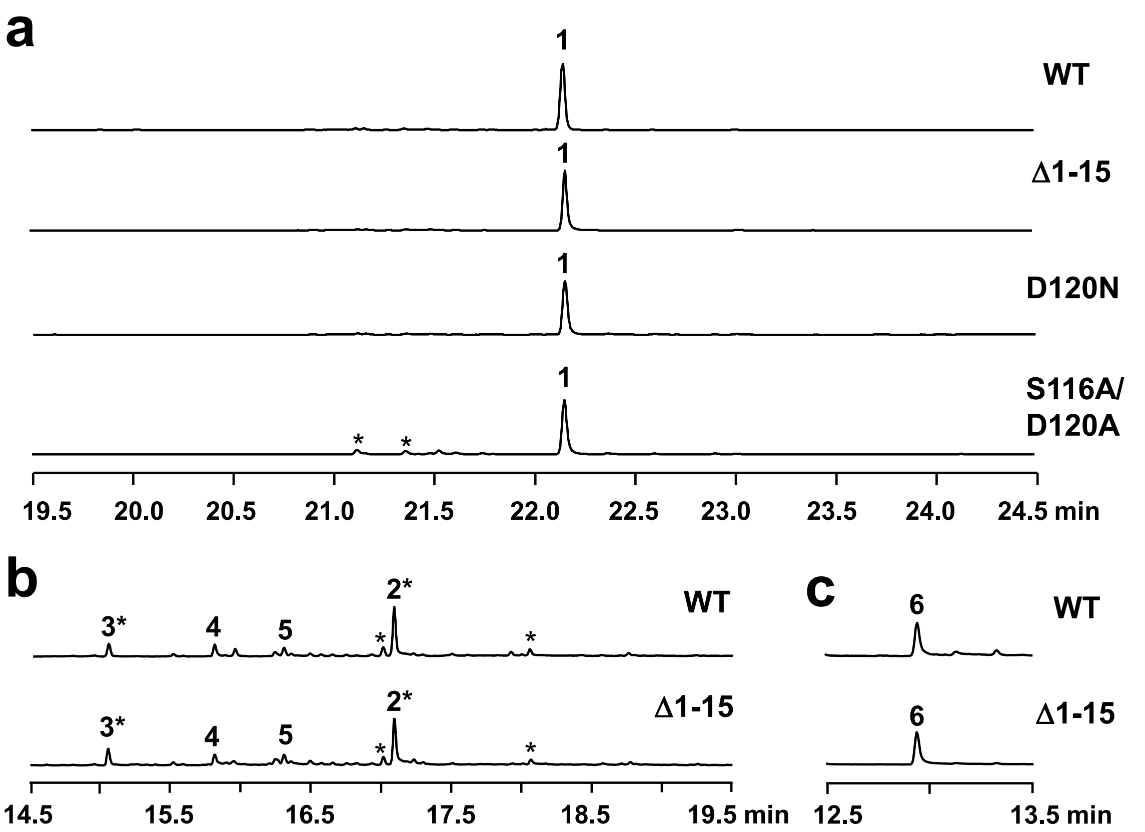

Supplementary Figure 1. GC-MS (total ion chromatograms) analysis of the diterpene (**a**), sesquiterpene (**b**) and monoterpene products (**c**) of VenA wild type and mutants. The asterisks stand for the uncharacterized products, the ratios of which to total products are > 5%.

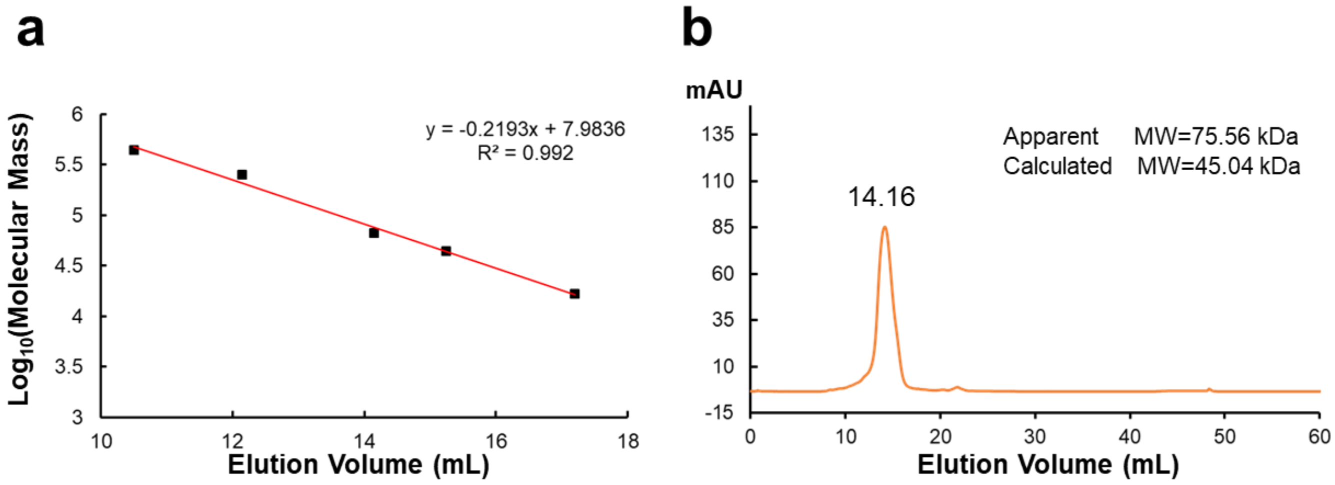

Supplementary Figure 2. Gel filtration analysis for oligomeric state determination of VenA. **a**, The standard curve of gel filtration chromatography. **b**, The calculation of the apparent molecular mass of VenA. Experimentally, purified VenA was applied to size-exclusion chromatography using Superdex^{TM} 200 increase 10/300 GL column (GE Healthcare) in a buffer containing 50 mM Tris-HCl, 300 mM NaCl, pH = 8.0. The apparent molecular mass of each sample was calculated based on the calibration of the column using protein standards.

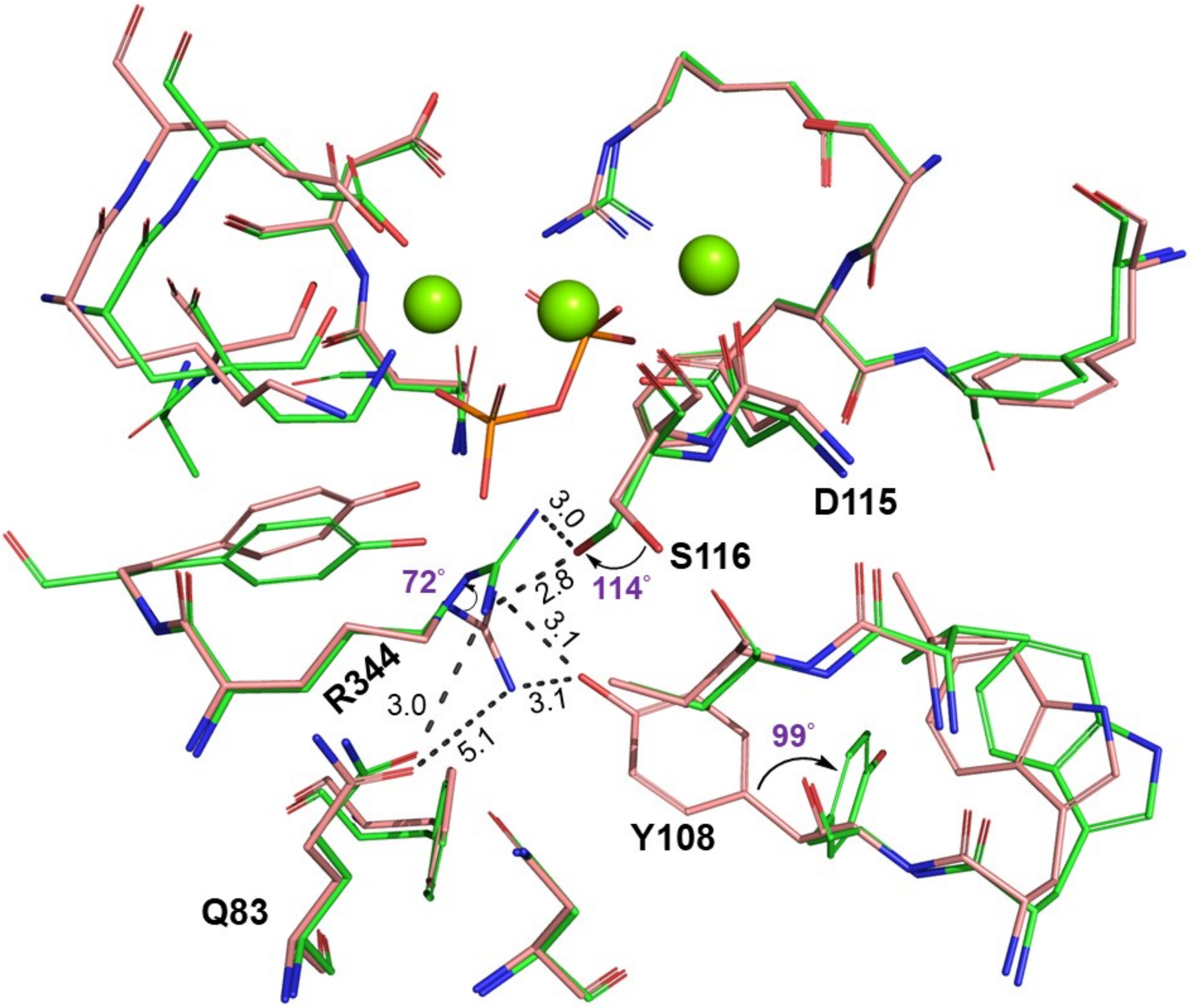

Supplementary Figure 3. The comparison of the residues in active sites of the *apo* form (pink) and PP_i-Mg^{2+}-bound form (green) of VenA. The distances between different atoms are shown in angstrom.

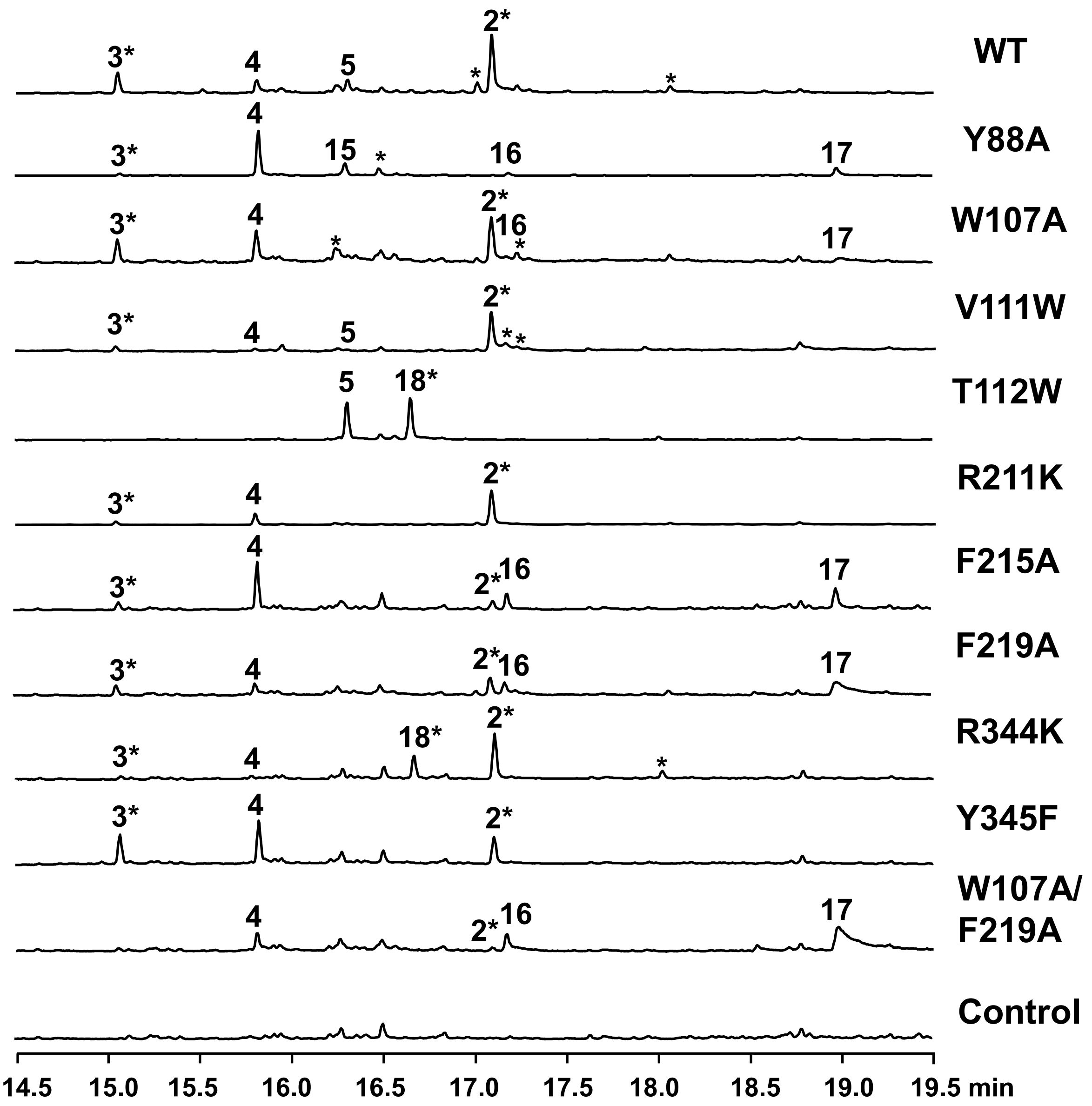

Supplementary Figure 4. GC-MS (total ion chromatograms) analysis of the sesquiterpene products of VenA wild type and representative mutants. The VenA mutants which share the same product profiles with that of wild type enzyme are not shown. The asterisks stand for the uncharacterized sesquiterpenes (> 5% of total products). The injected germacradien-6-ol (**18**) was converted into shyobunol (**18***) by Cope rearrangement during GC-MS analysis.

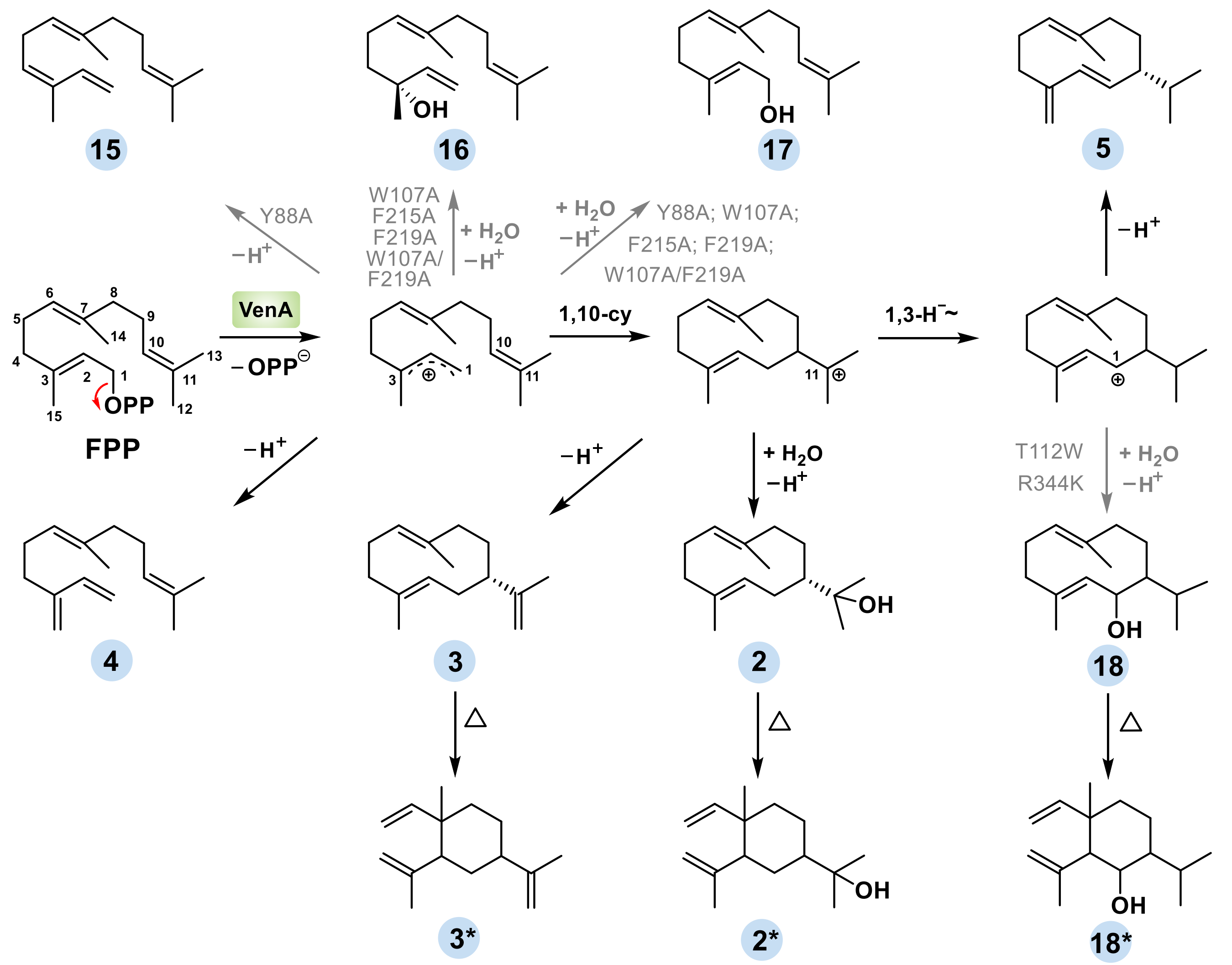

Supplementary Figure 5. The proposed biosynthetic mechanisms of the sesquiterpene/sesquiterpenoids produced by VenA wild type and mutants.

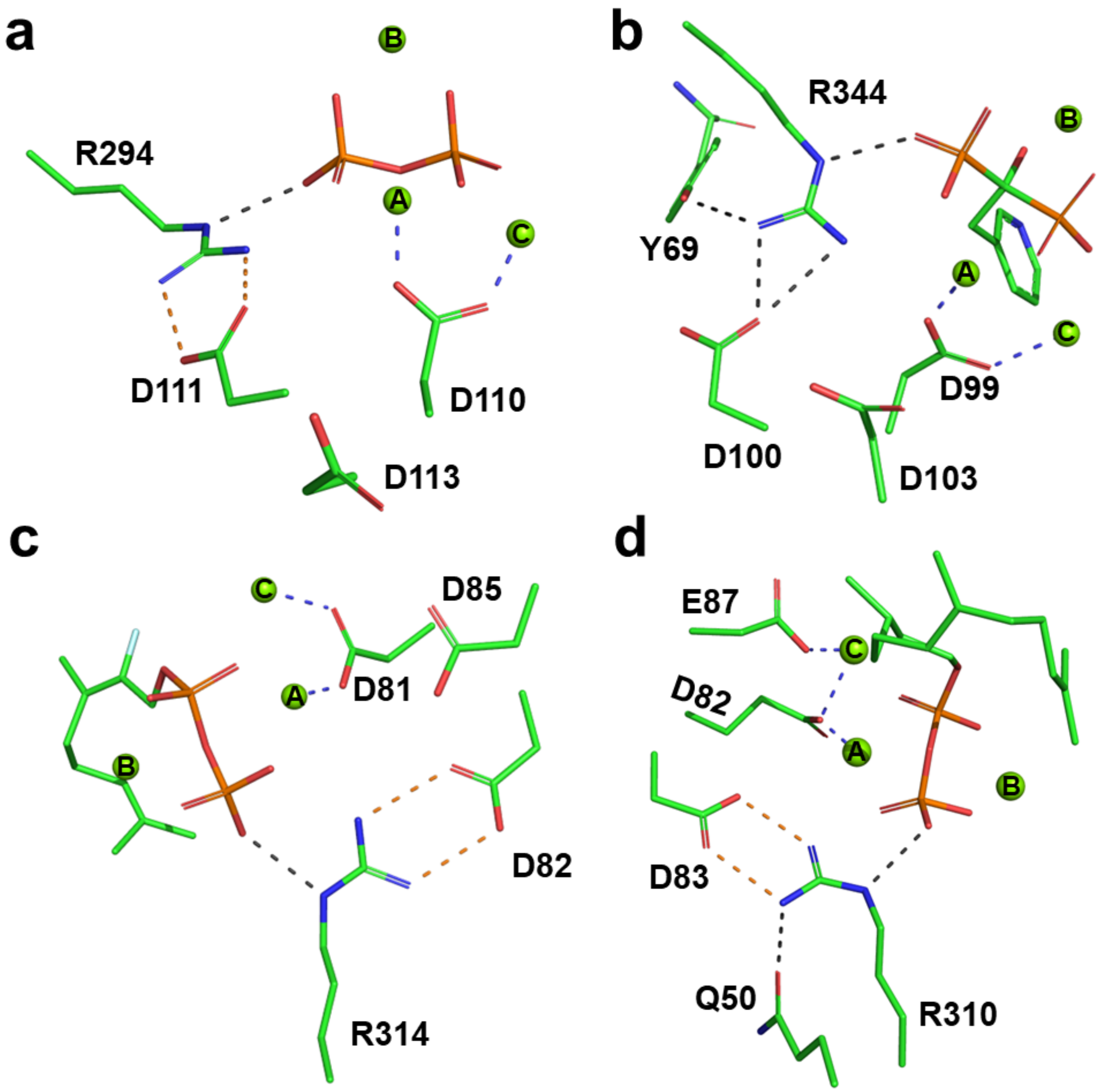

Supplementary Figure 6. The Asp-rich motifs of the selected bacterial type I TPSs. **a**, CotB2 from *Streptomyces melanosporofaciens* (PDB ID: 6GGI). **b**, EIZS from *Streptomyces coeicolor* (PDB ID: 7KJ9). **c**, 1,8-cineole synthase from *Streptomyces clavuligerus* (PDB ID: 5NX7). **d**, Selinadiene synthase from *Streptomyces pristinaespiralis* (PDB ID: 4OKZ). Only the closed forms bound with trinuclear magnesium cluster are selected. The third Asp in the Asp-rich motif of CotB2, EIZS and 1,8-cineole synthase is located outside the active pocket. Mg^{2+} ions are marked by green spheres; the salt bridge, coordination and hydrogen bonds are marked by orange, blue and black dashed lines, respectively. All the residues binding with conserved Arg of RY dimer were shown.

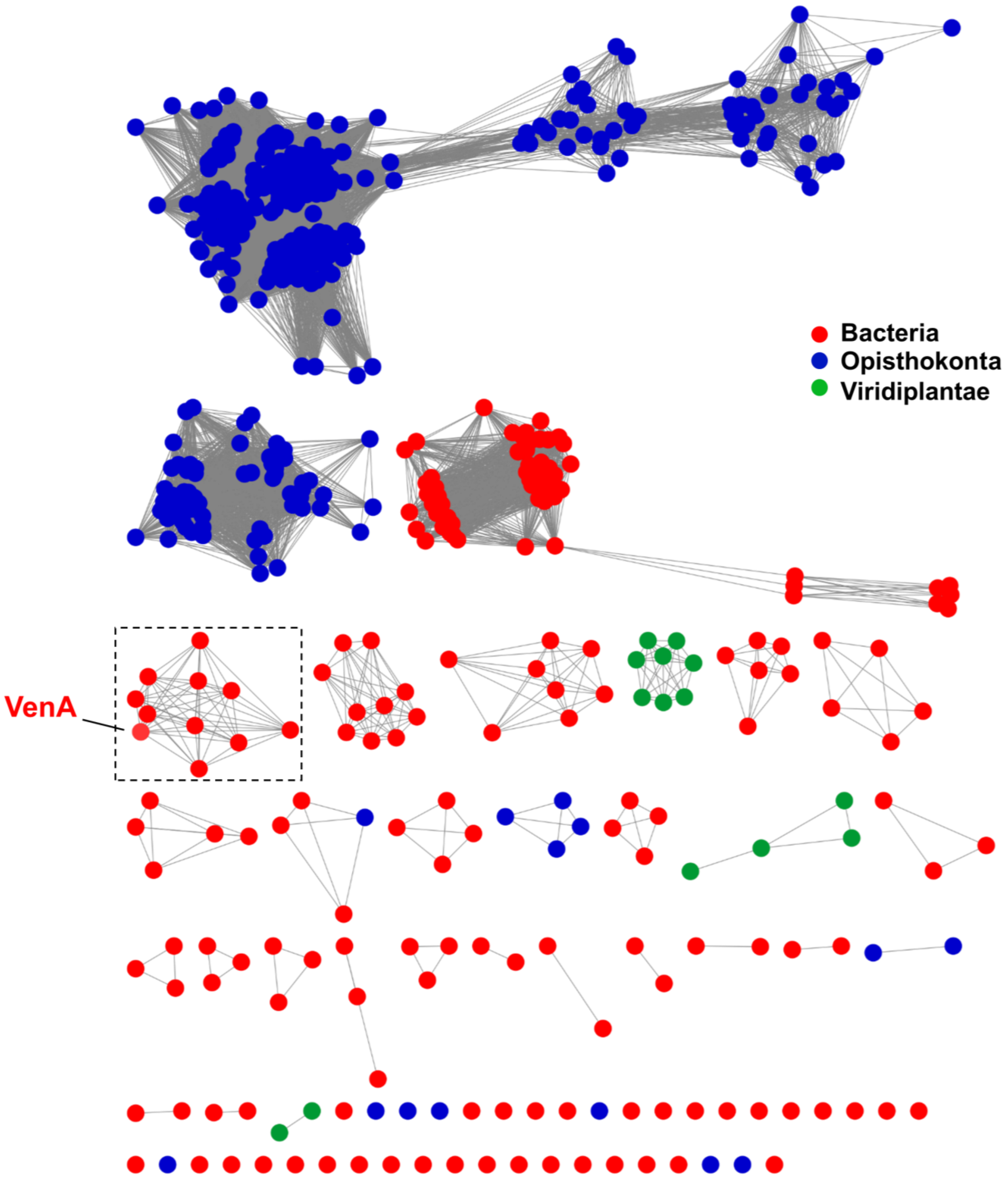

Supplementary Figure 7. The sequence similarity network (SSN) of VenA. The SSN was generated using the EFI-Enzyme Similarity Tool with default settings using VenA as a query sequence to search the homologues in UniProt database. The alignment score threshold was set as 70. The potential venezuelaene A synthases from bacteria are marked with a dashed box.

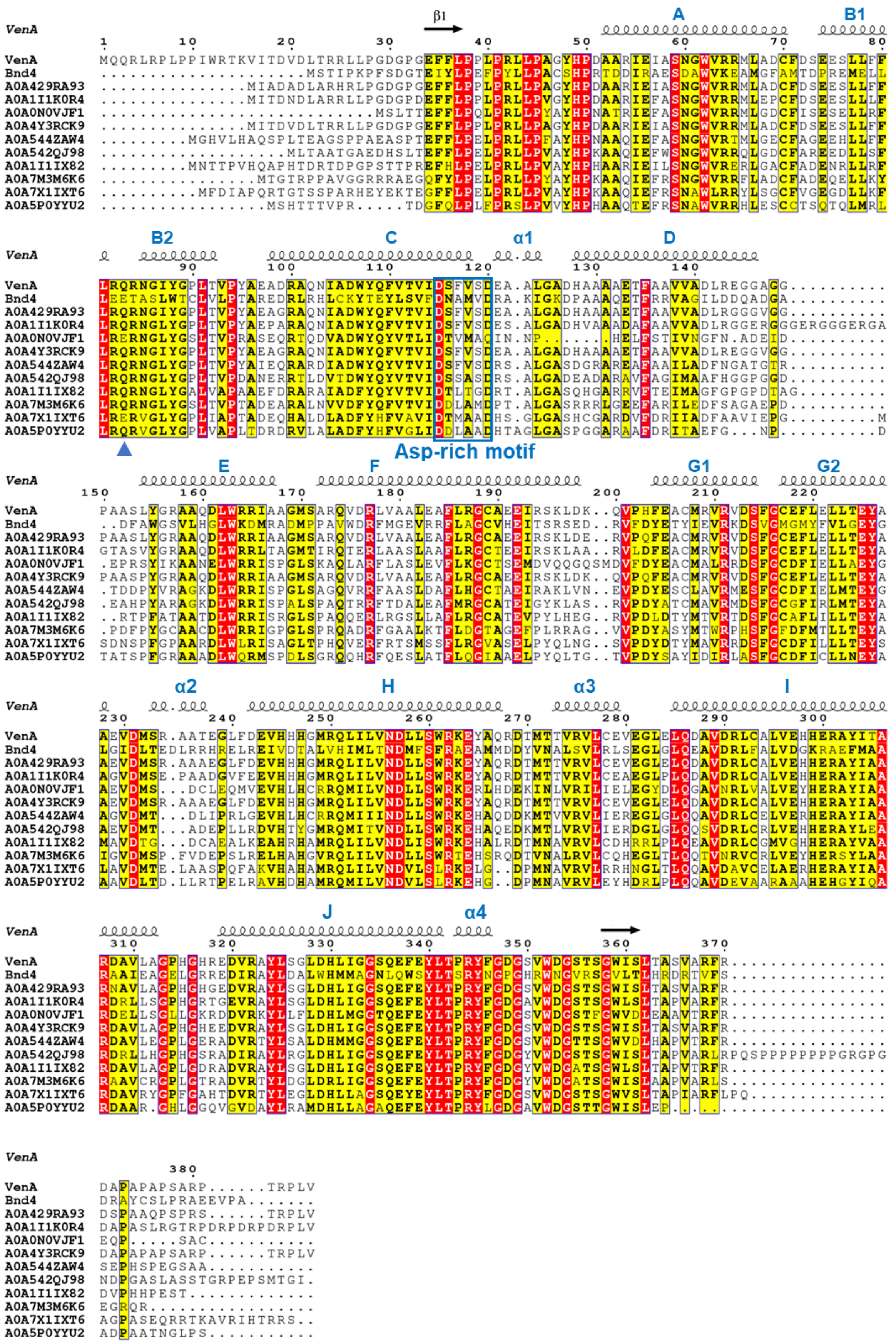

Supplementary Figure 8. The protein sequence alignment of VenA, Bnd4 and VenA's homologues collected from SSN analysis (Supplementary Fig. 7).

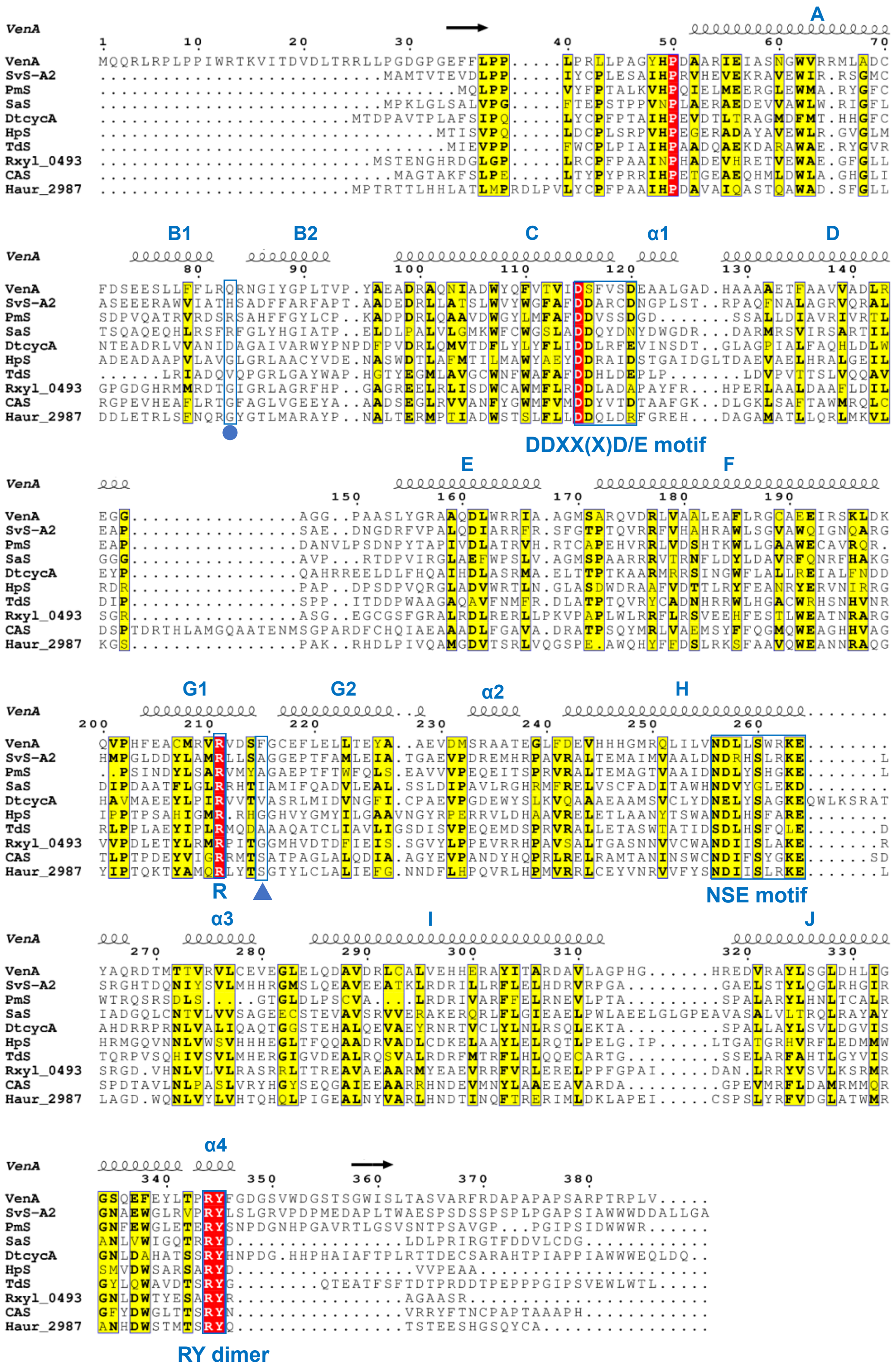

Supplementary Figure 9. The protein sequence alignment of VenA with nine type I bacterial DTS including SvS-A2 (GenBank accession number: 6TBD_A), PmS (WP_030431358.1), SaS (WP_030426588.1), DtcycA (M1V9Q0.1), HpS (WP_003963279.1), TdS (EIF90392.1), Rxyl_0493 (WP_041328593.1), CAS (WP_030430753.1) and Haur_2987 (WP_012190524.1). The residues Gln83 and Phe215 in G1/2 kink of VenA are marked by "●" and "▲", respectively.

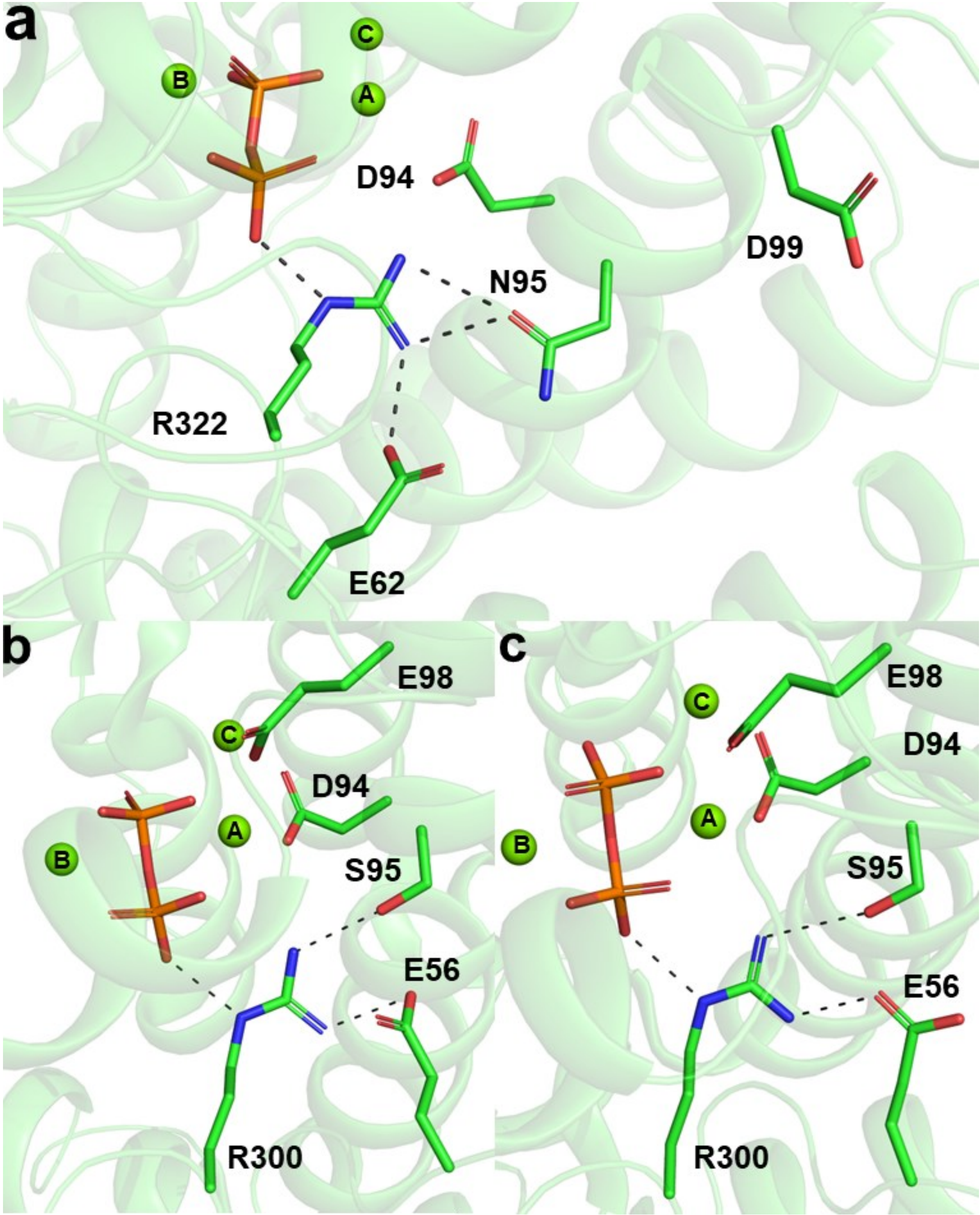

Supplementary Figure 10. The active site of the AlphaFold2 structure model of Bnd4 (**a**) and the chain A (**b**) and chain B (**c**) of FgGS structure (PDB ID: 6VYD). The distances (Å) between different atoms are shown in angstrom and the distances < 3.4 Å are considered for the formation of hydrogen bonds.

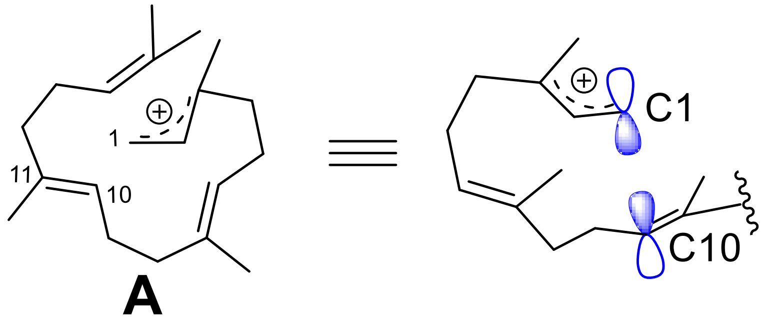

Supplementary Figure 11. The orientations of *p*-orbitals between C1 and C10 of the intermediate **A** (Fig. 5).

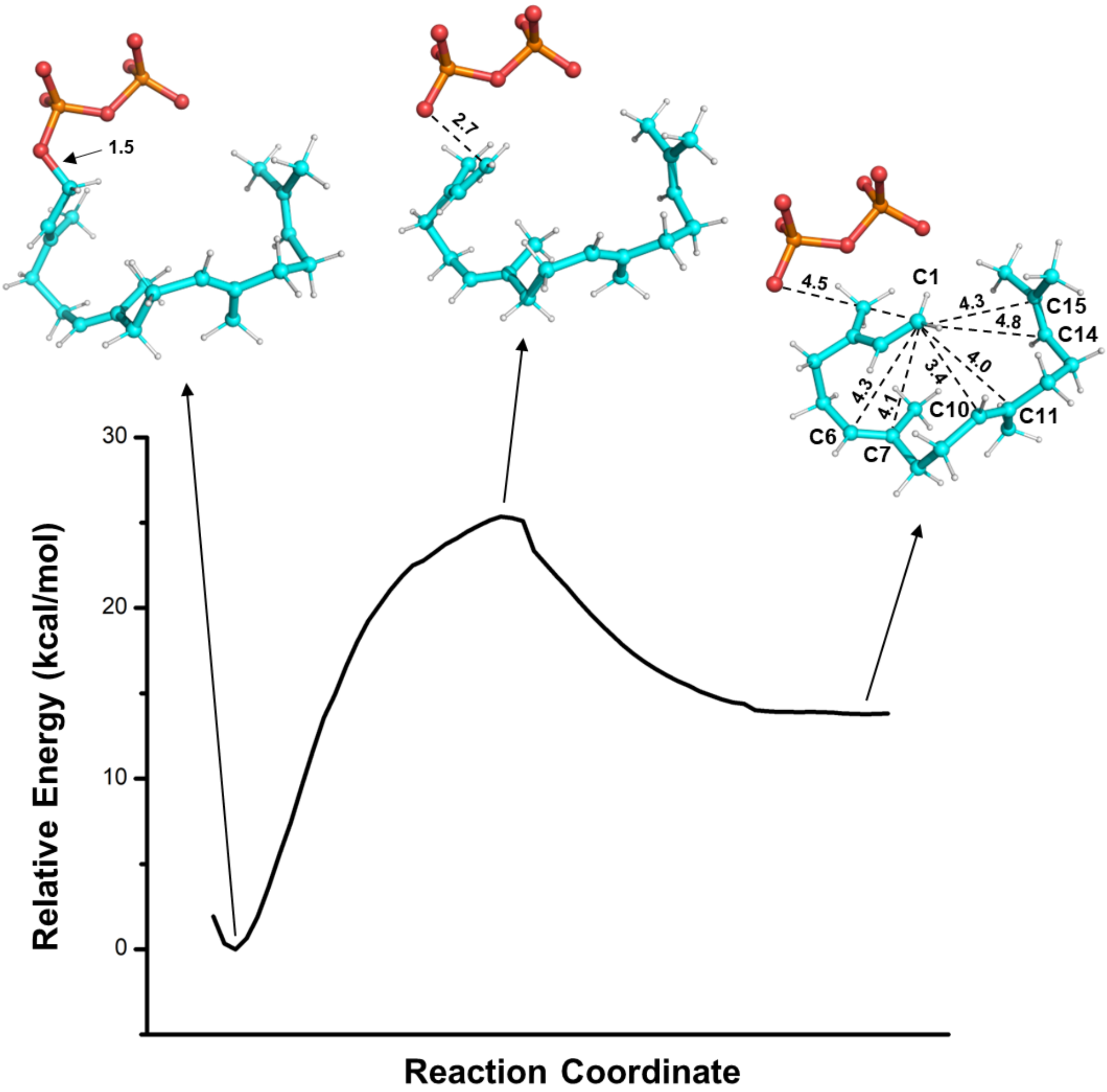

Supplementary Figure 12. The potential energy profile and key structures of the PP_i cleavage for GGPP in VenA. The reaction coordinate is along the C1-O1 bond. The distances are given in Å.

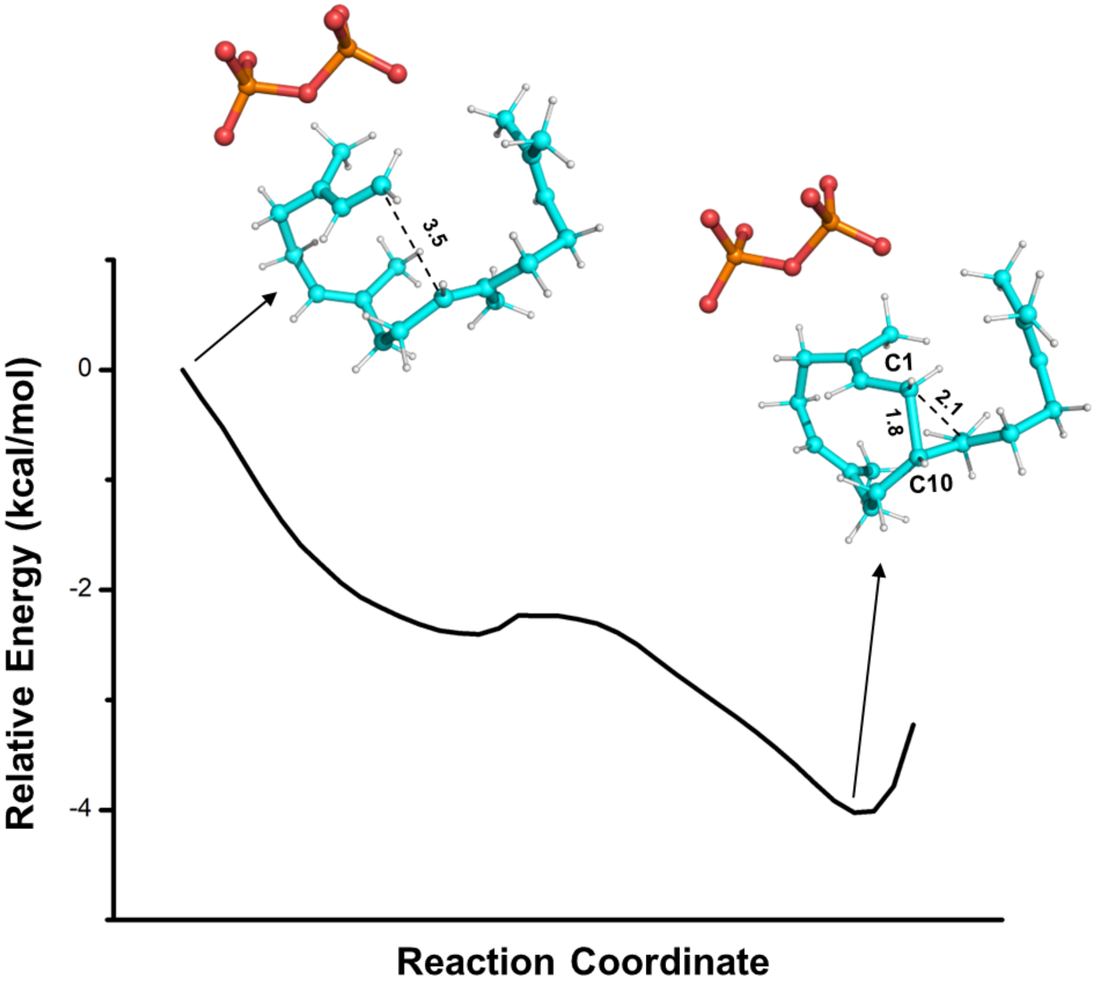

Supplementary Figure 13. The potential energy profile and key structures of the 1,10-cyclizaiton for GGPP in VenA. The distances are given in Å.

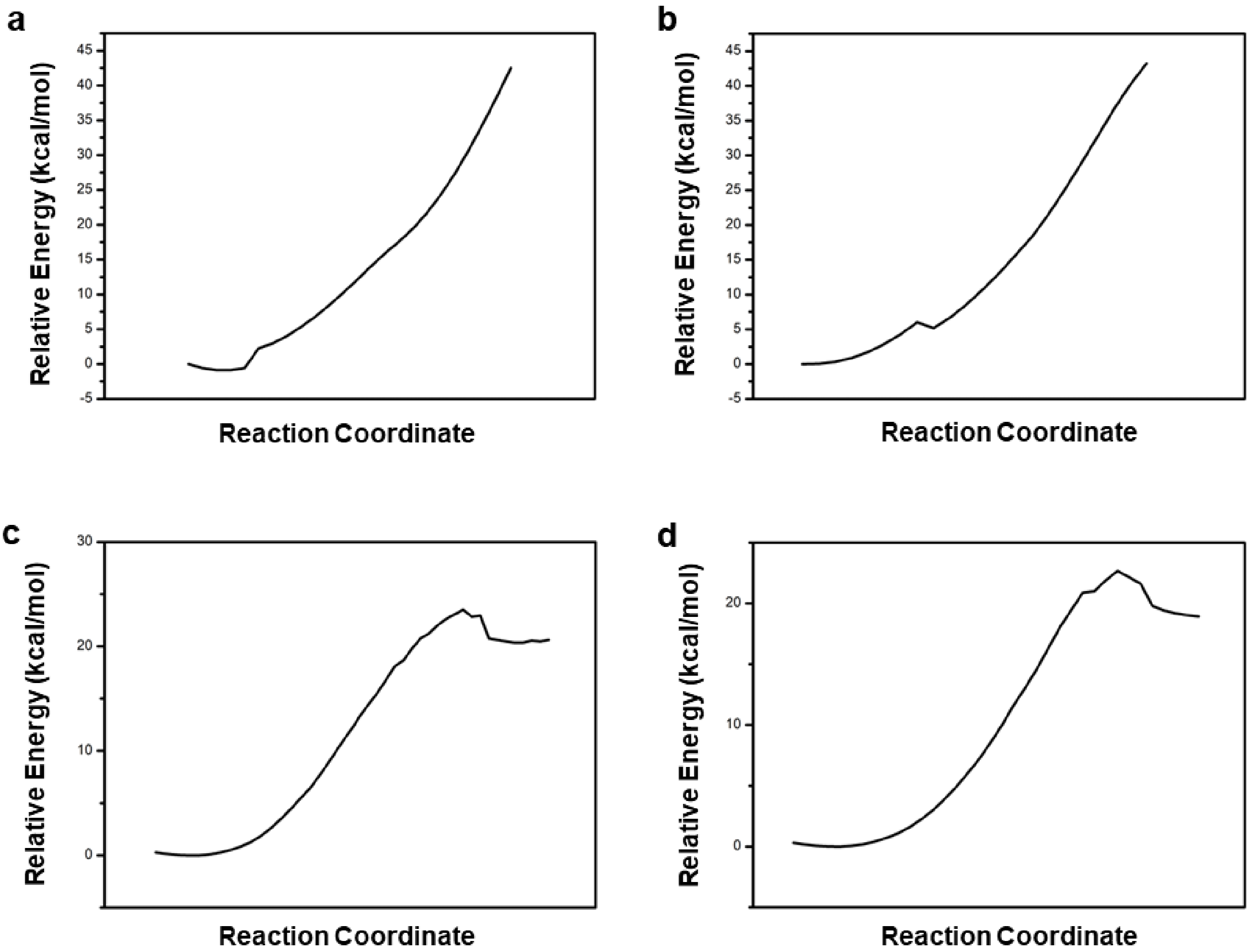

Supplementary Figure 14. The potential energy profiles of various cyclization styles for GGPP. **a**, 1,6- cyclization. **b**, 1,7-cyclization. **c**, 1,14-cyclization. **d**, 1,15-cyclization.

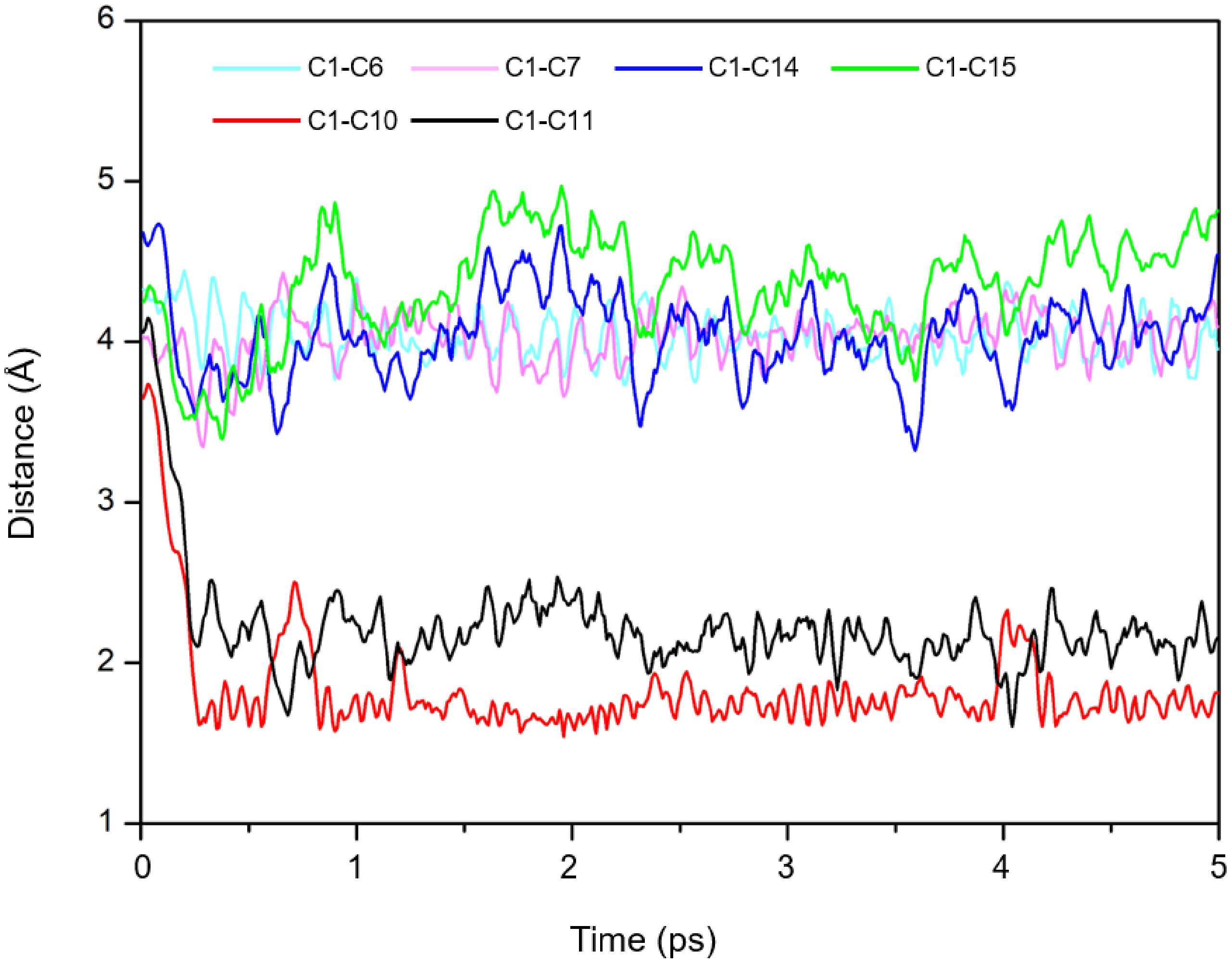

Supplementary Figure 15. The selected distance evolutions along the 5 ps QM/MM MD trajectories for intermediate **A**. The distances are given in Å.

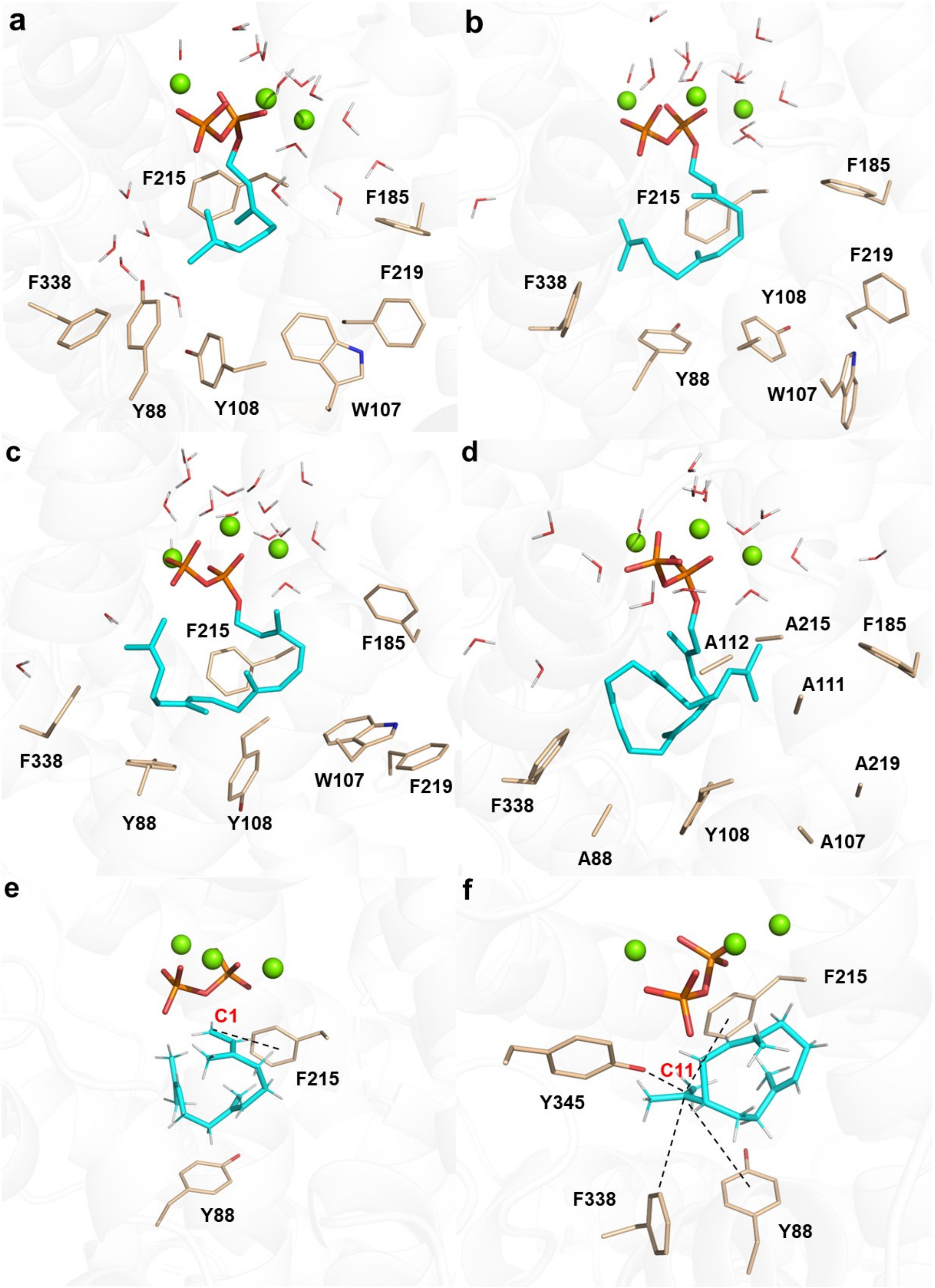

Supplementary Figure 16. a, The QM/MM models and zoom-in active sites of VenA with GPP (**a**), FPP (**b**) or GGPP (**c**) binding and of VenA$^{Y88A/W107A/V111A/T112A/F215A/F219A}$ with GFPP (**d**) binding. **e**, QM/MM predicted pose of farnesyl cation. **f**, QM/MM predicted pose of 11-germacryl cation. The aromatic amino acid residues around substates and the water molecules around the active pocket of wild-type or mutant VenA (**a**-**d**) are shown. The proposed key interactions between carbon cation intermediates and VenA residues are shown by dashed lines.

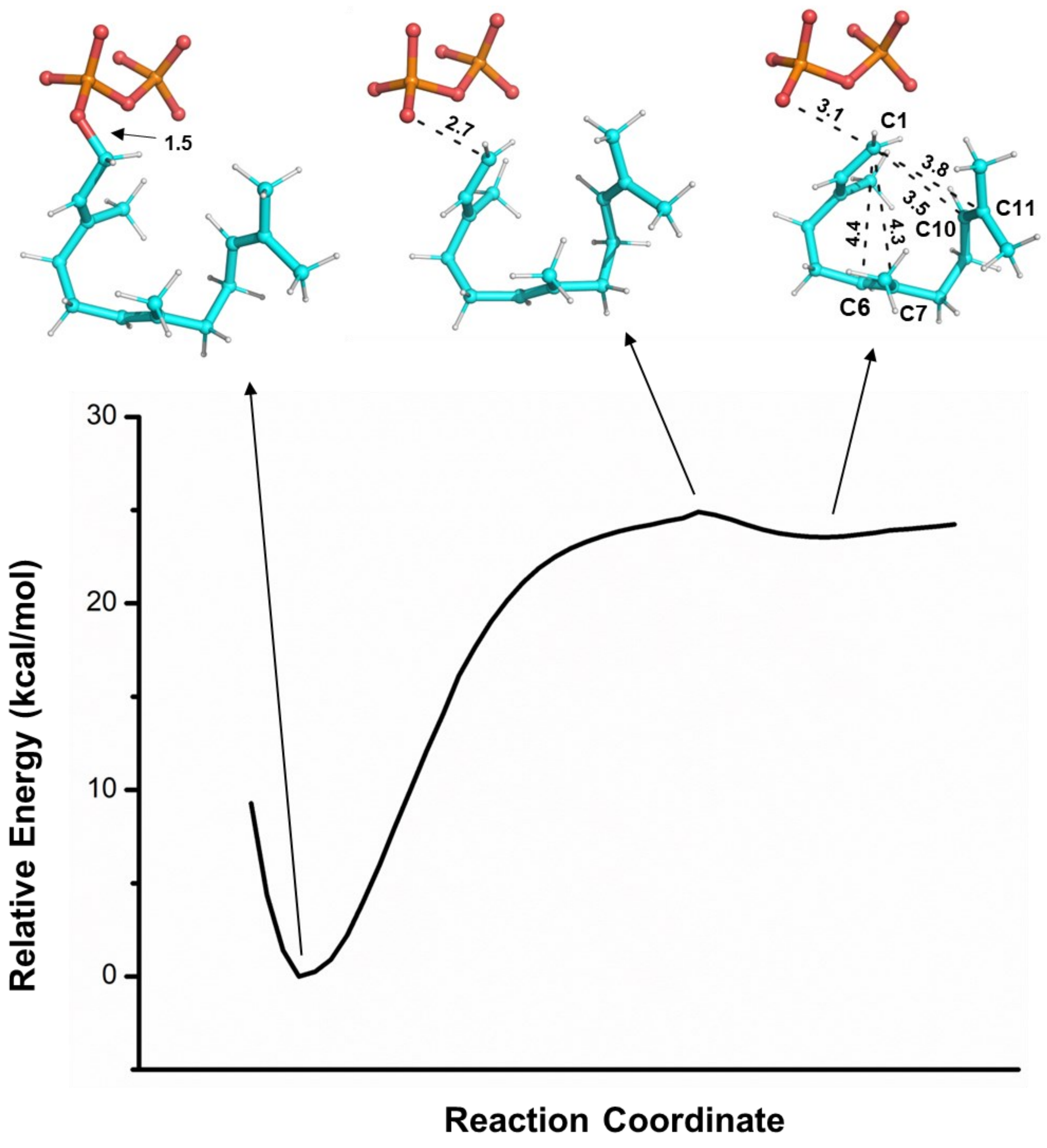

Supplementary Figure 17. The potential energy profile and key structures of the PP_i cleavage for FPP in VenA. The distances are given in Å.

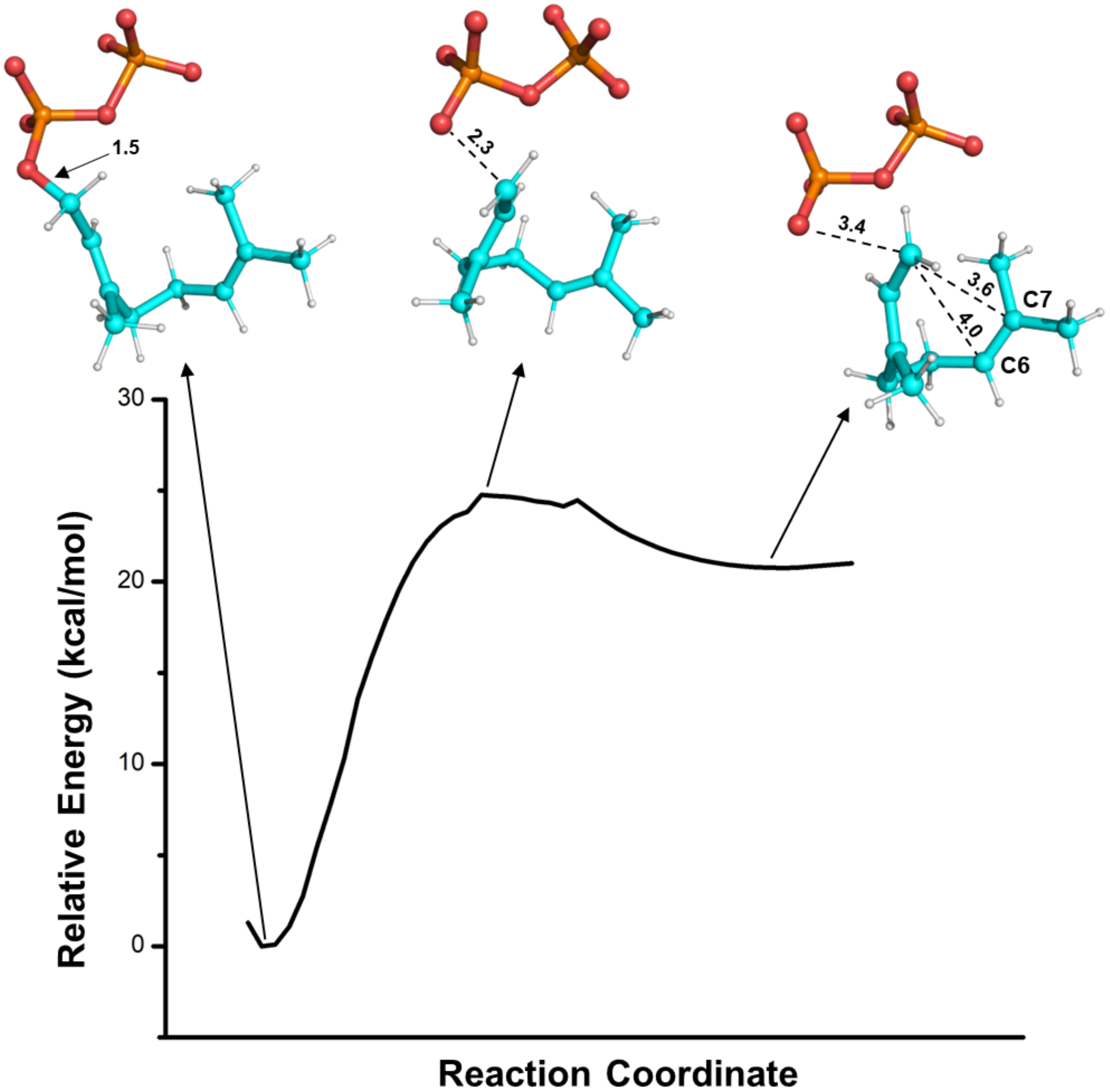

Supplementary Figure 18. The potential energy profile and key structures of the PP_i cleavage for GPP in VenA. The distances are given in Å.

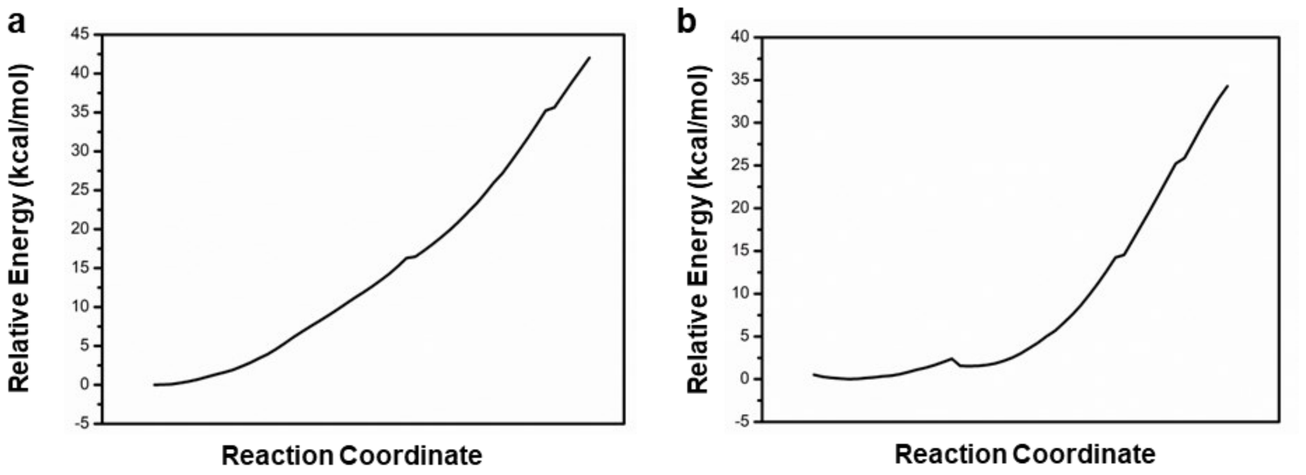

Supplementary Figure 19. The potential energy profiles of various cyclization styles for FPP. **a**, 1,6-cyclization. **b**, 1,7-cyclization.

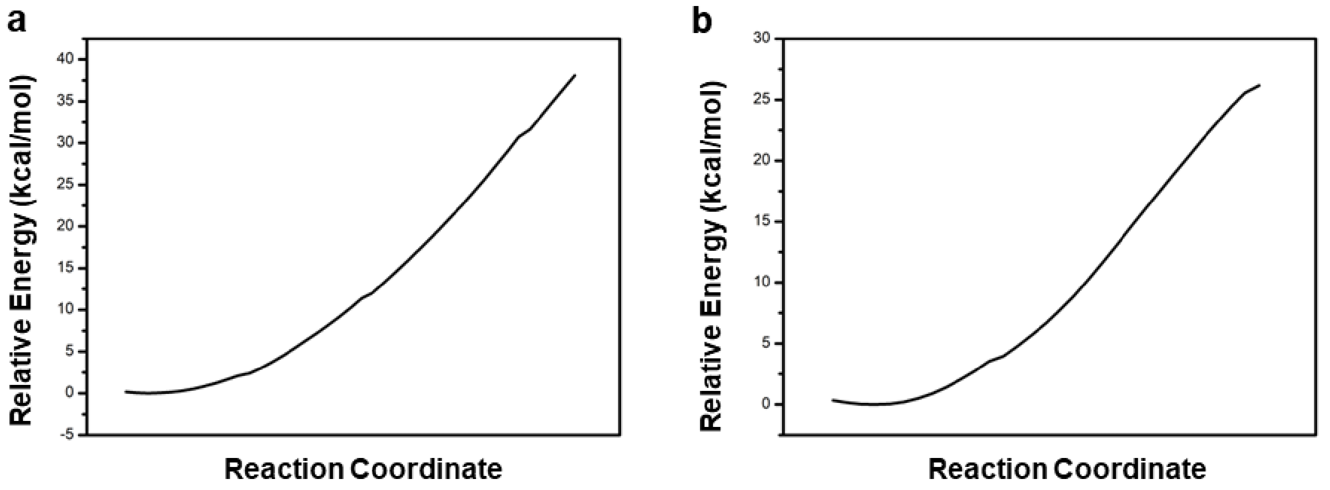

Supplementary Figure 20. The potential energy profiles of various cyclization styles for GPP. **a**, 1,6-cyclization. **b**, 1,7-cyclization.

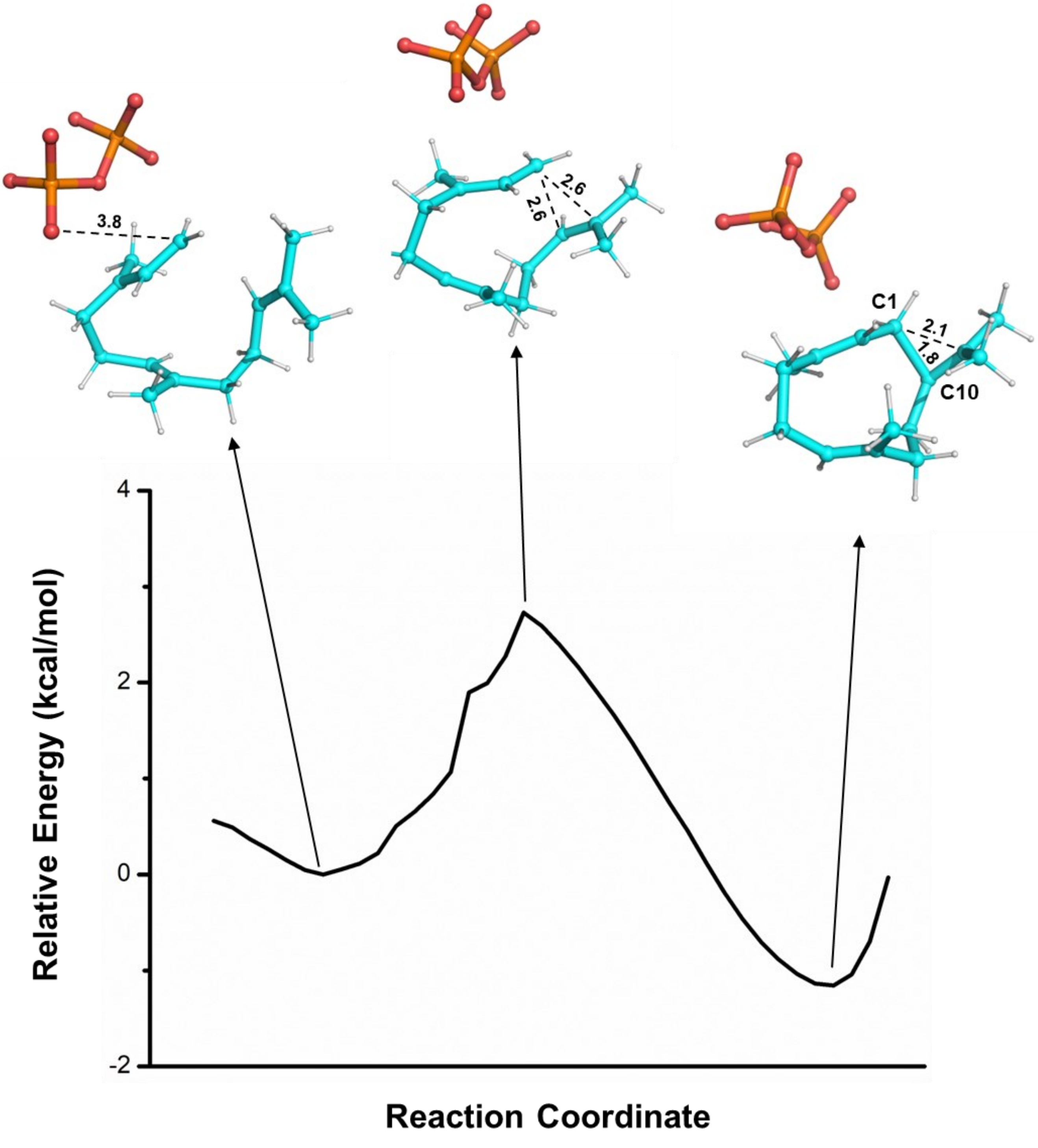

Supplementary Figure 21. The potential energy profile and key structures of the 1,10-cyclization for FPP in VenA. The distances are given in Å.

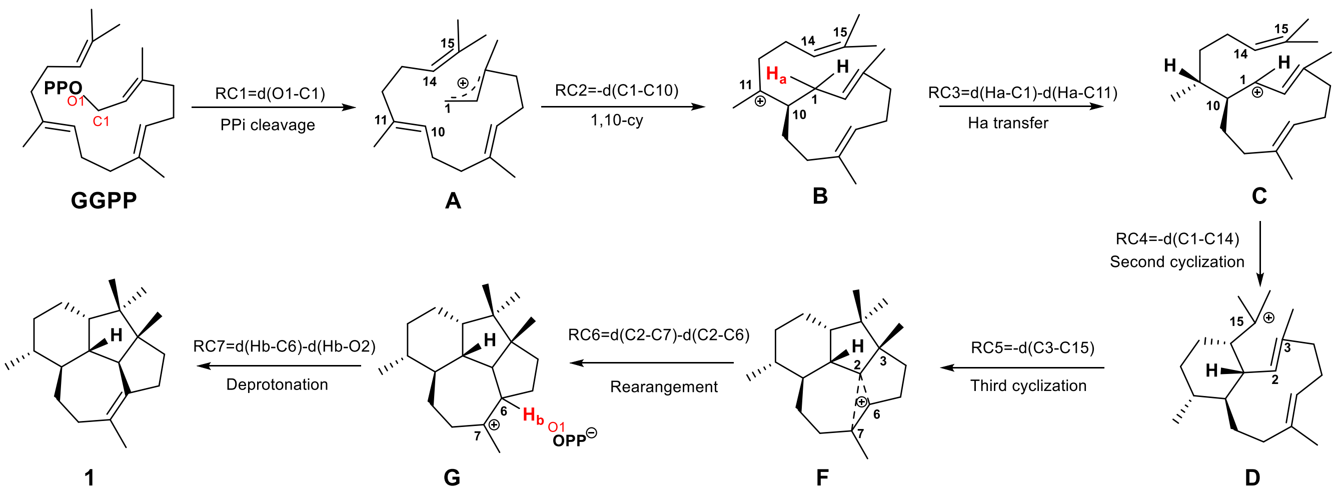

Supplementary Figure 22. The defined reaction coordinates (RC) for biosynthesis of **1** by VenA.

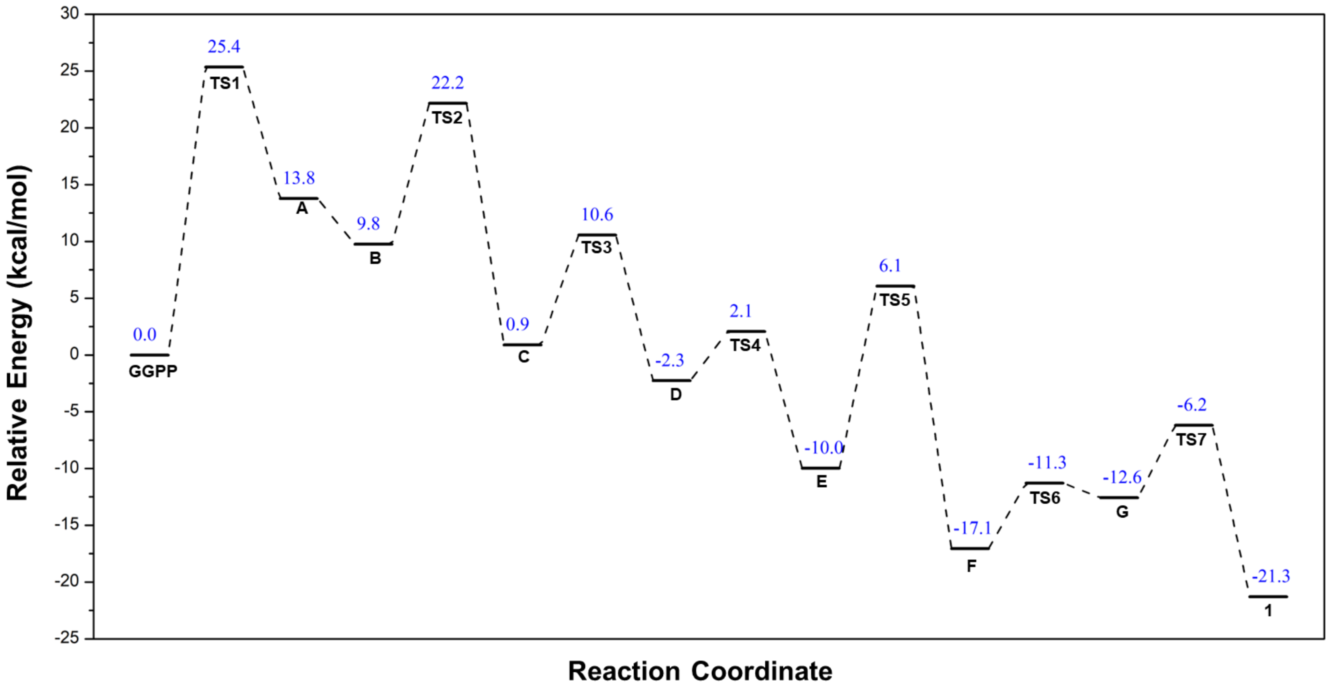

Supplementary Figure 23. The energy profile for biosynthesis of **1** by VenA.

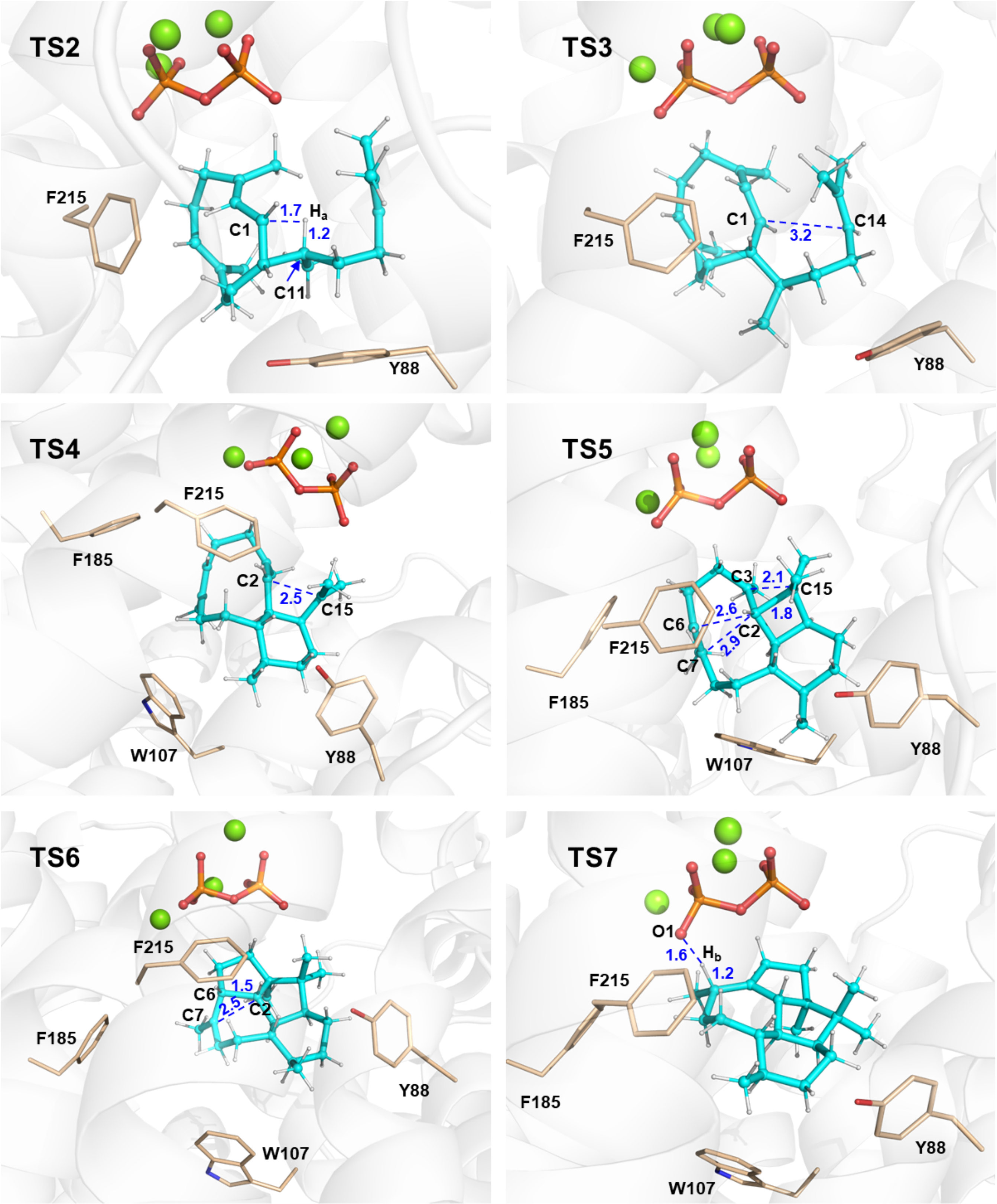

Supplementary Figure 24. The QM/MM models of TS2-TS7 from intermediate **B** to product **1**.

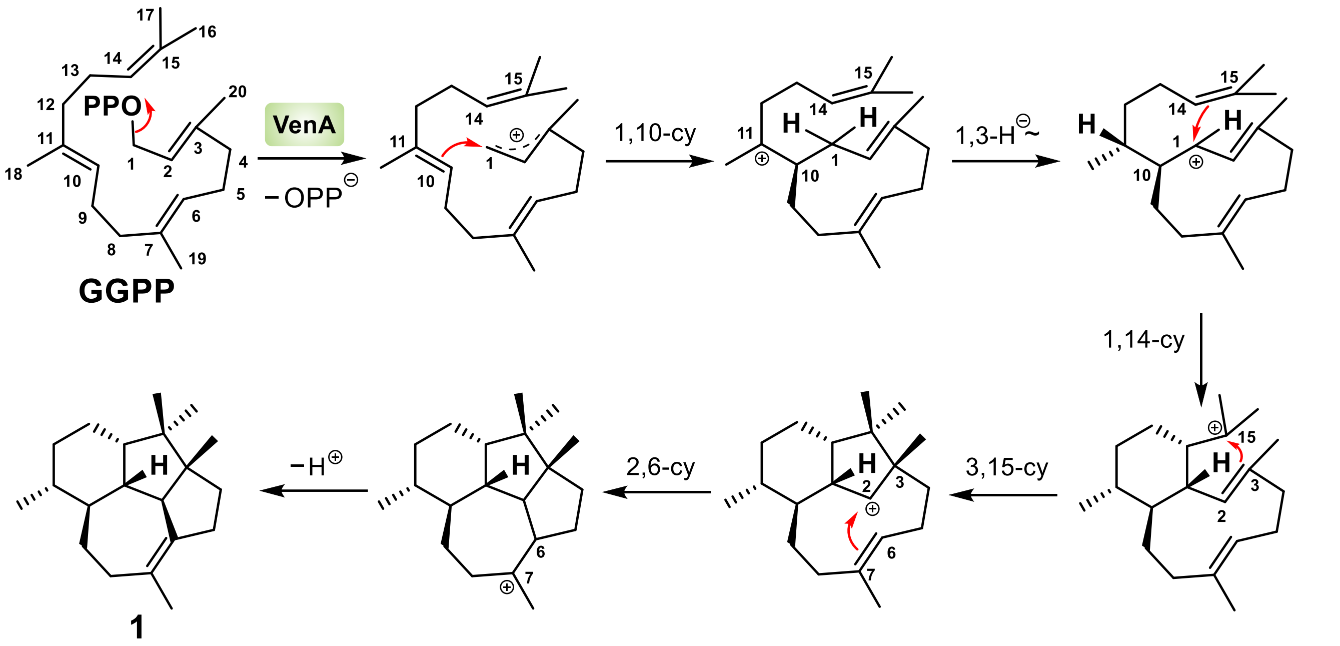

Supplementary Figure 25. Previous proposed biosynthetic mechanism of **1**[11]. The 3,15-cyclization step was confirmed to be unfavourable and C2 cation intermediate is quite unstable according to QM/MM calculations. Therefore, the old mechanism was revised in this study (see Fig. 5).

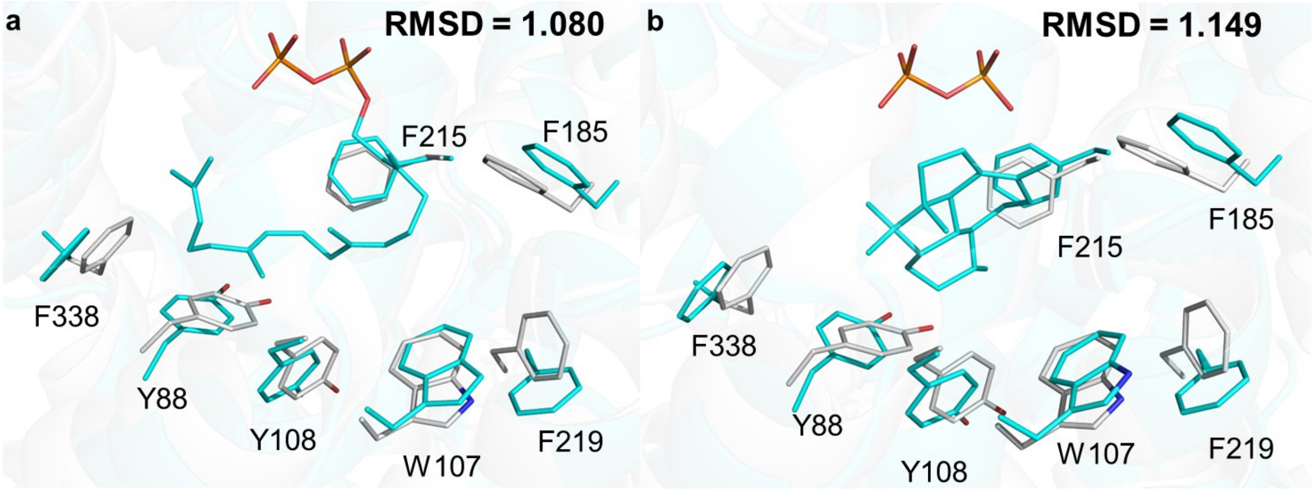

Supplementary Figure 26. Superimposition of the active pockets of the X-ray structure and multiscale simulation models: substrate (**a**) and product (**b**) states. Residues from the crystal structure and simulation models are shown in grey and cyan, respectively. The RMSD values are 1.080 Å and 1.149 Å, respectively.

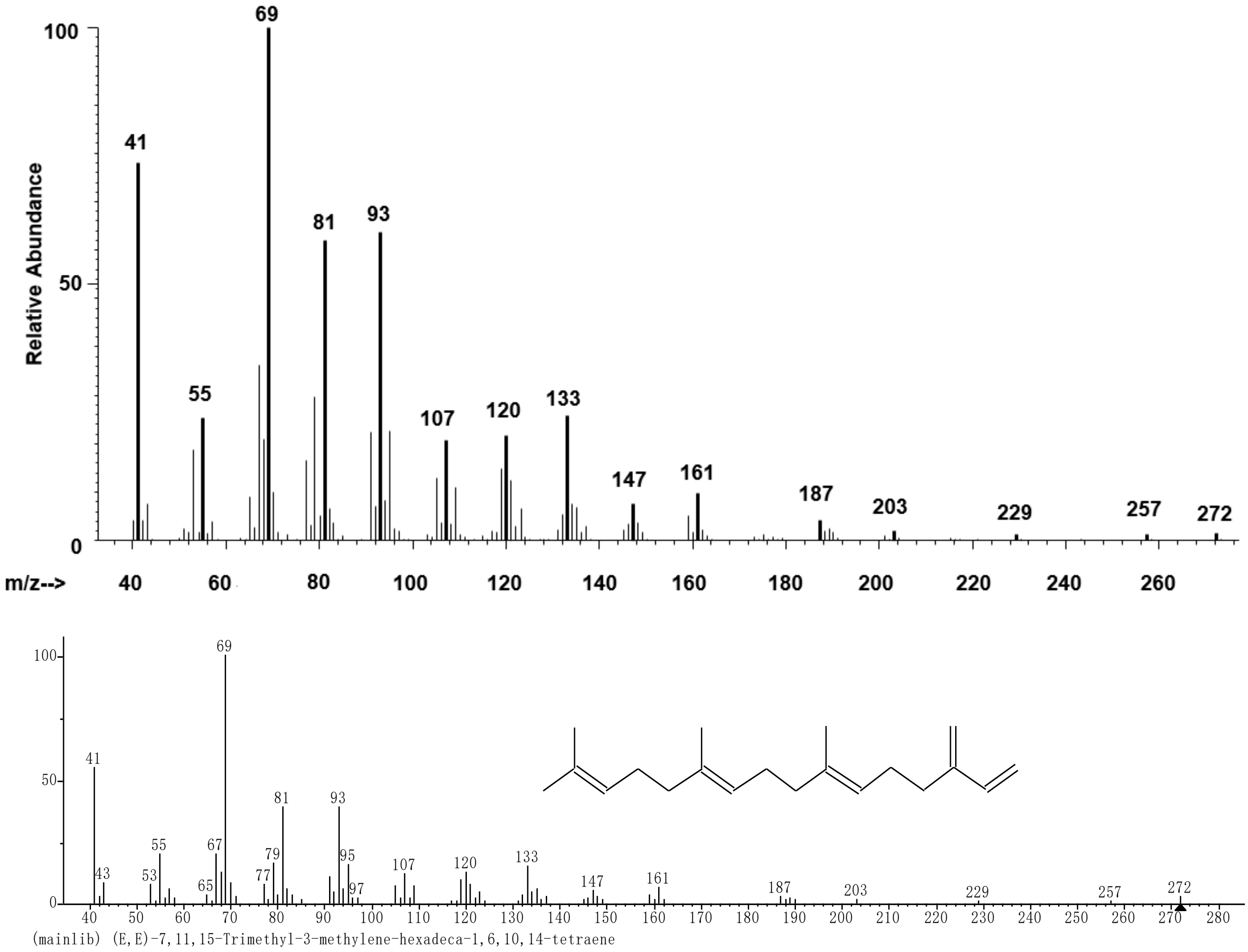

Supplementary Figure 27. The GC mass spectrum of **7** (*top*) and the standard mass spectrum of *β*-springene in the NIST library (*bottom*).

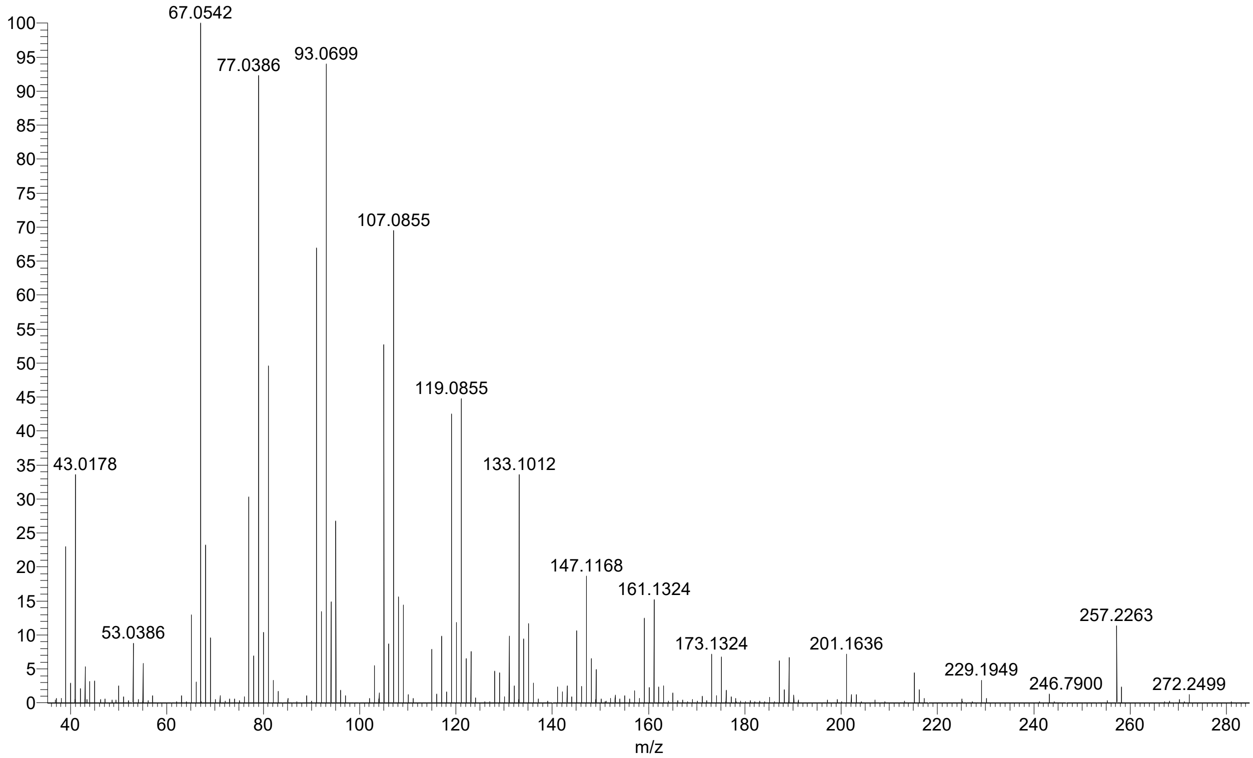

Supplementary Figure 28. The high-resolution GC mass spectrum of (*S*)-cembrene A (**8**) (*calc.* 272.2499; *obs.* 272.2499).

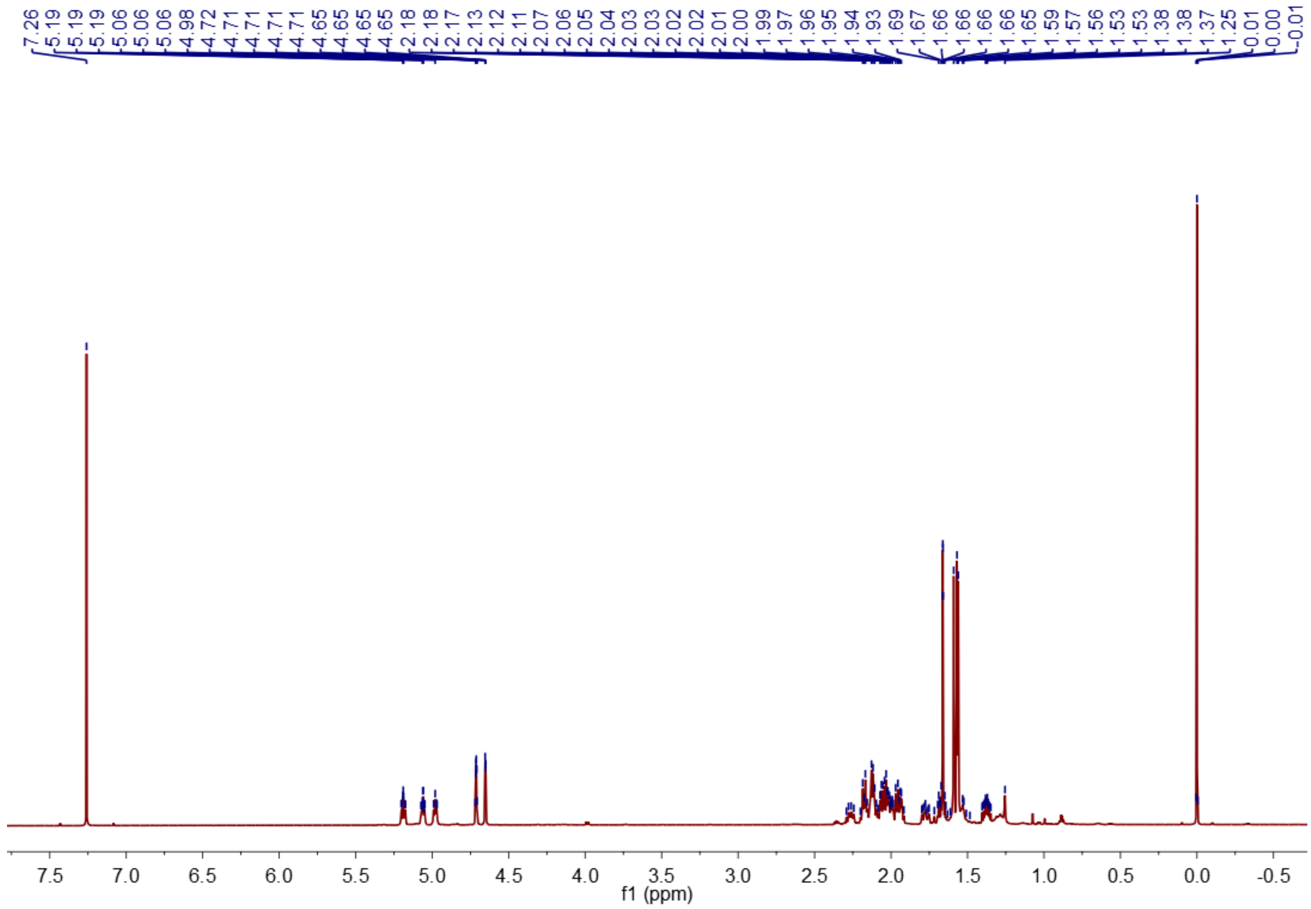

Supplementary Figure 29. The ^1H NMR spectrum of **8** in $CDCl_3$.

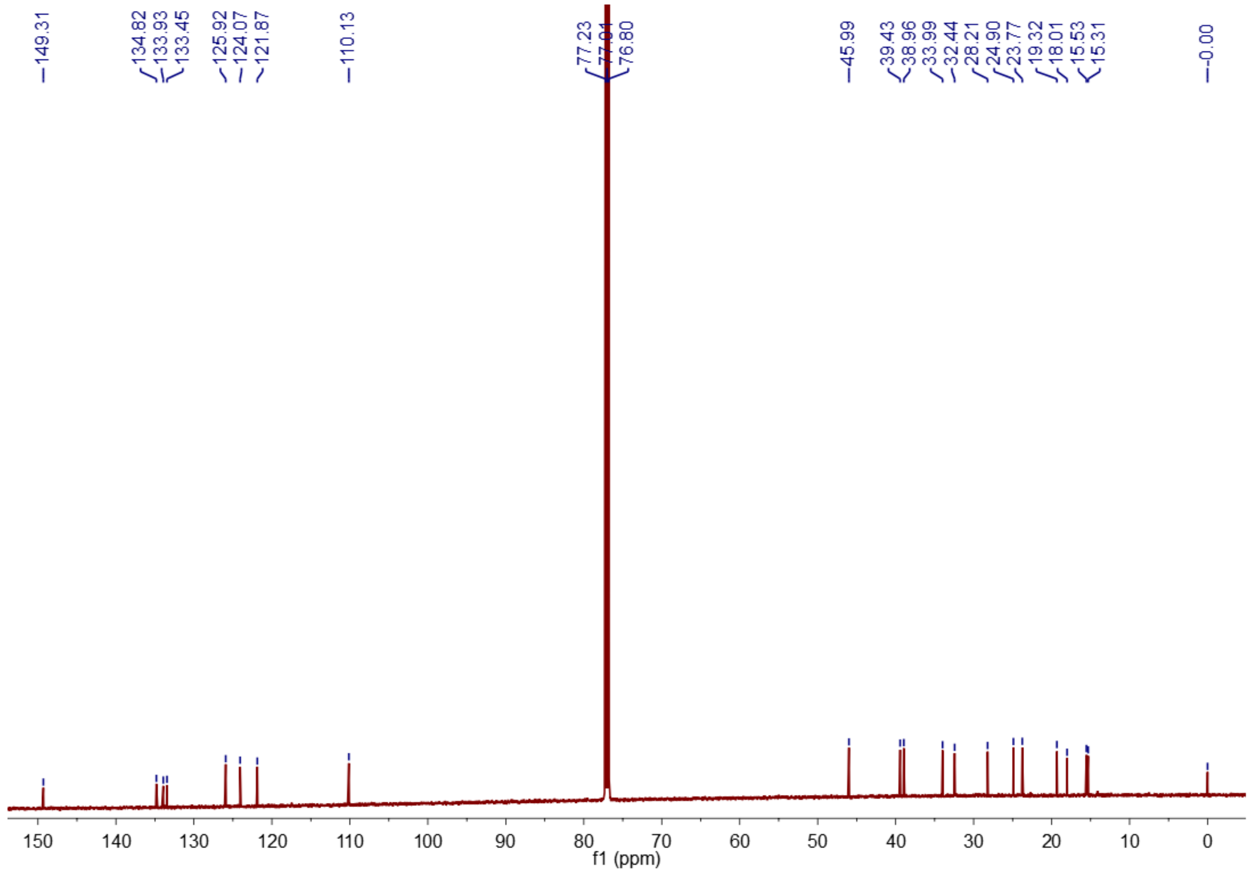

Supplementary Figure 30. The ^{13}C NMR spectrum of **8** in $CDCl_3$.

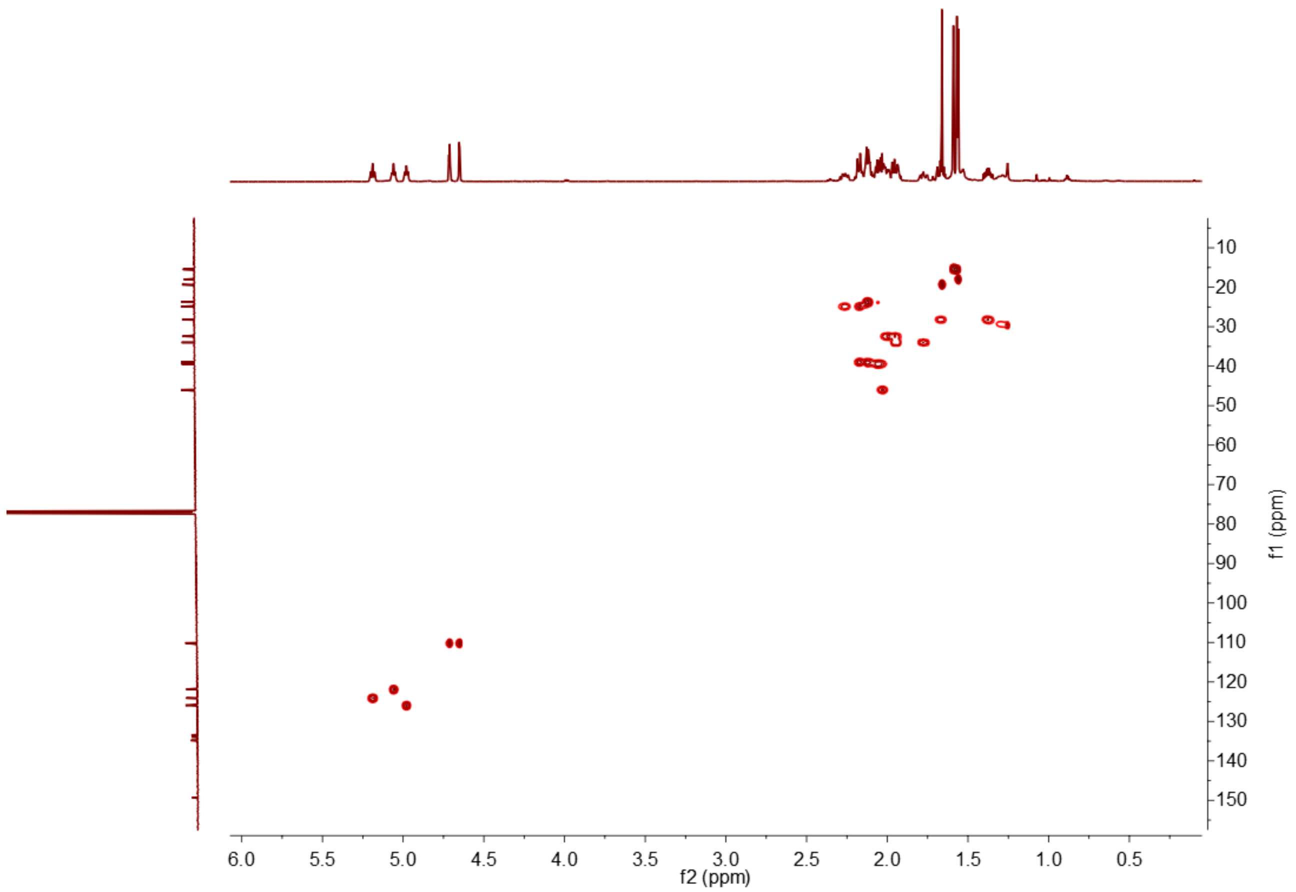

Supplementary Figure 31. The HSQC spectrum of **8** in $CDCl_3$.

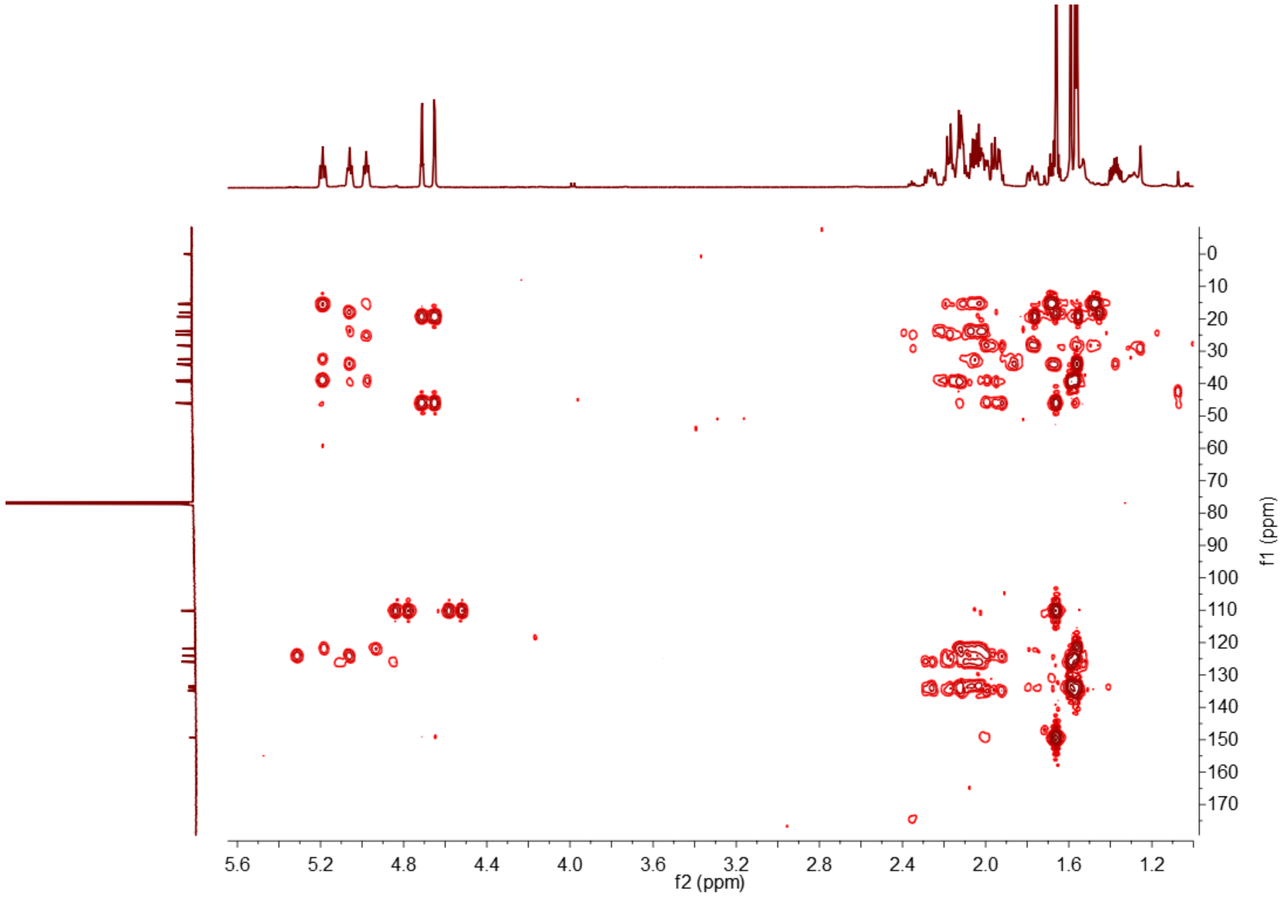

Supplementary Figure 32. The HMBC spectrum of **8** in $CDCl_3$.

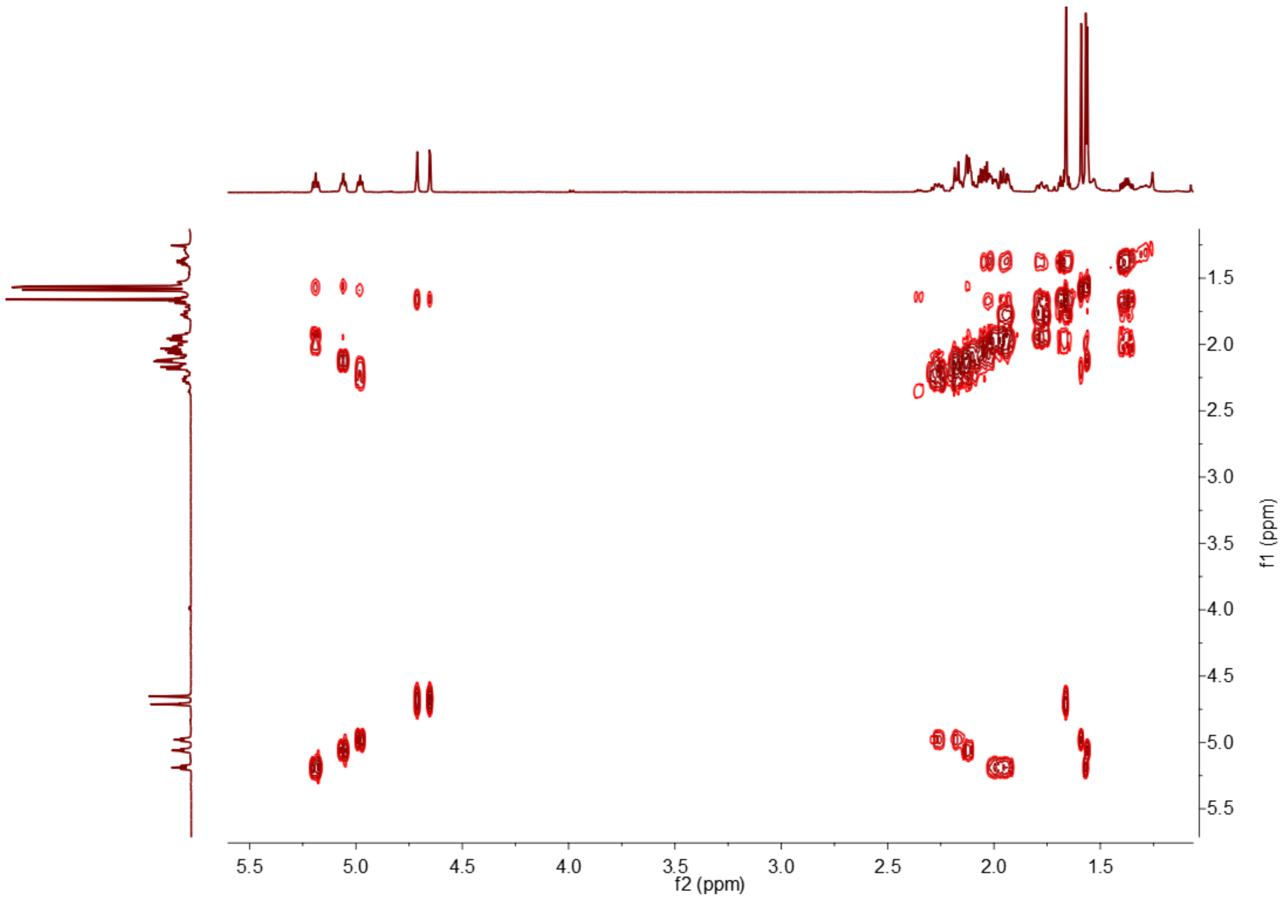

Supplementary Figure 33. The ^{1}H-^{1}H COSY spectrum of **8** in $CDCl_3$.

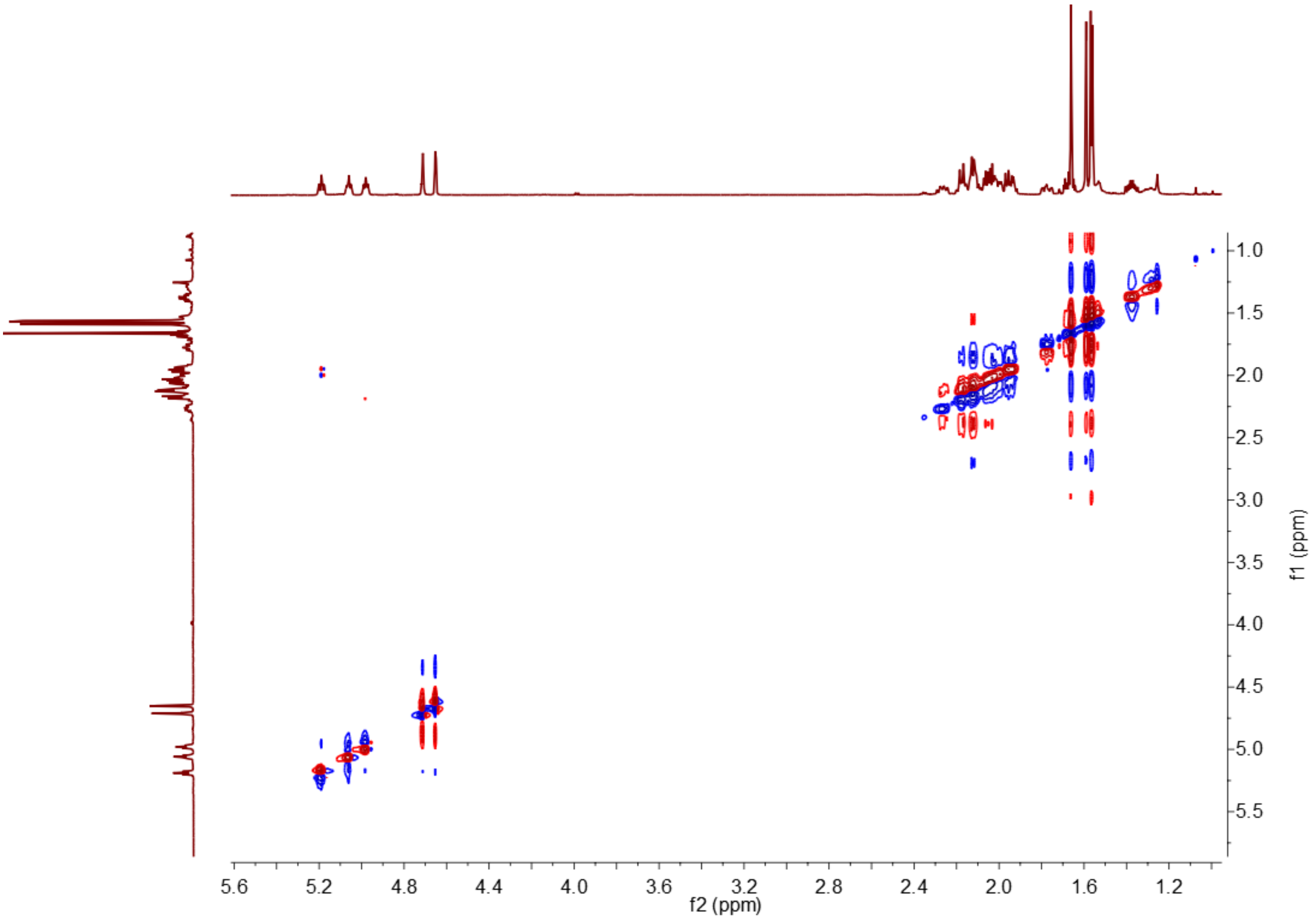

Supplementary Figure 34. The NOESY spectrum of **8** in $CDCl_3$.

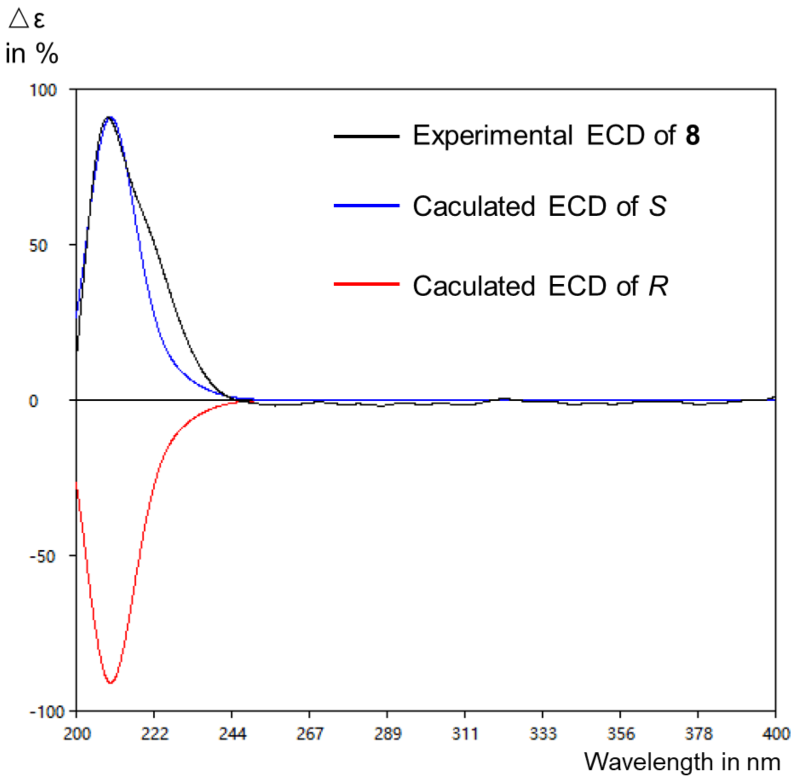

Supplementary Figure 35. The electronic circular dichroism (ECD) spectral analysis of **8** (*S*).

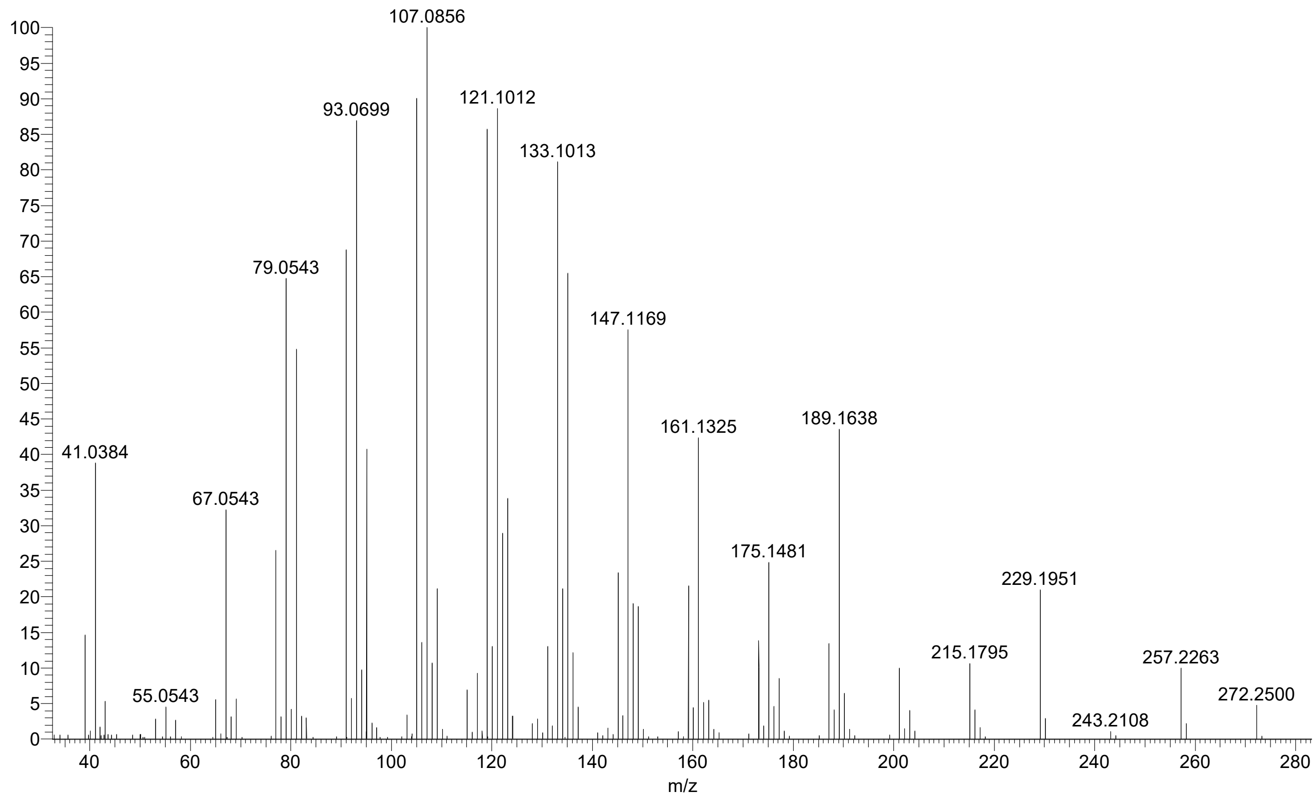

Supplementary Figure 36. The high-resolution GC mass spectrum of **9** (*calc.* 272.2499; *obs.* 272.2500).

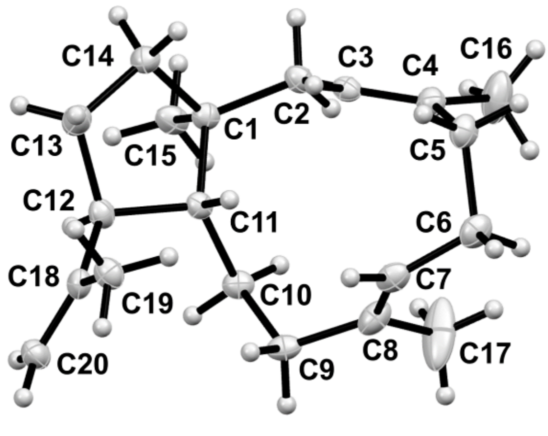

Supplementary Figure 37. ORTEP plot of the crystal structure of **9**.

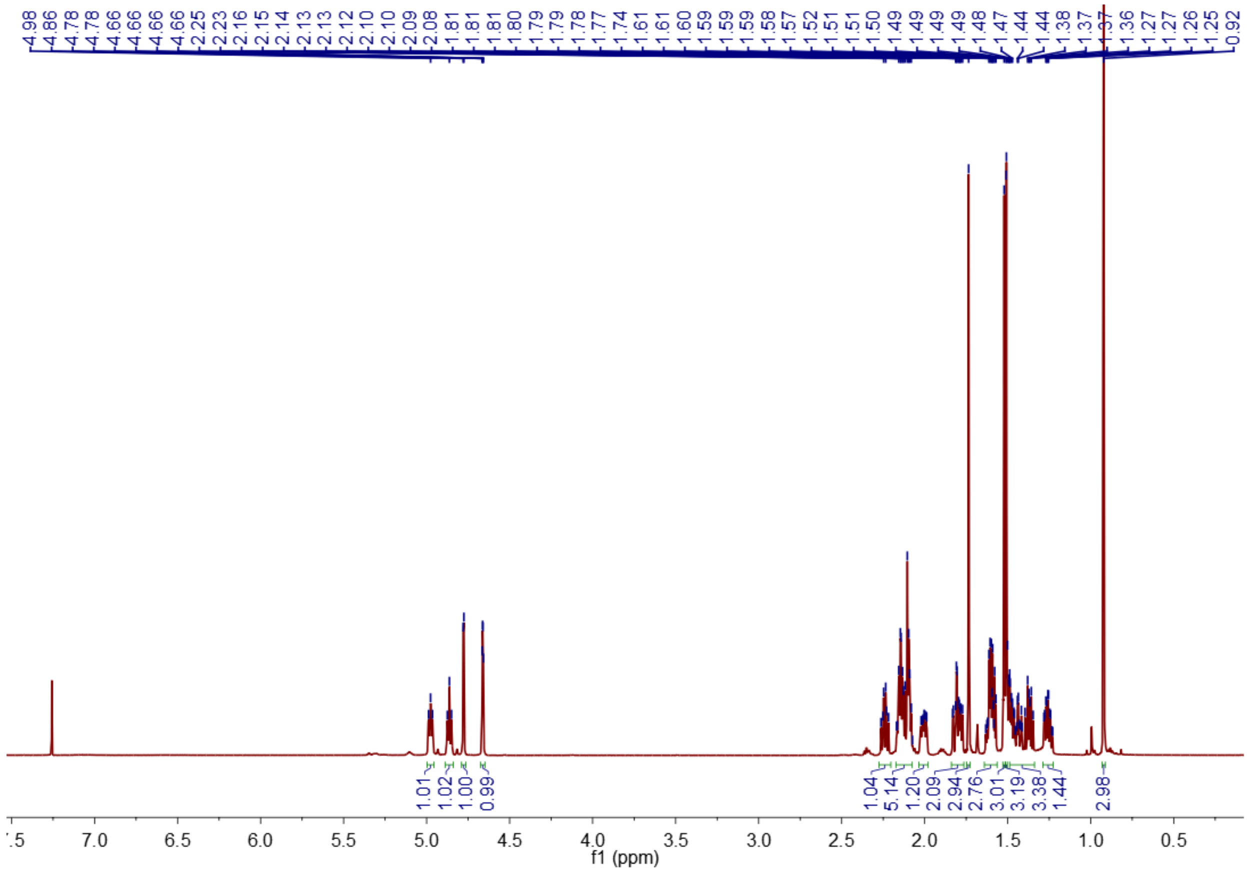

Supplementary Figure 38. The ^{1}H NMR spectrum of **9** in $CDCl_3$.

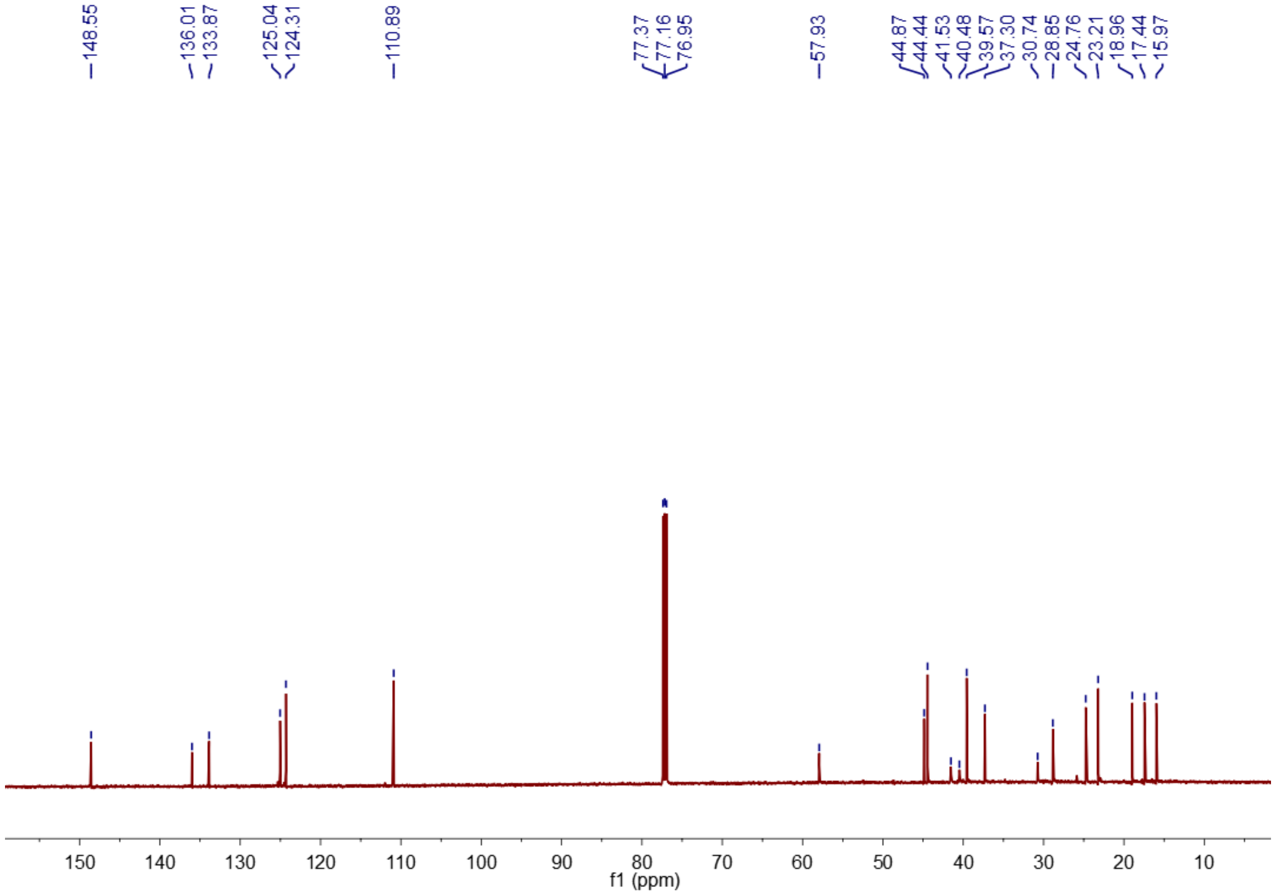

Supplementary Figure 39. The ^{13}C NMR spectrum of **9** in $CDCl_3$.

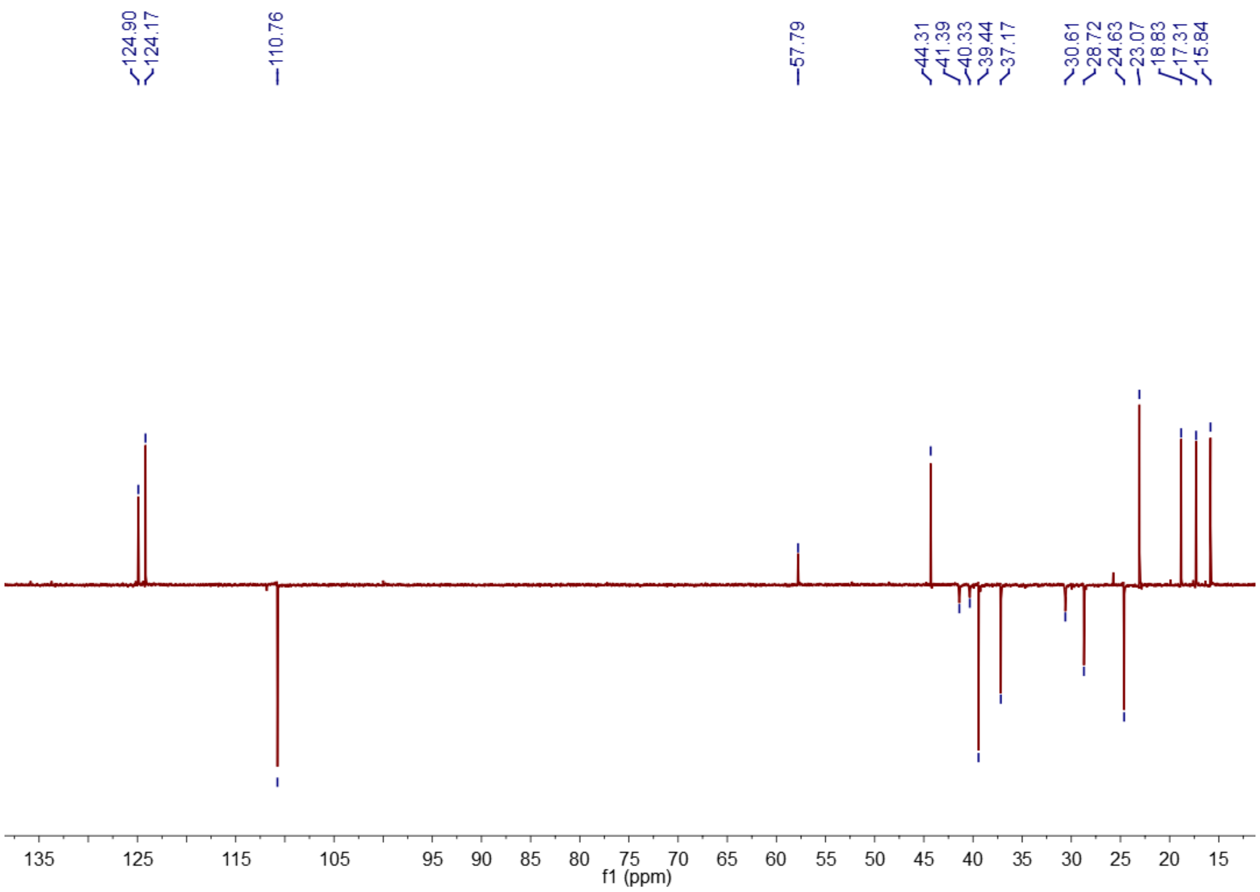

Supplementary Figure 40. The ^{13}C-DEPT 135° spectrum of **9** in $CDCl_3$.

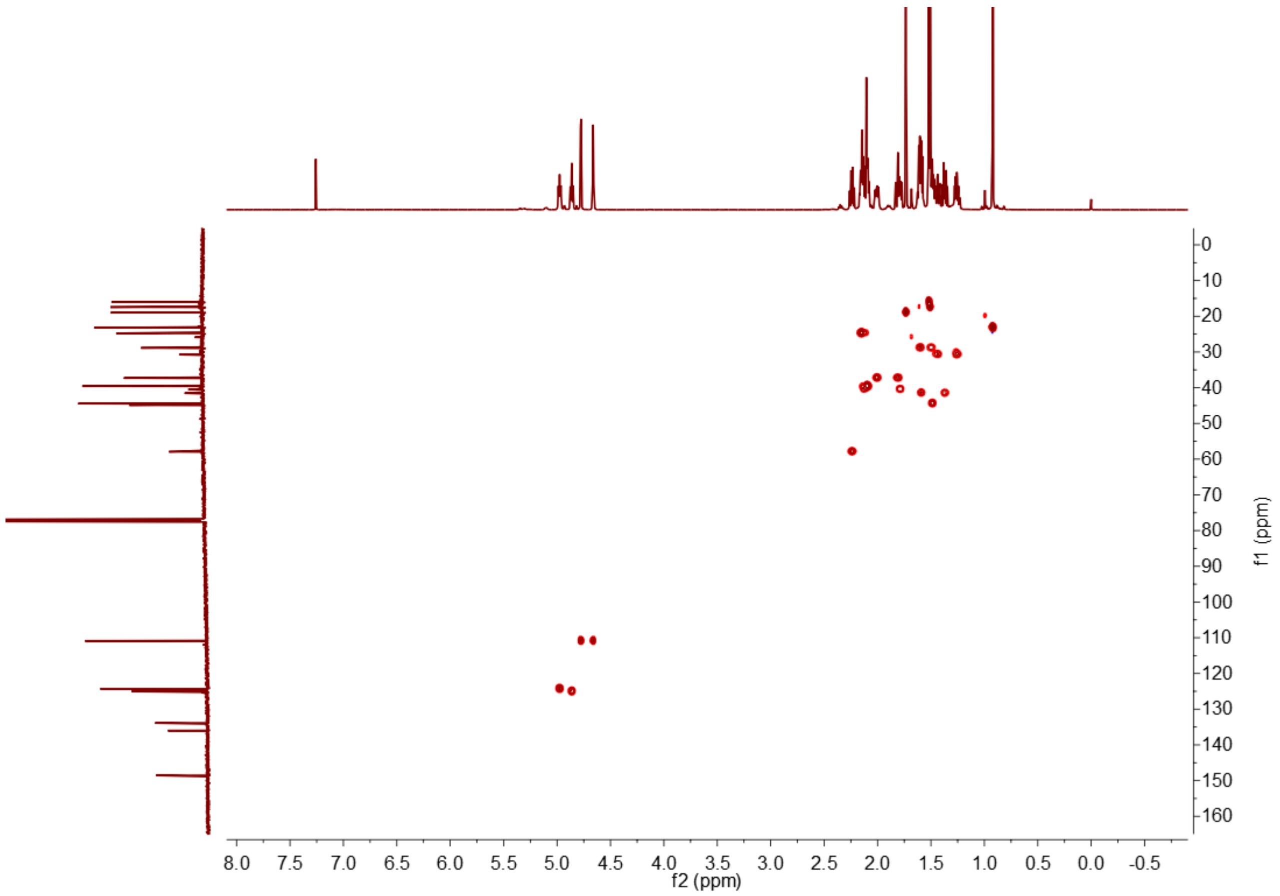

Supplementary Figure 41. The HSQC spectrum of **9** in $CDCl_3$.

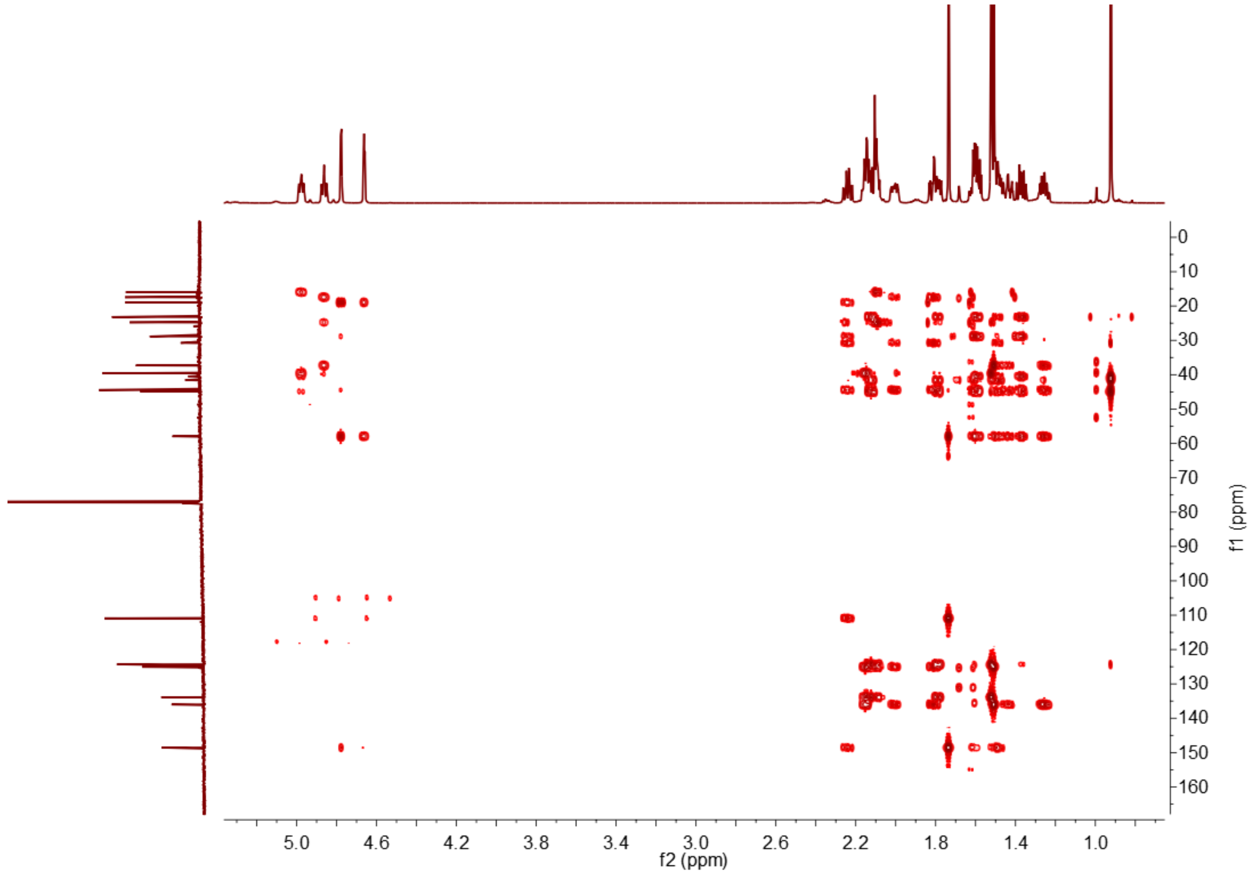

Supplementary Figure 42. The HMBC spectrum of **9** in $CDCl_3$.

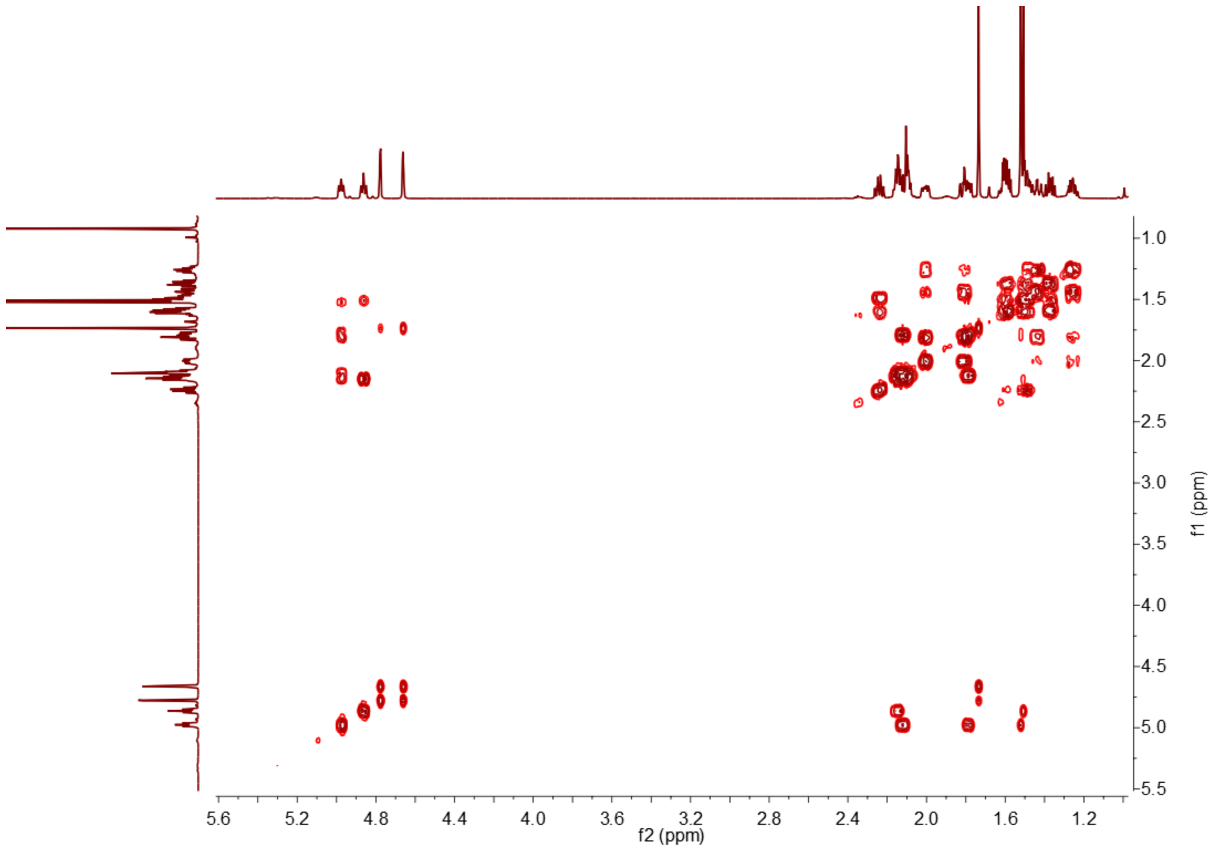

Supplementary Figure 43. The ^1H-^1H COSY spectrum of **9** in $CDCl_3$.

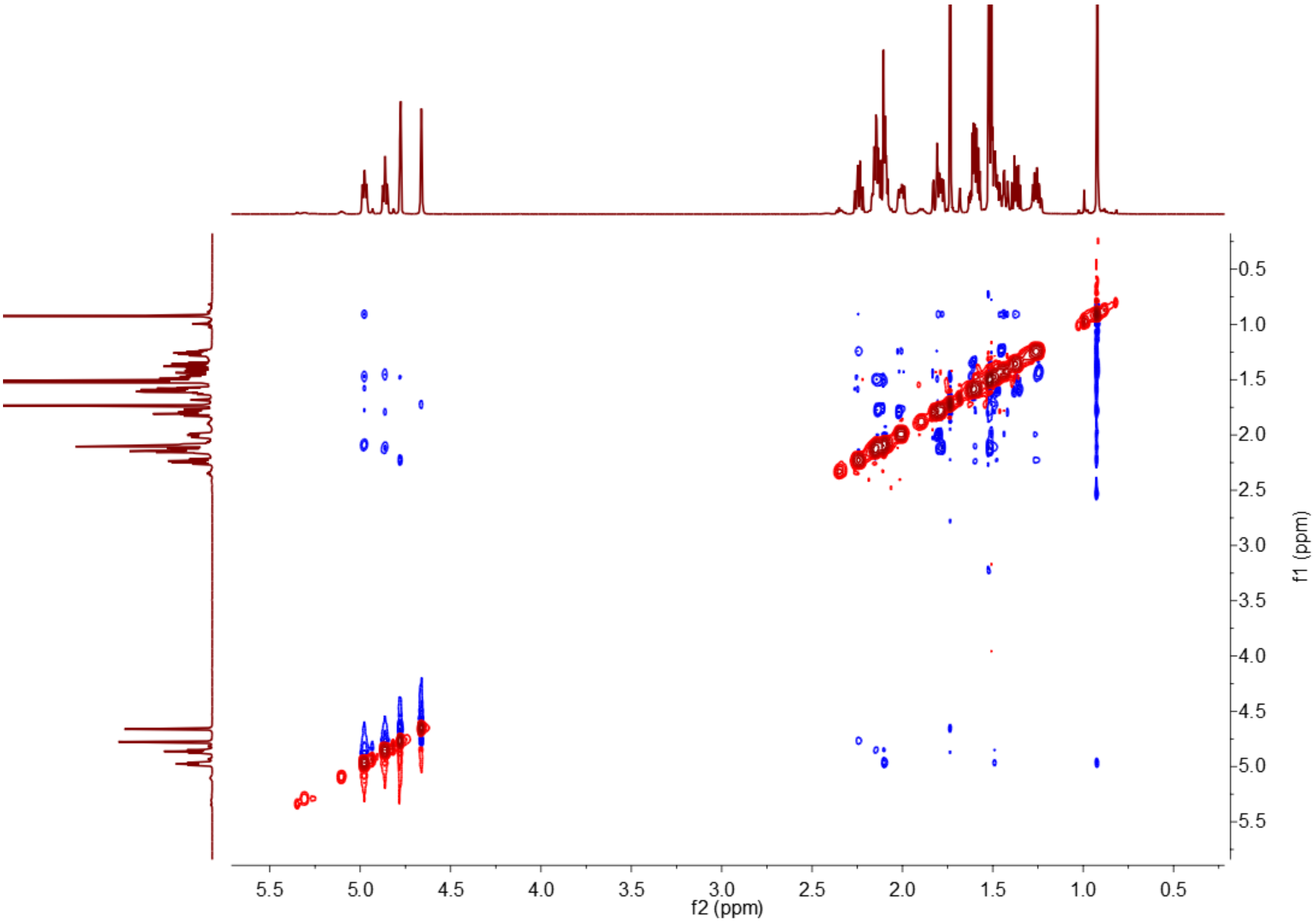

Supplementary Figure 44. The NOESY spectrum of **9** in $CDCl_3$.

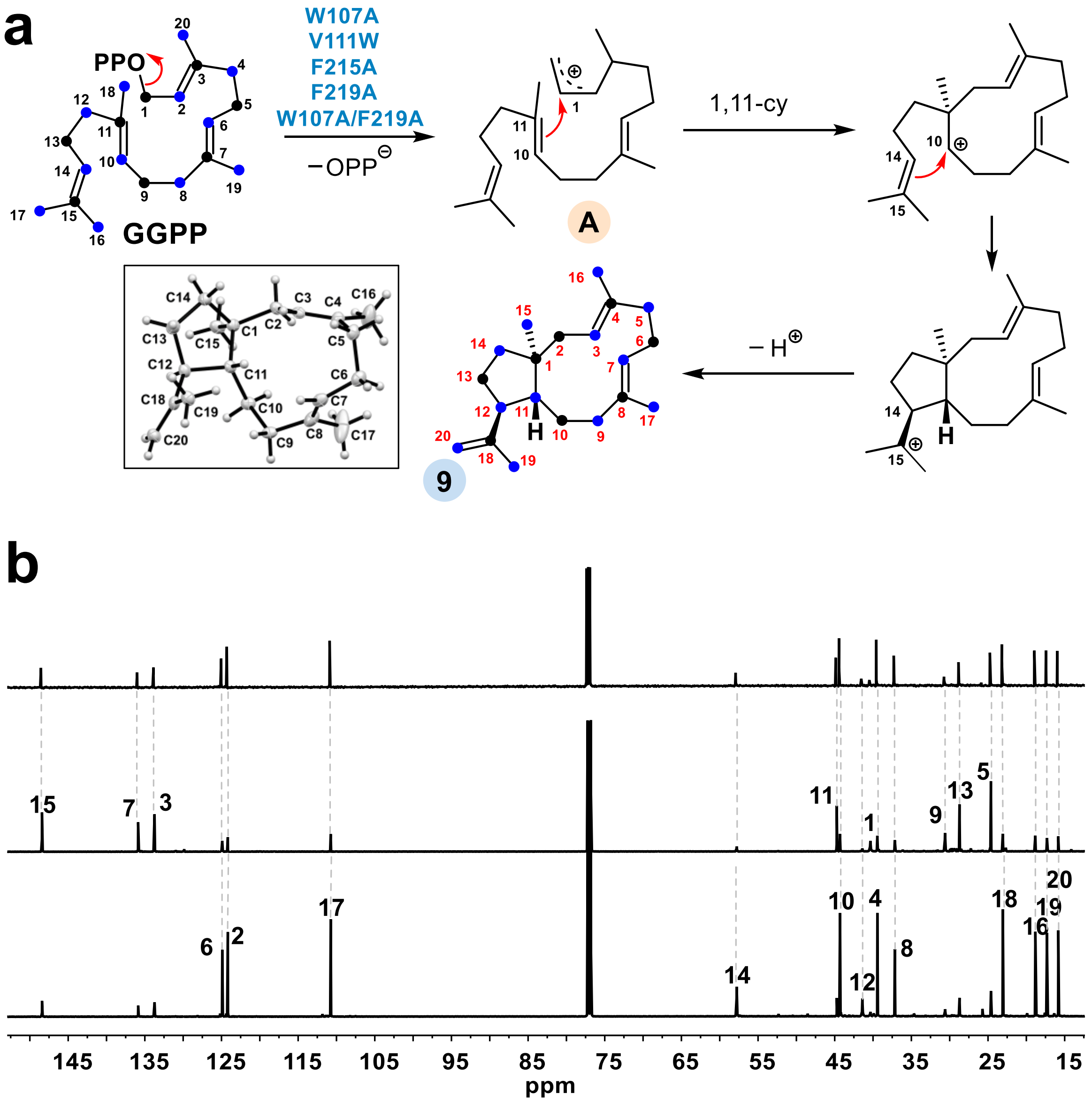

Supplementary Figure 45. The use of ^{13}C-labelled precursors for tracing **9** biosynthesis. **a**, The proposed cyclization mechanism of GGPP to form **9**. **b**, ^{13}C NMR spectra of unlabelled and differentially ^{13}C labelled **9**. (***top***) The ^{13}C NMR spectrum of unlabelled **9**; The ^{13}C NMR spectrum of the ^{13}C-labelled **9** obtained from the feeding of (1-^{13}C)-labelled sodium (***middle***) and (2-^{13}C)-labelled sodium acetate (***bottom***) to the recombinant *E. coli* strain of Eco-A^{W107A}D. The numbering of carbons indicates their origin from GGPP and derives from the numbering system used for **9** (makered by red color) [16].

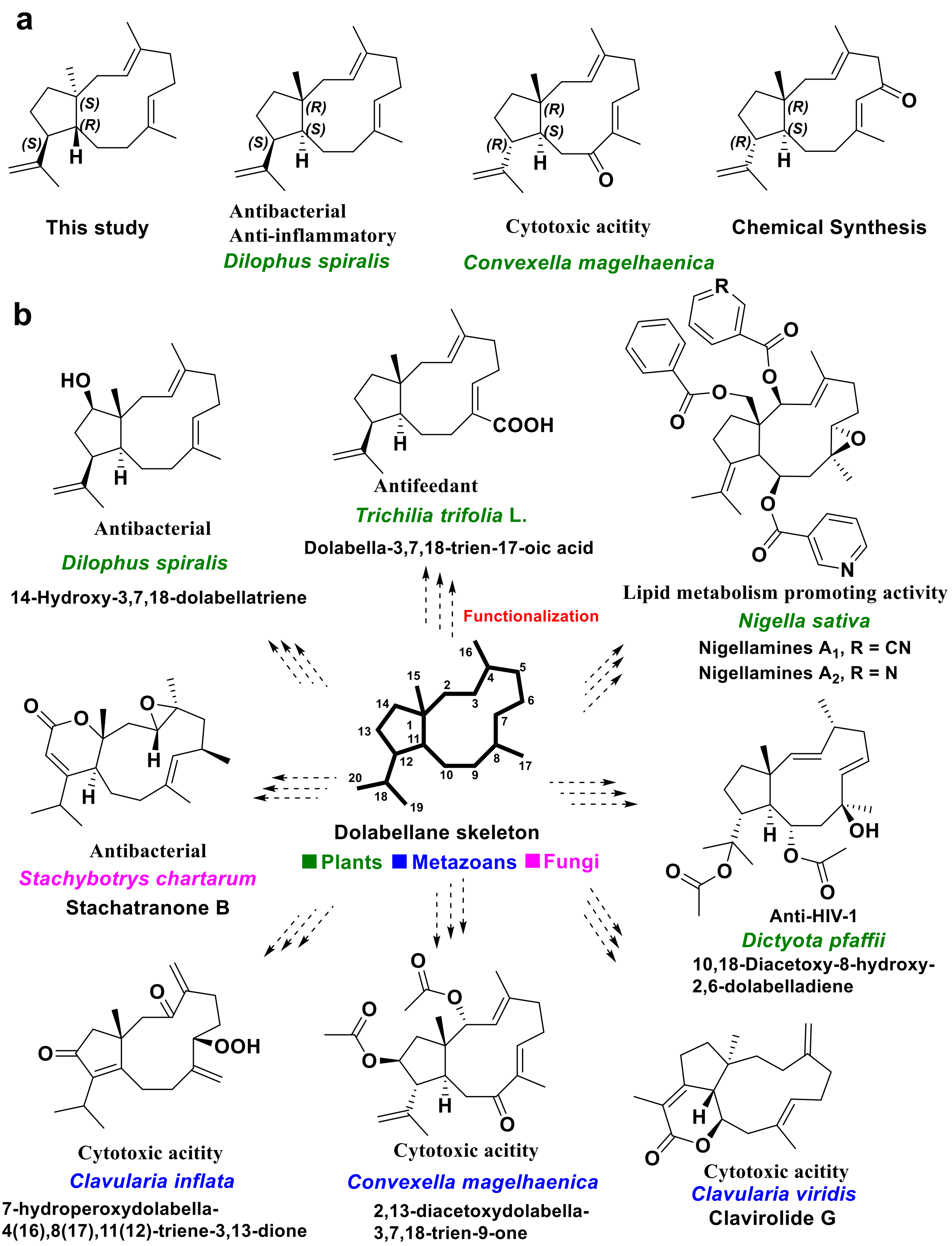

Supplementary Figure 46. The known conformations of 3,7,18-dolabellatriene and its derivatives (**a**) and the selected bioactive dolabellane-type diterpenoids (**b**). The carbon atoms are numbered according to the previous literature[16].

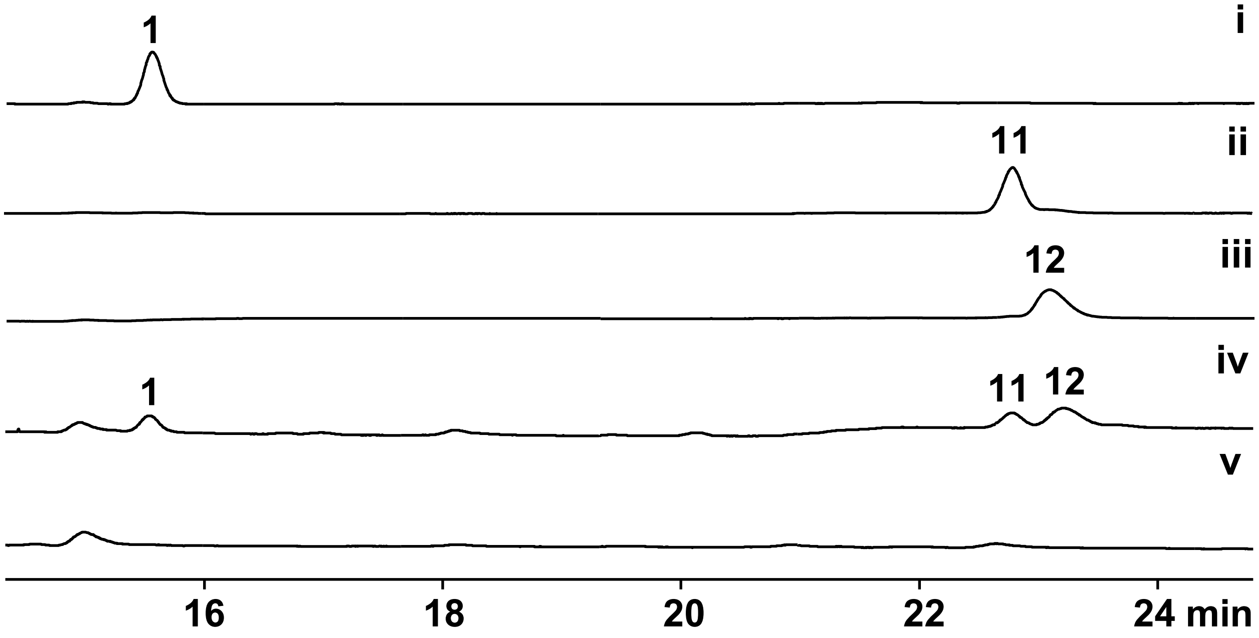

Supplementary Figure 47. GC analysis of the reaction of VenA^{F185A} toward GGPP. (i) Authentic standard of **1**; (ii) Authentic standard of **11**; (iii) Authentic standard of **12**; (iv) VenA^{F185A} reaction; (v) The control reaction of (iv).

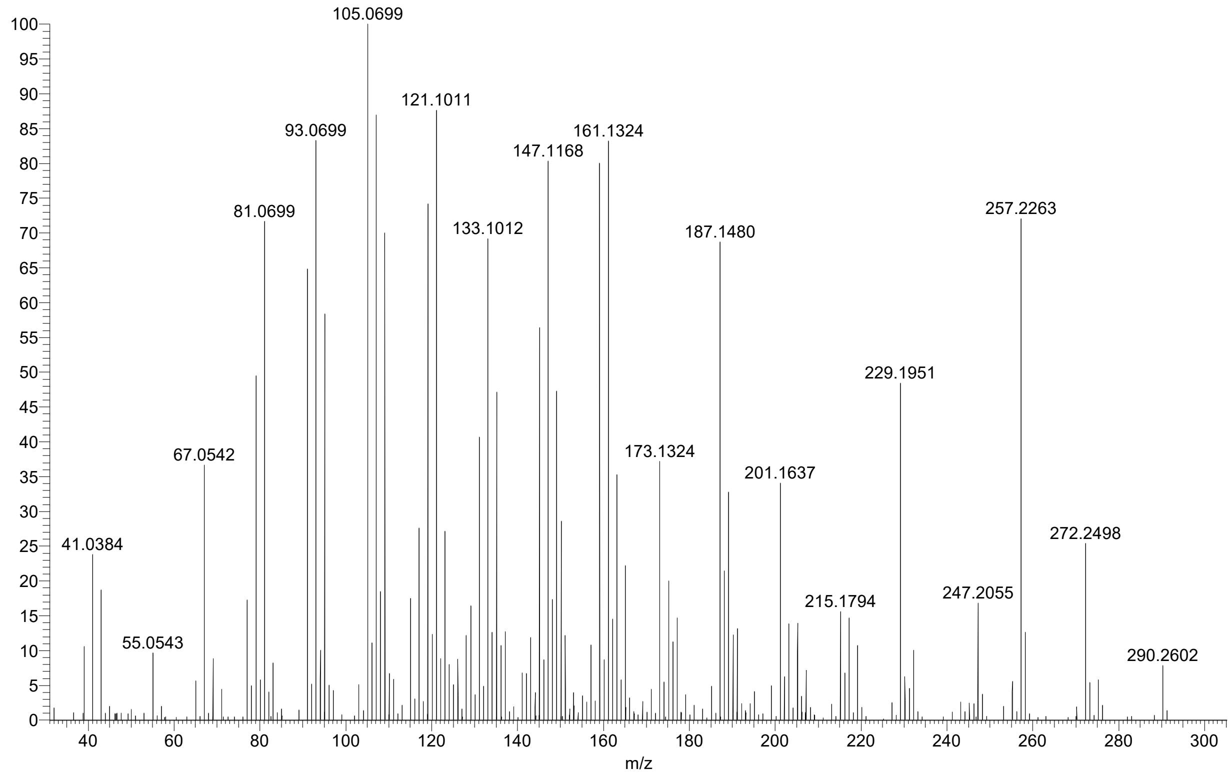

Supplementary Figure 48. The high-resolution GC mass spectrum of **11** (*calc.* 290.2604; *obs.* 290.2602).

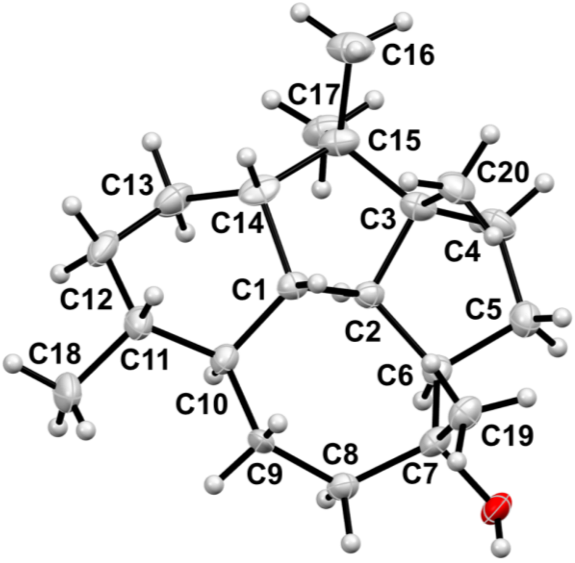

Supplementary Figure 49. ORTEP plot of the crystal structure of **11**.

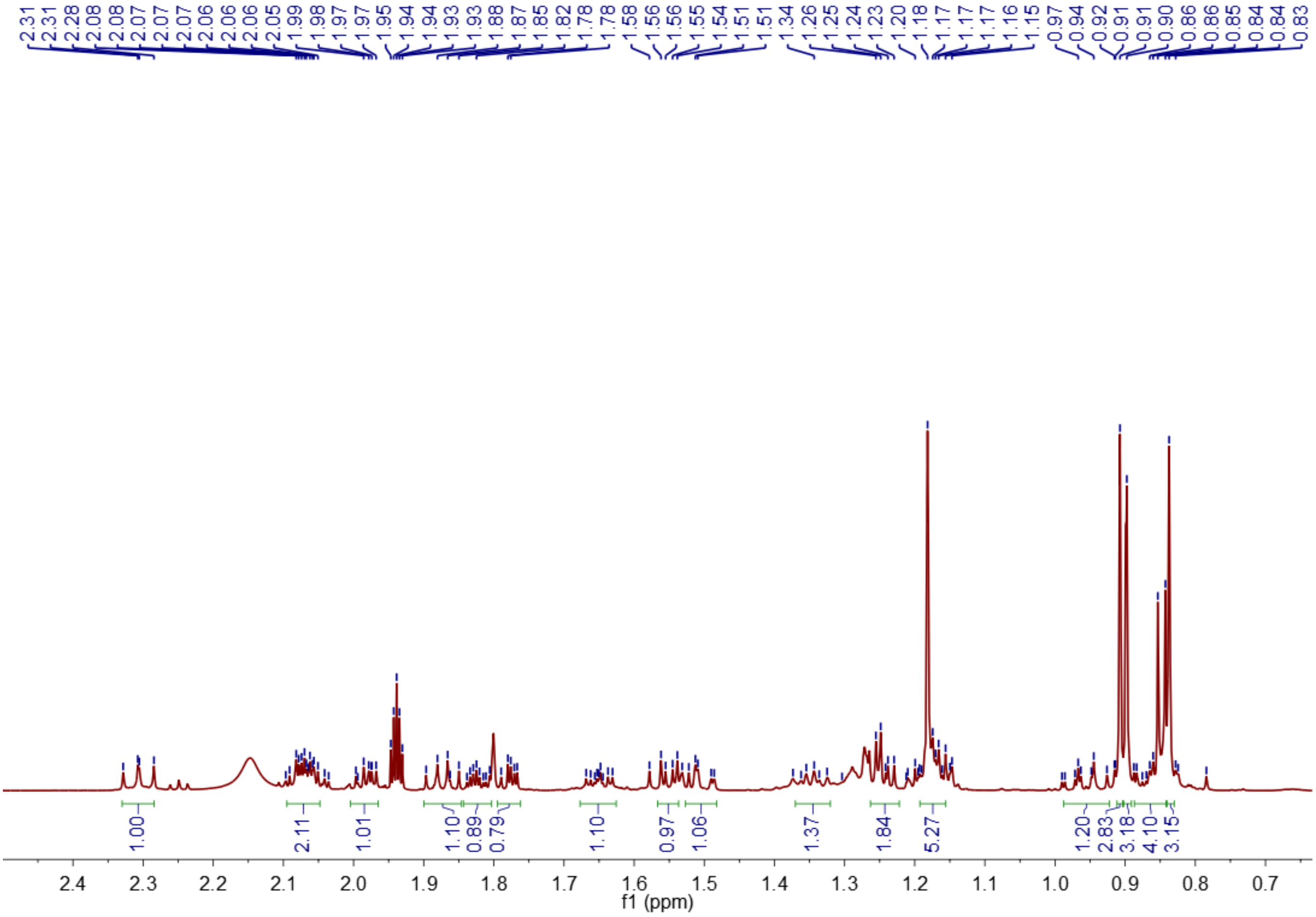

Supplementary Figure 50. The ^{1}H NMR spectrum of **11** in CD_3CN.

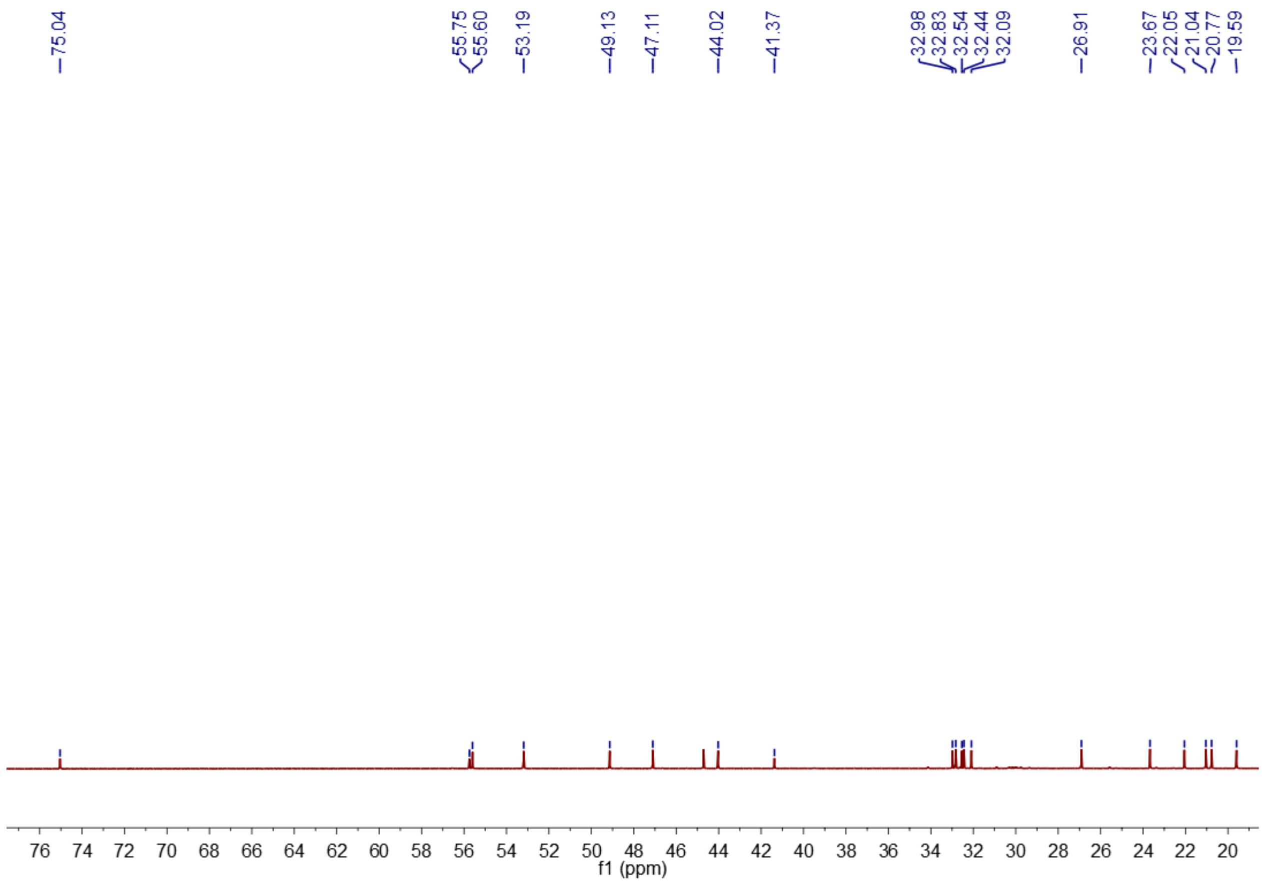

Supplementary Figure 51. The ^{13}C NMR spectrum of **11** in CD_3CN.

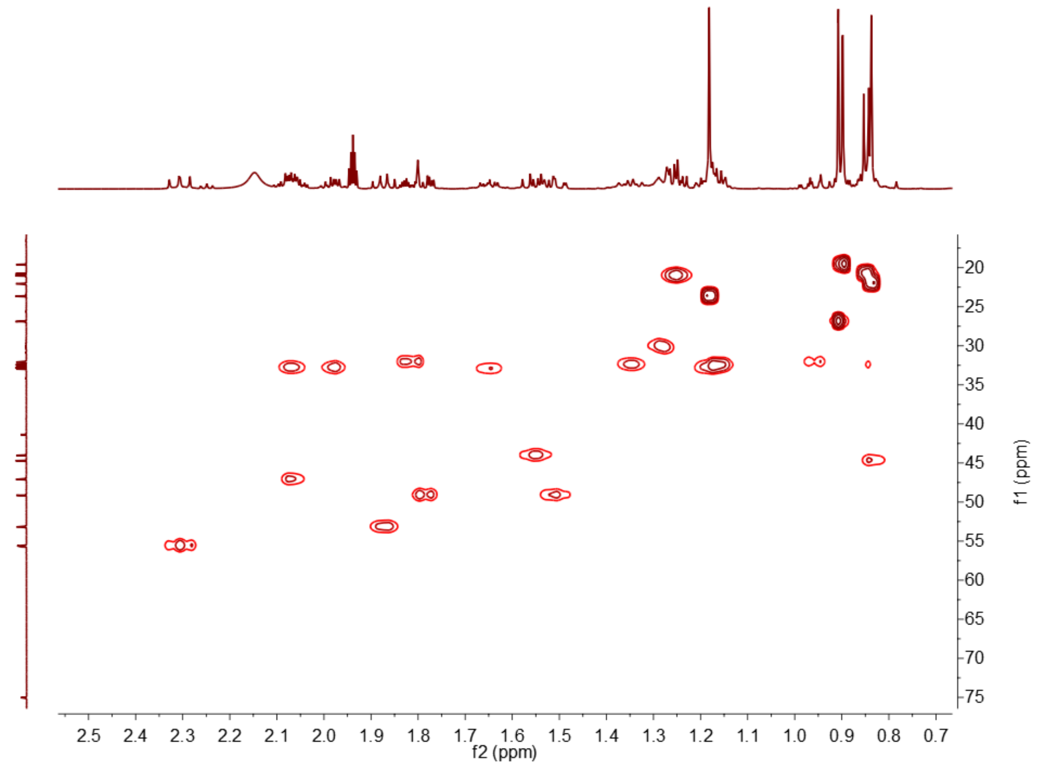

Supplementary Figure 52. The HSQC spectrum of **11** in CD_3CN.

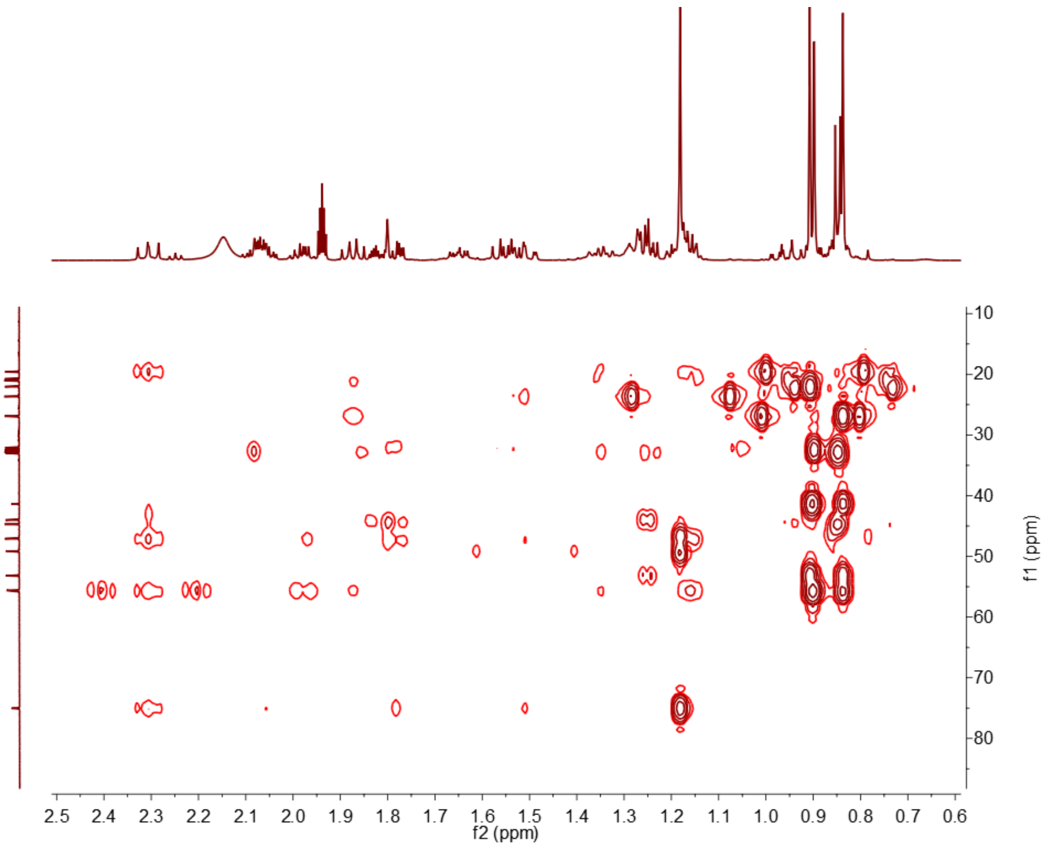

Supplementary Figure 53. The HMBC spectrum of **11** in CD3CN.

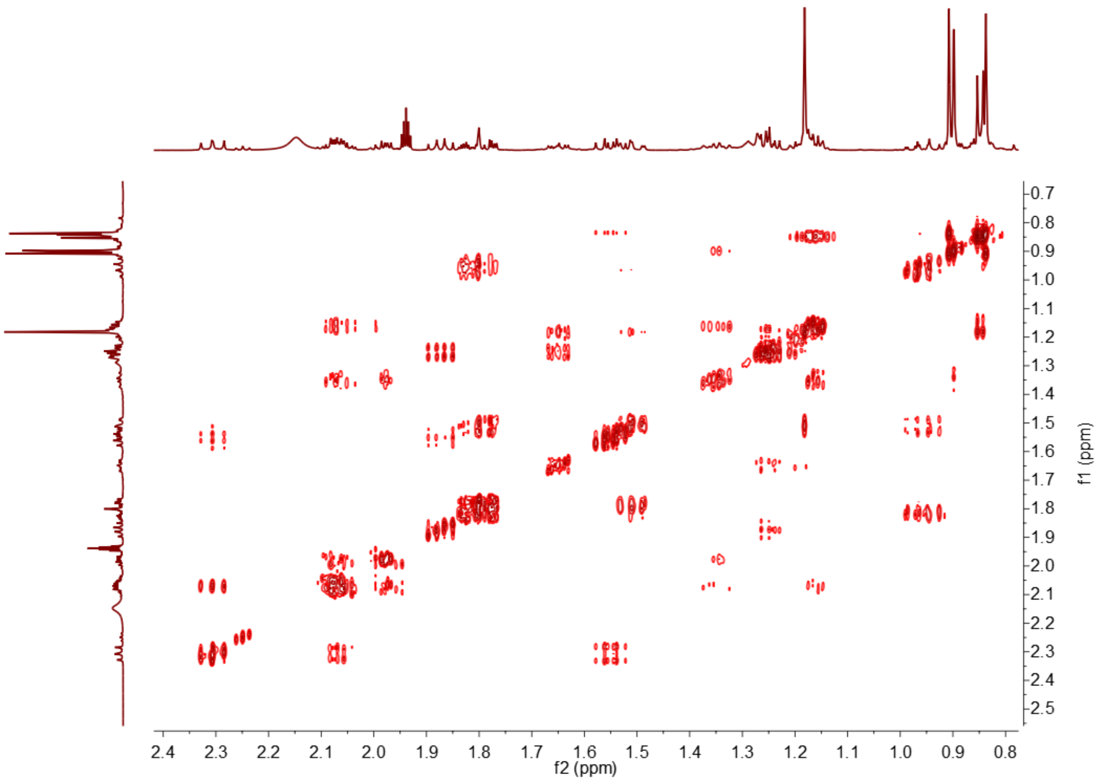

Supplementary Figure 54. The ^{1}H-^{1}H COSY spectrum of **11** in CD3CN.

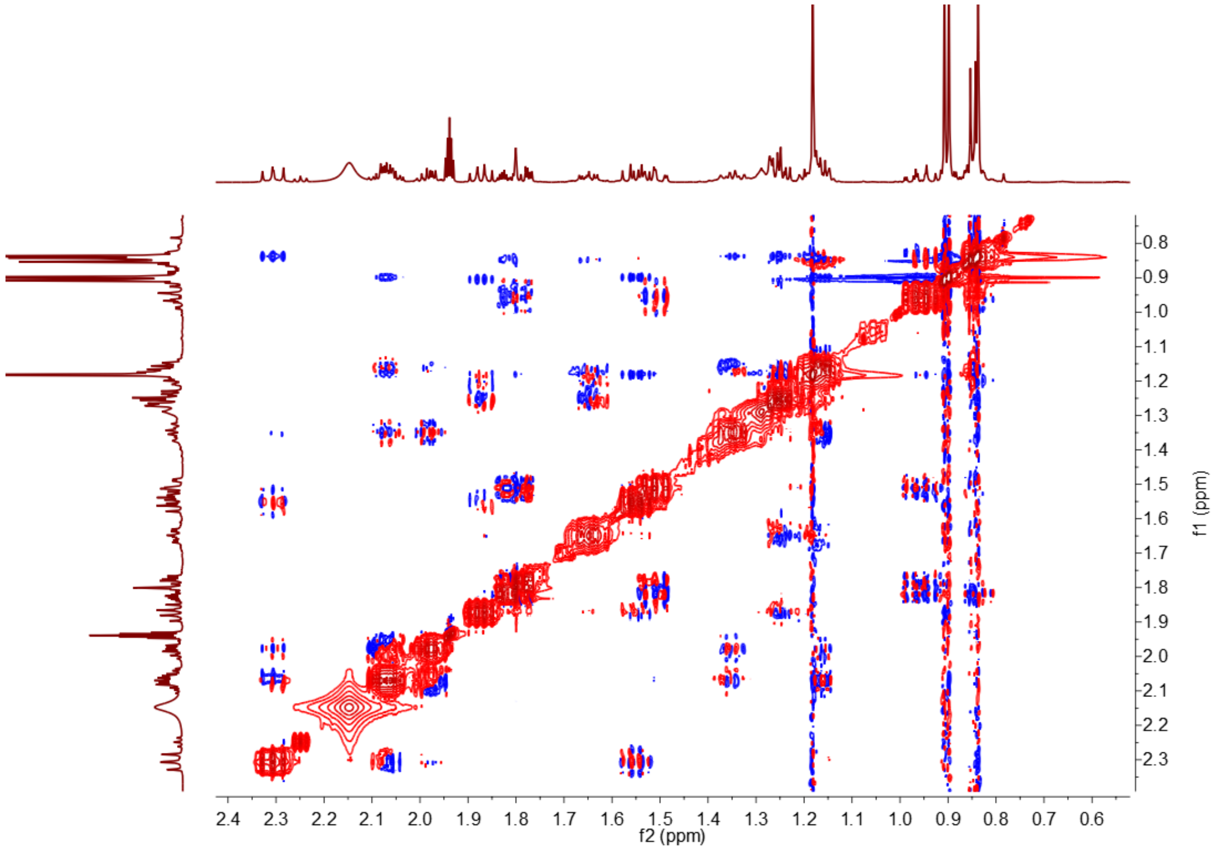

Supplementary Figure 55. The NOESY spectrum of **11** in CD_3CN.

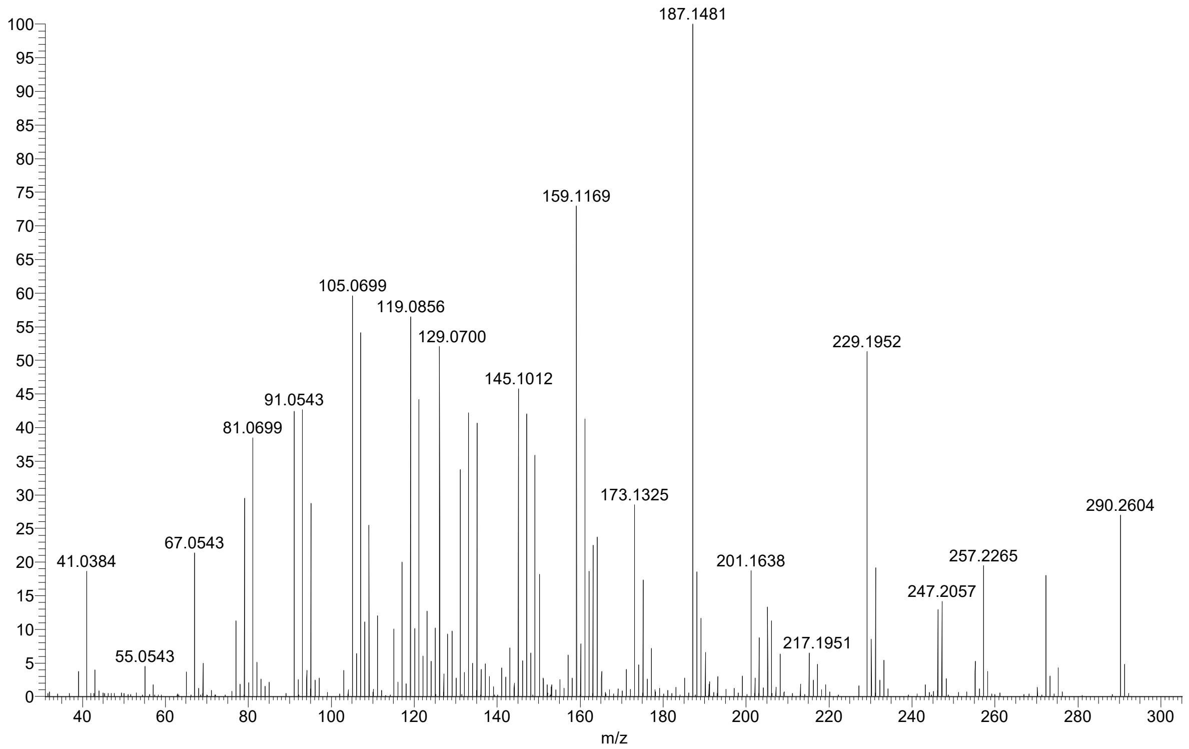

Supplementary Figure 56. The high-resolution GC mass spectrum of **12** (*calc.* 290.2604; *obs.* 290.2604).

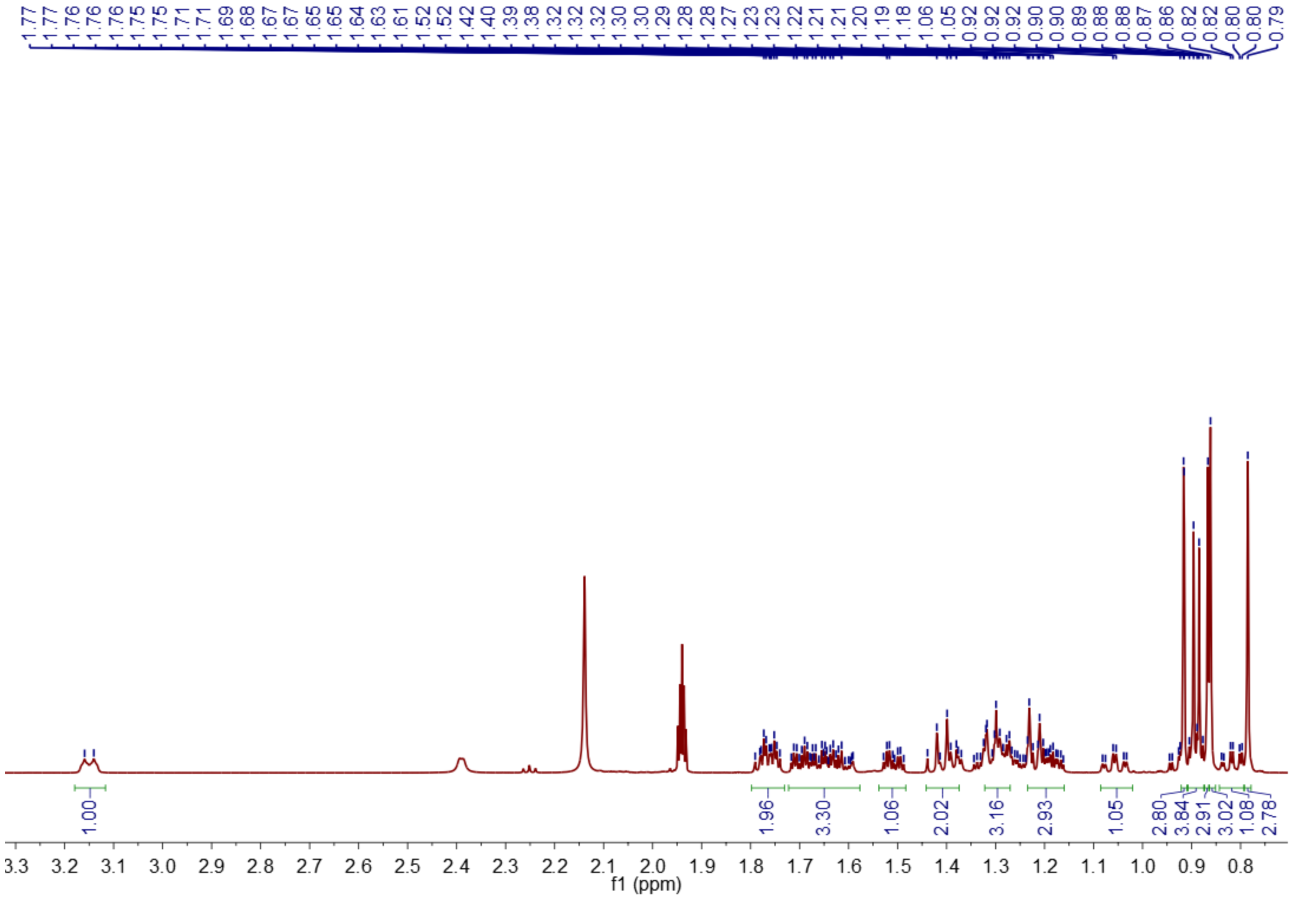

Supplementary Figure 57. The ^{1}H NMR spectrum of **12** in CD_3CN.

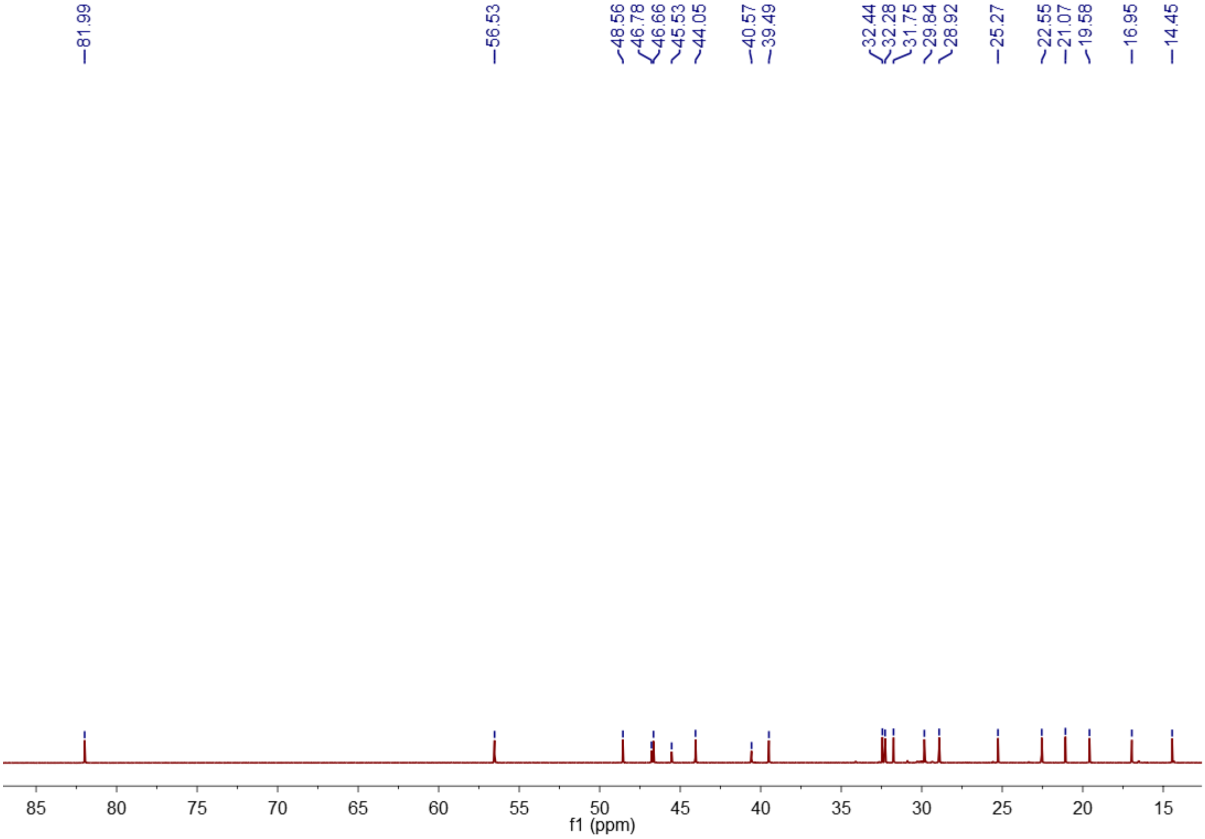

Supplementary Figure 58. The ^{13}C NMR spectrum of **12** in CD_3CN.

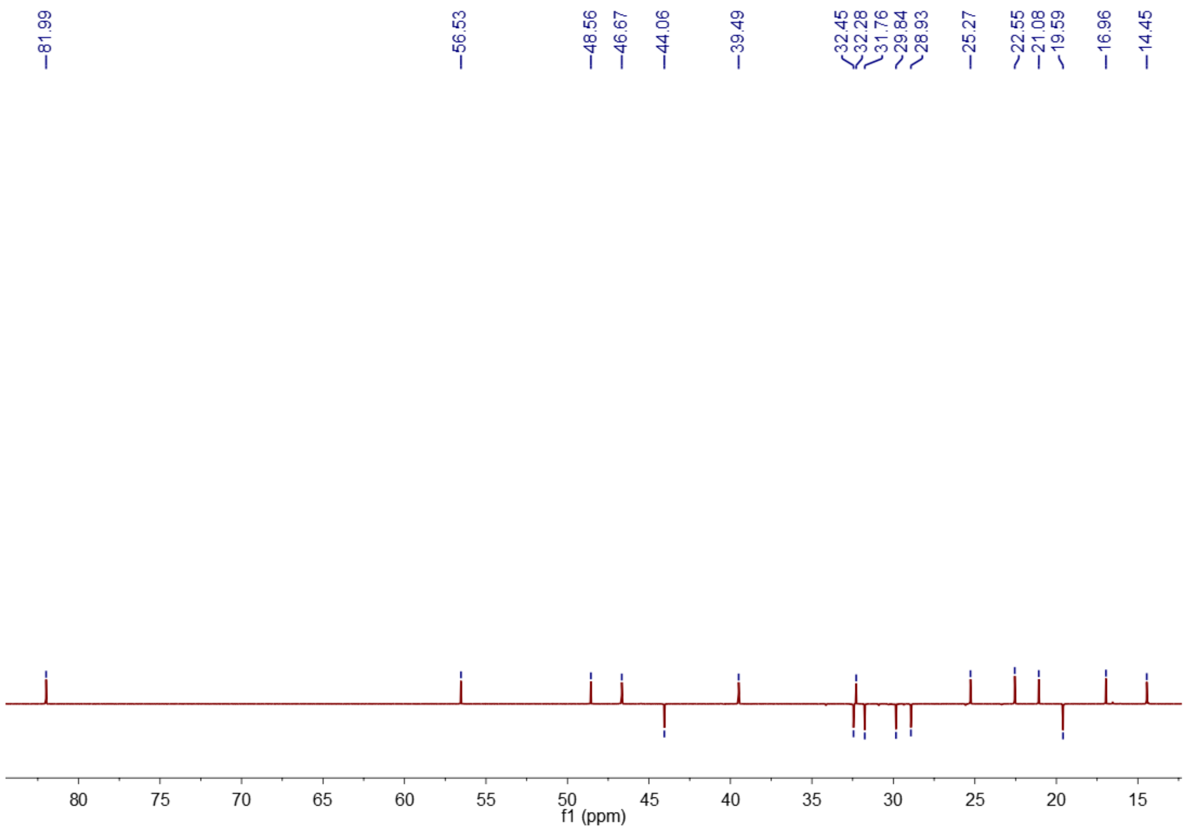

Supplementary Figure 59. The ^{13}C-DEPT 135° spectrum of **12** in CD_3CN

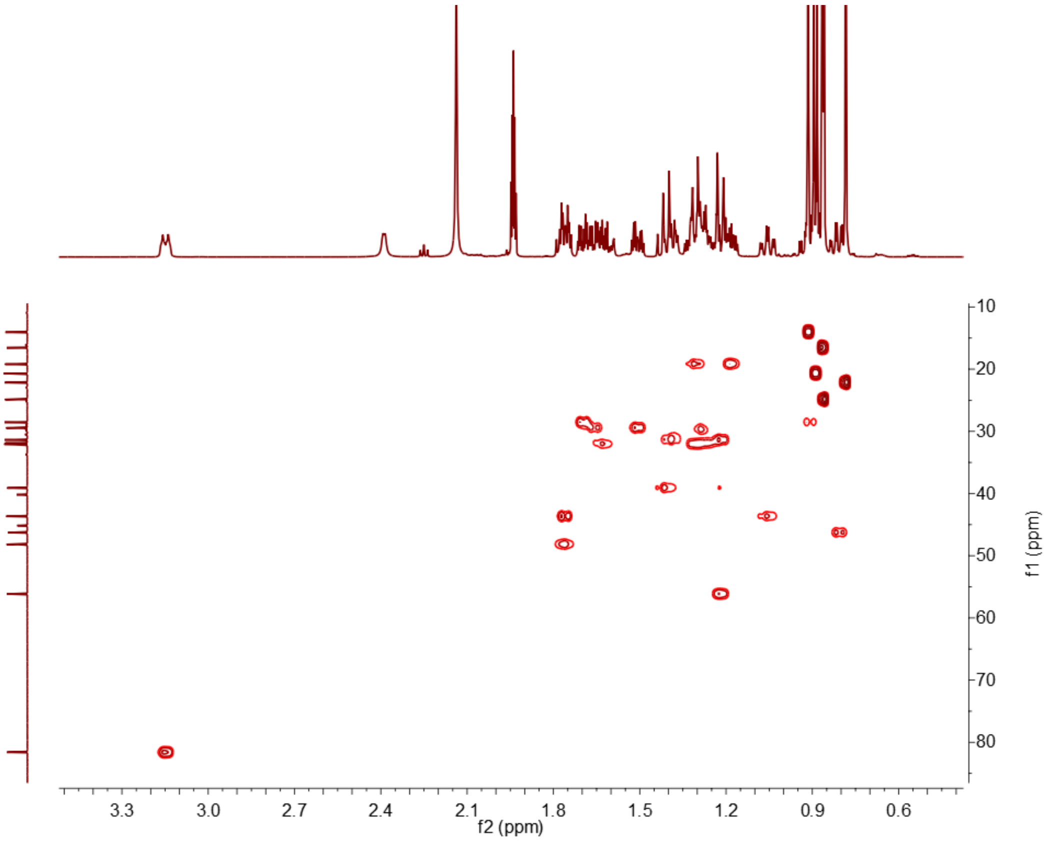

Supplementary Figure 60. The HSQC spectrum of **12** in CD_3CN.

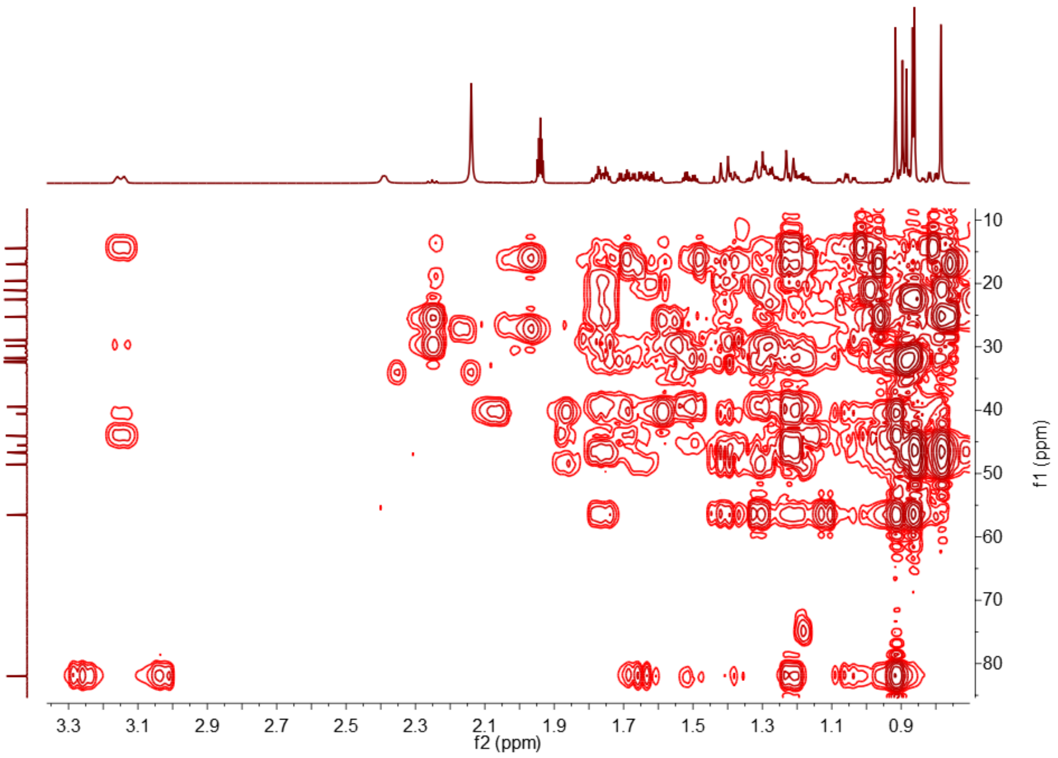

Supplementary Figure 61. The HMBC spectrum of **12** in CD_3CN.

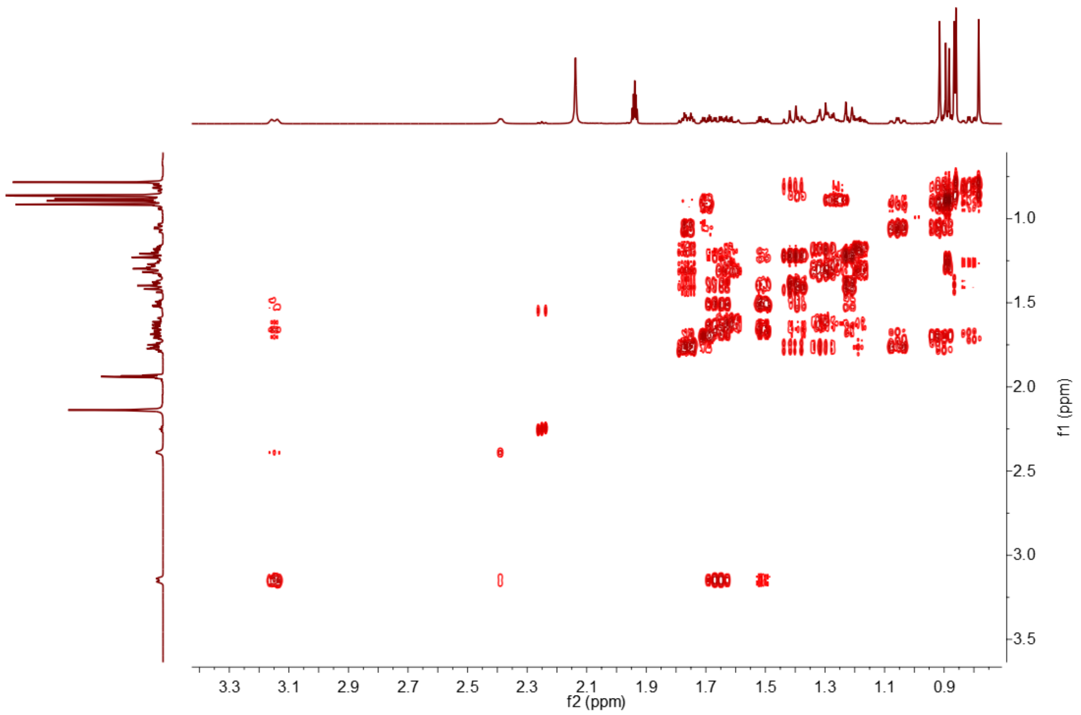

Supplementary Figure 62. The ^{1}H-^{1}H COSY spectrum of **12** in CD_3CN.

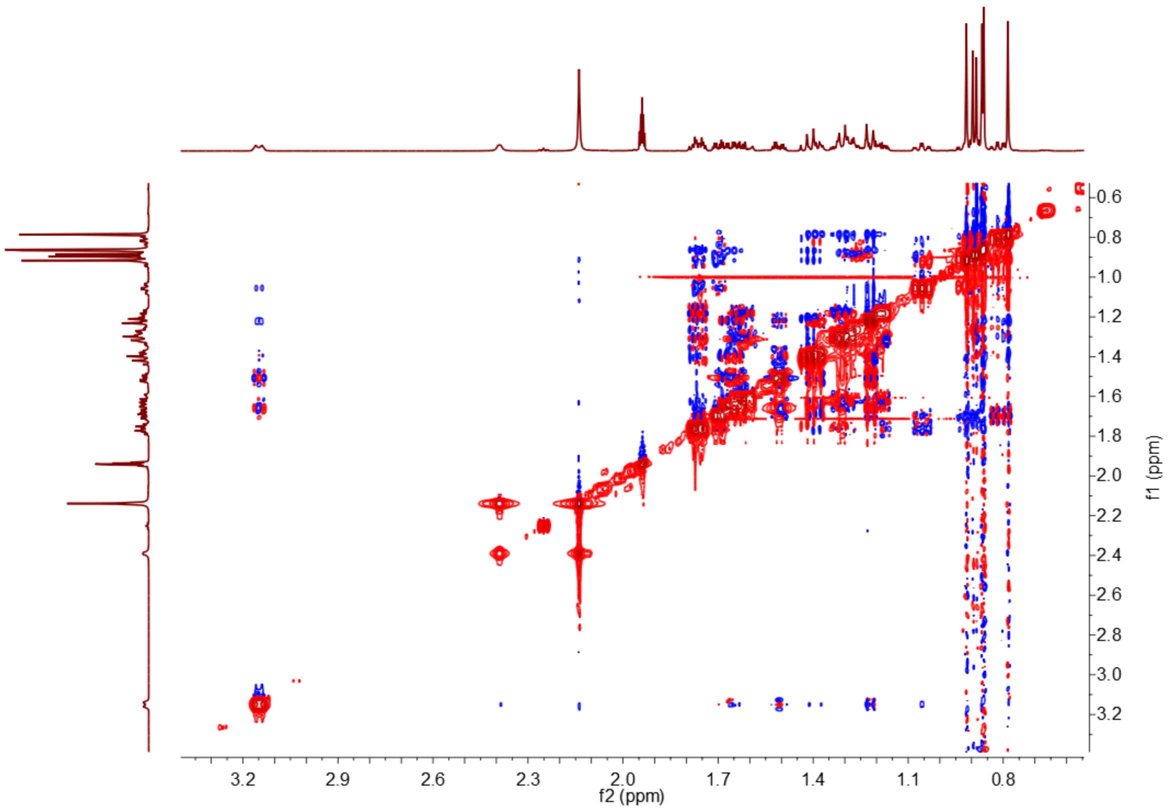

Supplementary Figure 63. The NOESY spectrum of **12** in CD_3CN.

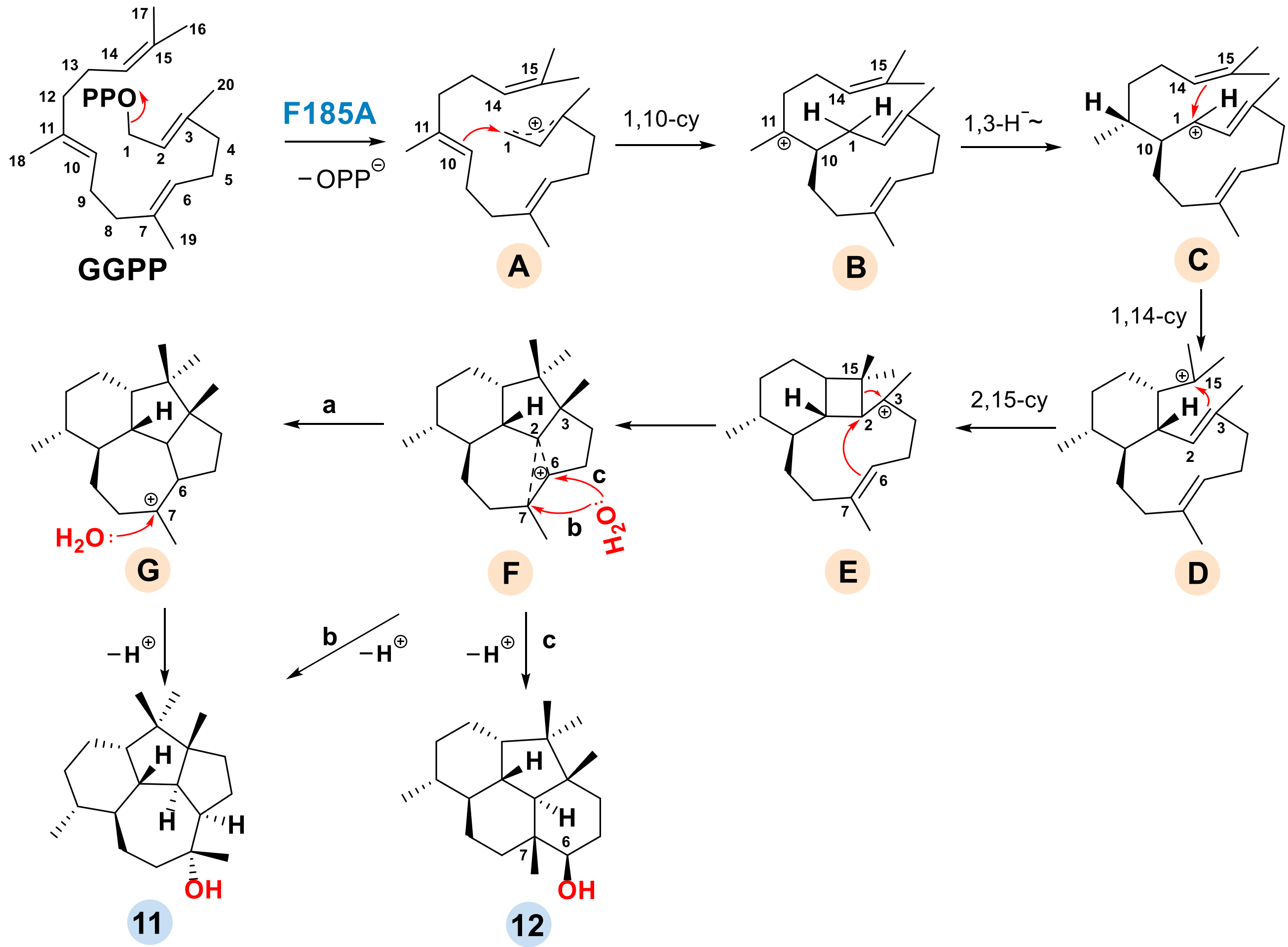

Supplementary Figure 64. Proposed biosynthetic mechanisms of **11** (**a** or **b**) and **12** (**c**) catalyzed by VenA^{F185A}.

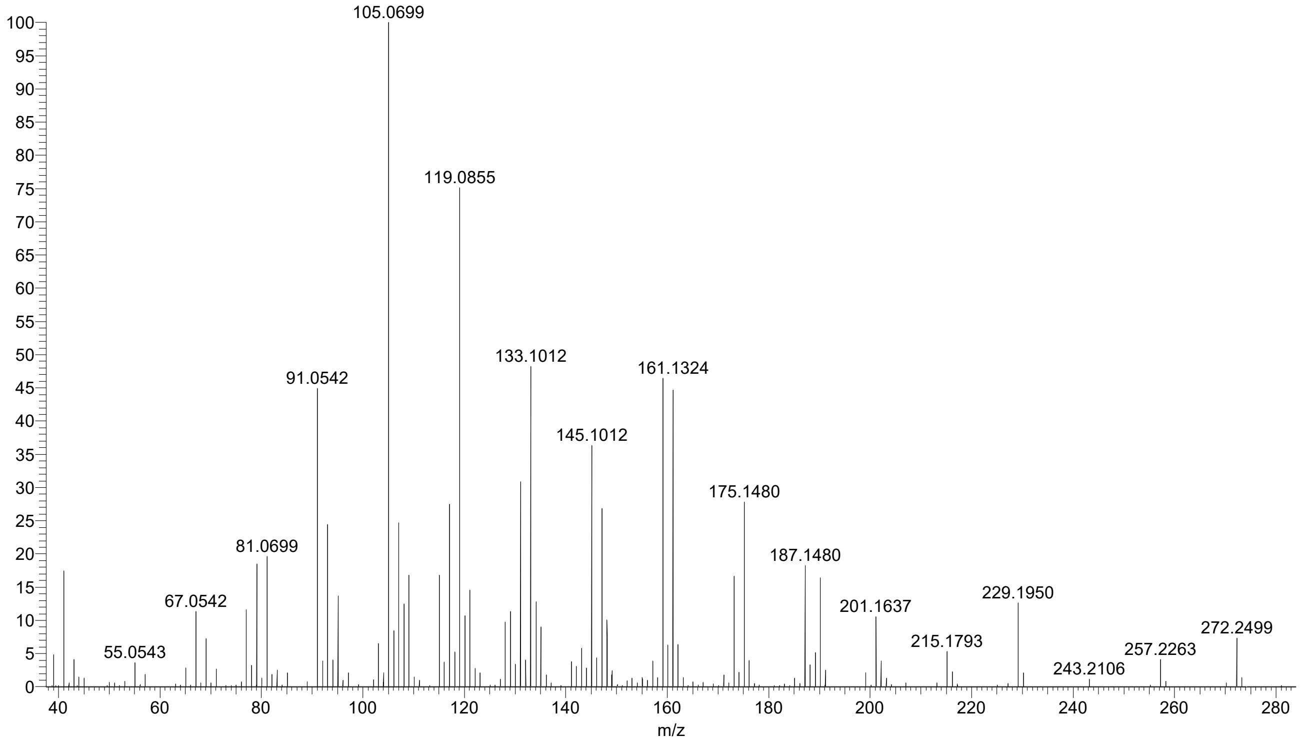

Supplementary Figure 65. The high-resolution GC mass spectrum of **10** (*calc.* 272.2499; *obs.* 272.2499).

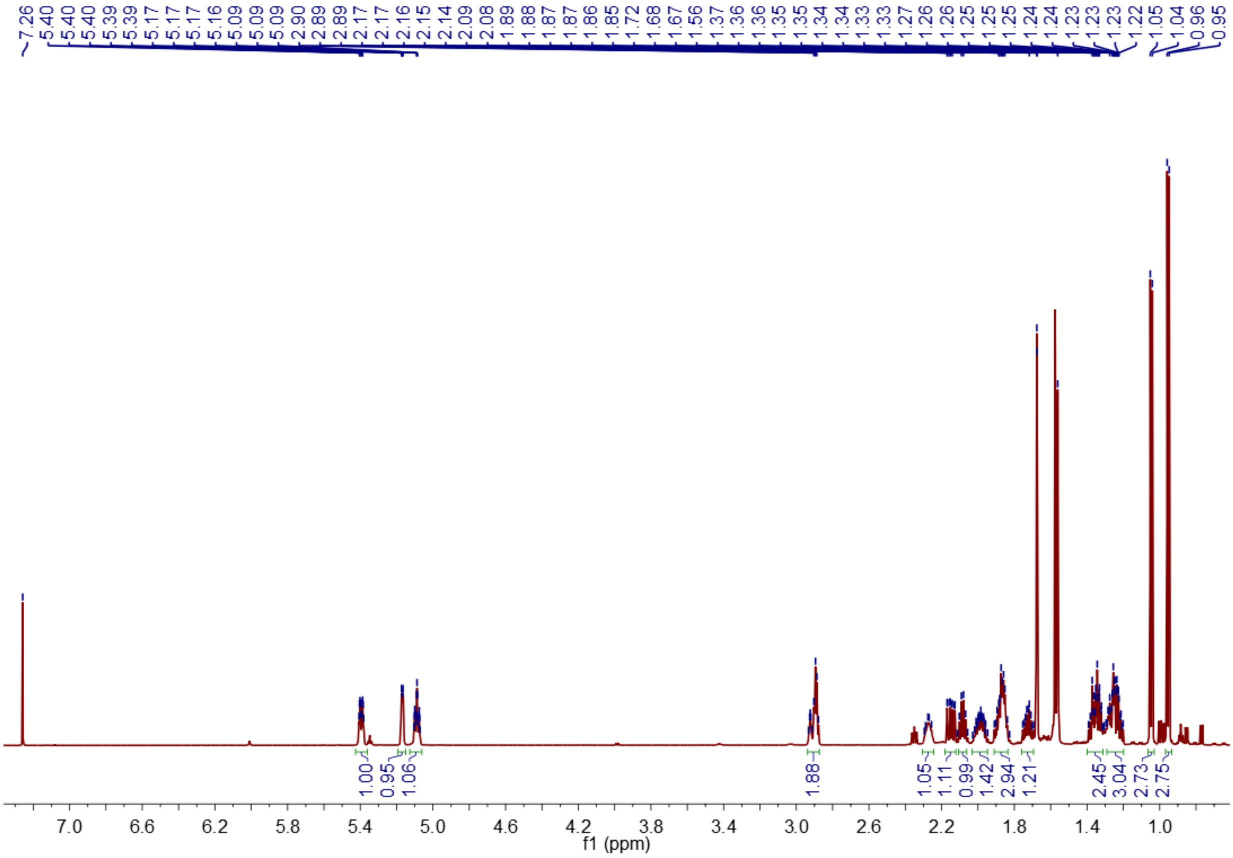

Supplementary Figure 66. The ^{1}H NMR spectrum of **10** in $CDCl_3$.

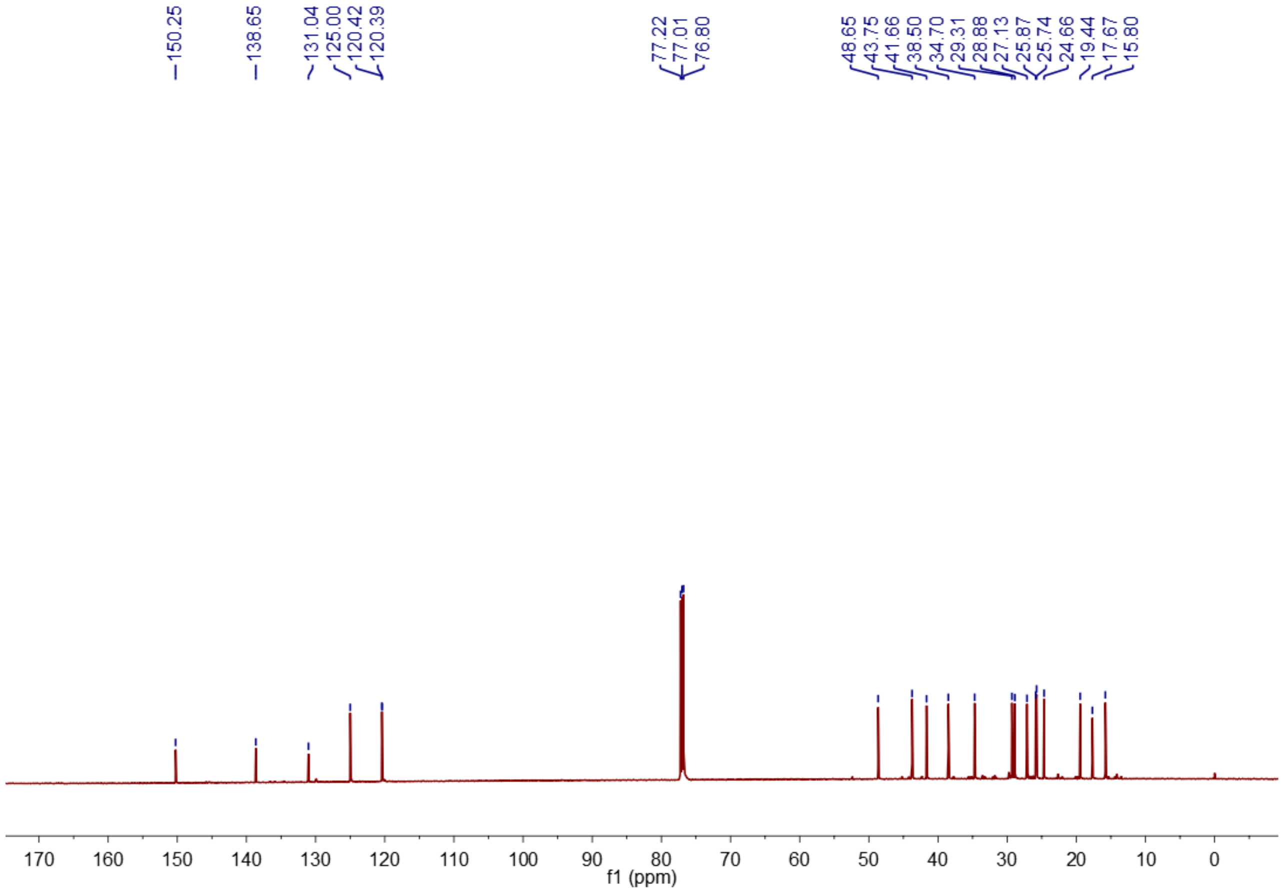

Supplementary Figure 67. The ^{13}C NMR spectrum of **10** in $CDCl_3$.

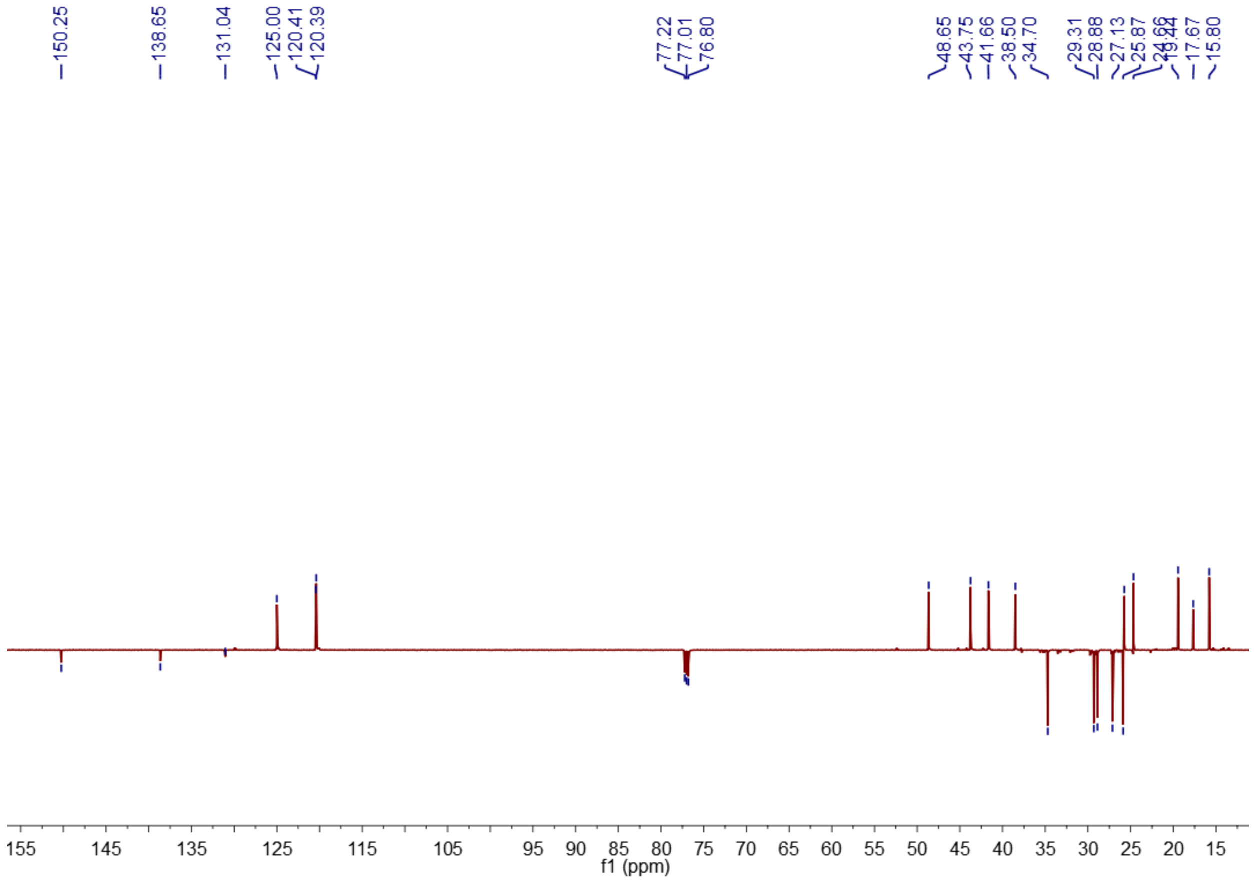

Supplementary Figure 68. The ^{13}C-DEPTQ spectrum of **10** in $CDCl_3$.

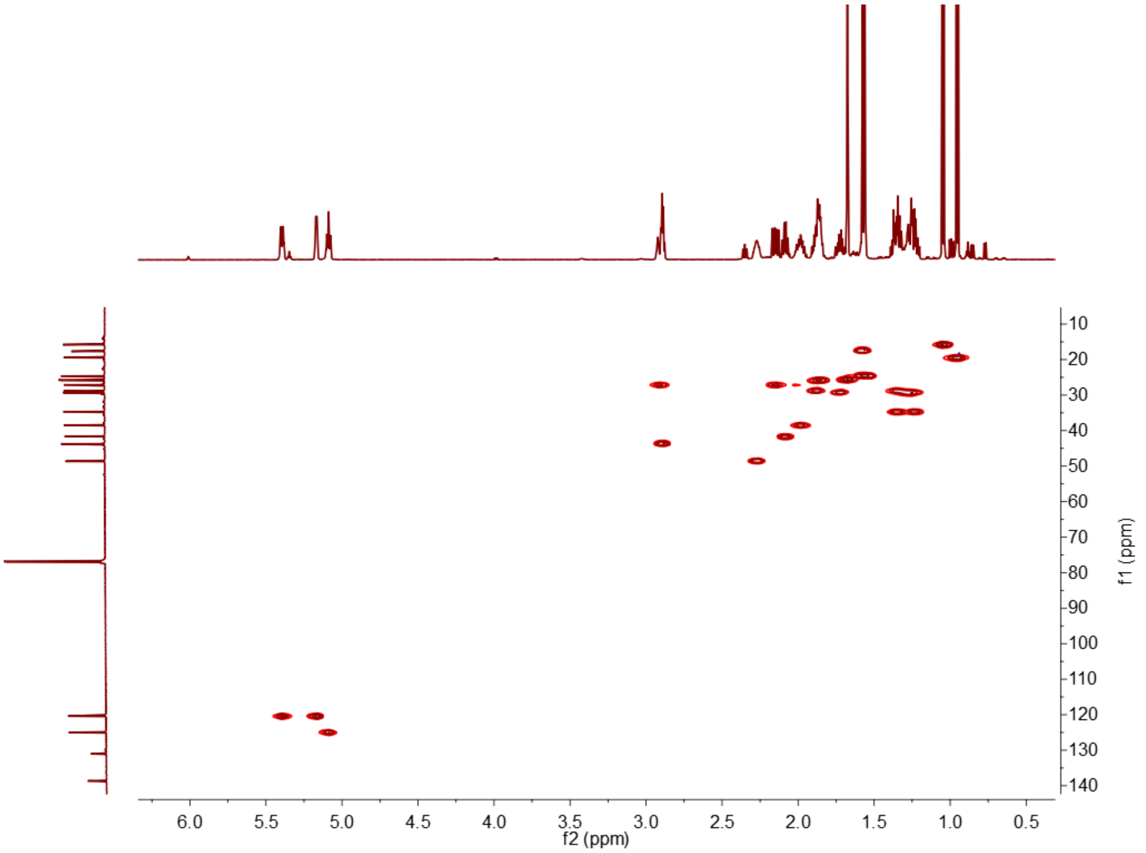

Supplementary Figure 69. The HSQC spectrum of **10** in $CDCl_3$.

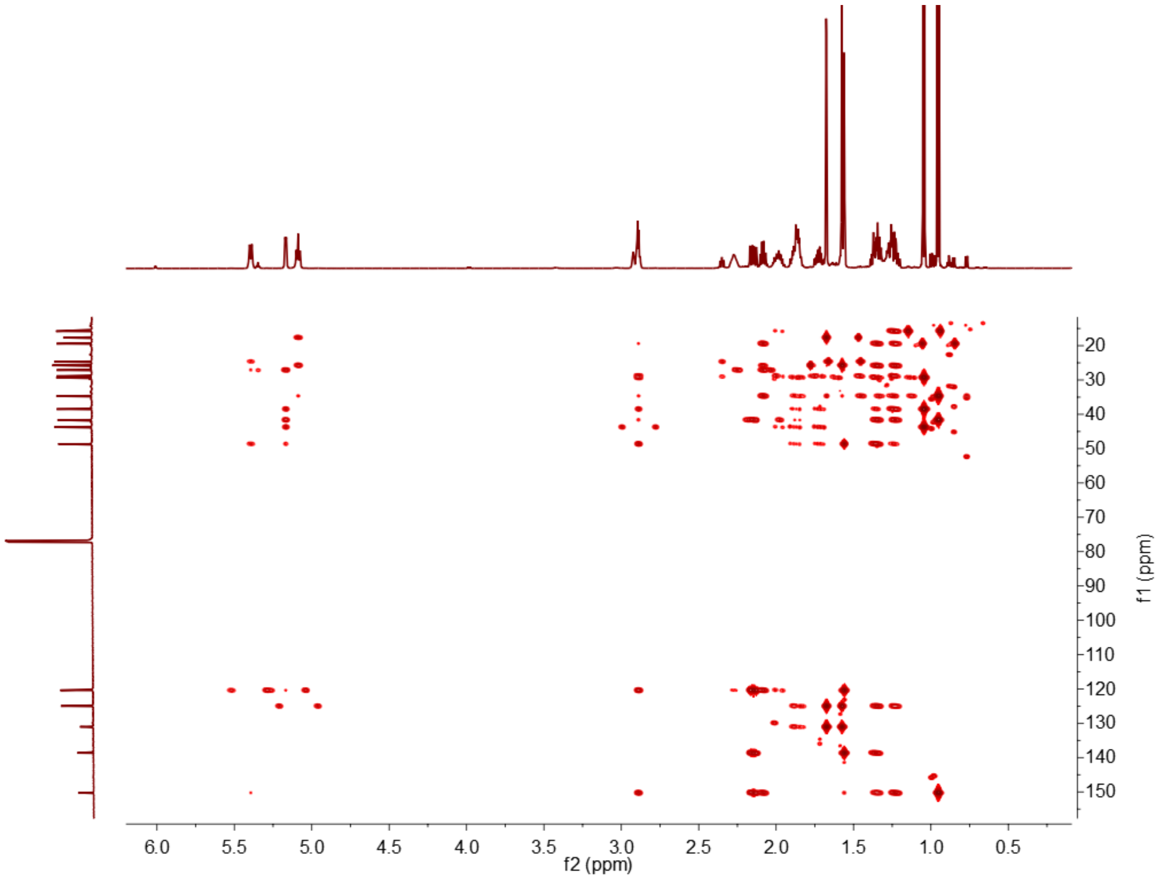

Supplementary Figure 70. The HMBC spectrum of **10** in $CDCl_3$.

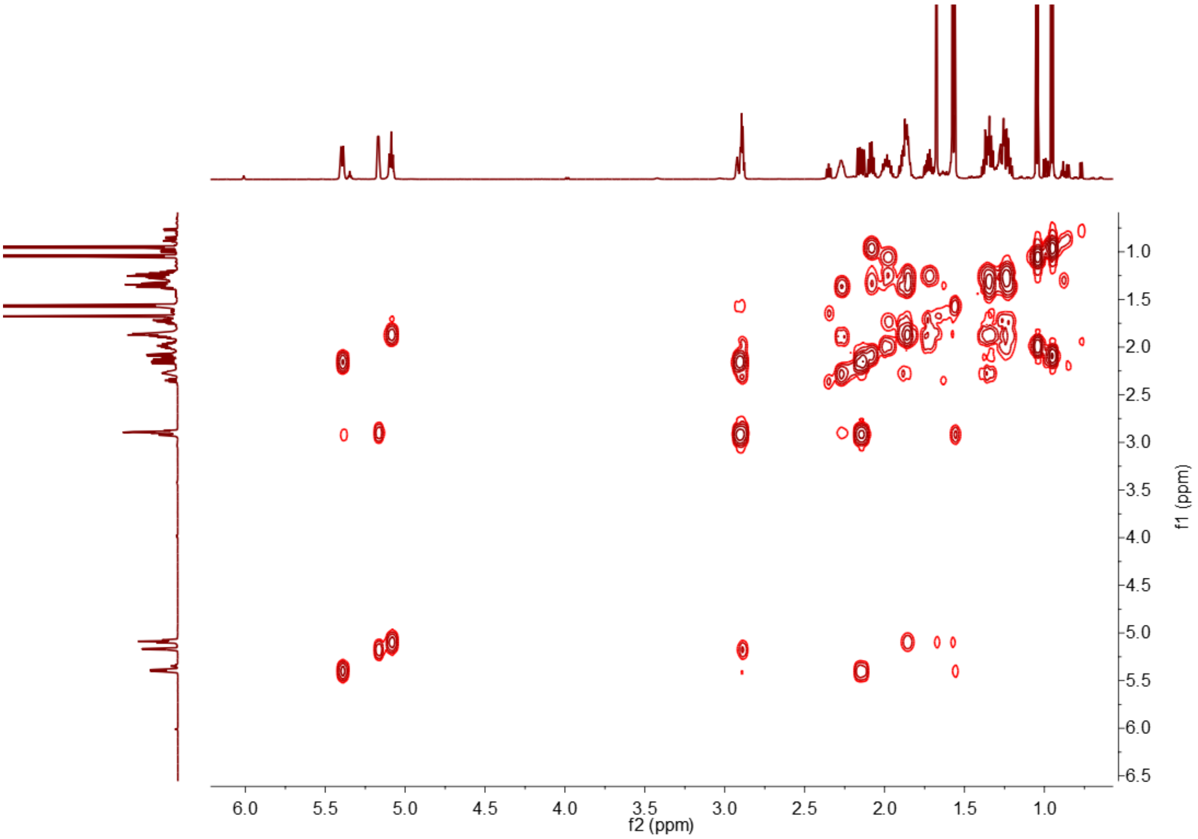

Supplementary Figure 71. The ^{1}H-^{1}H COSY spectrum of **10** in $CDCl_3$.

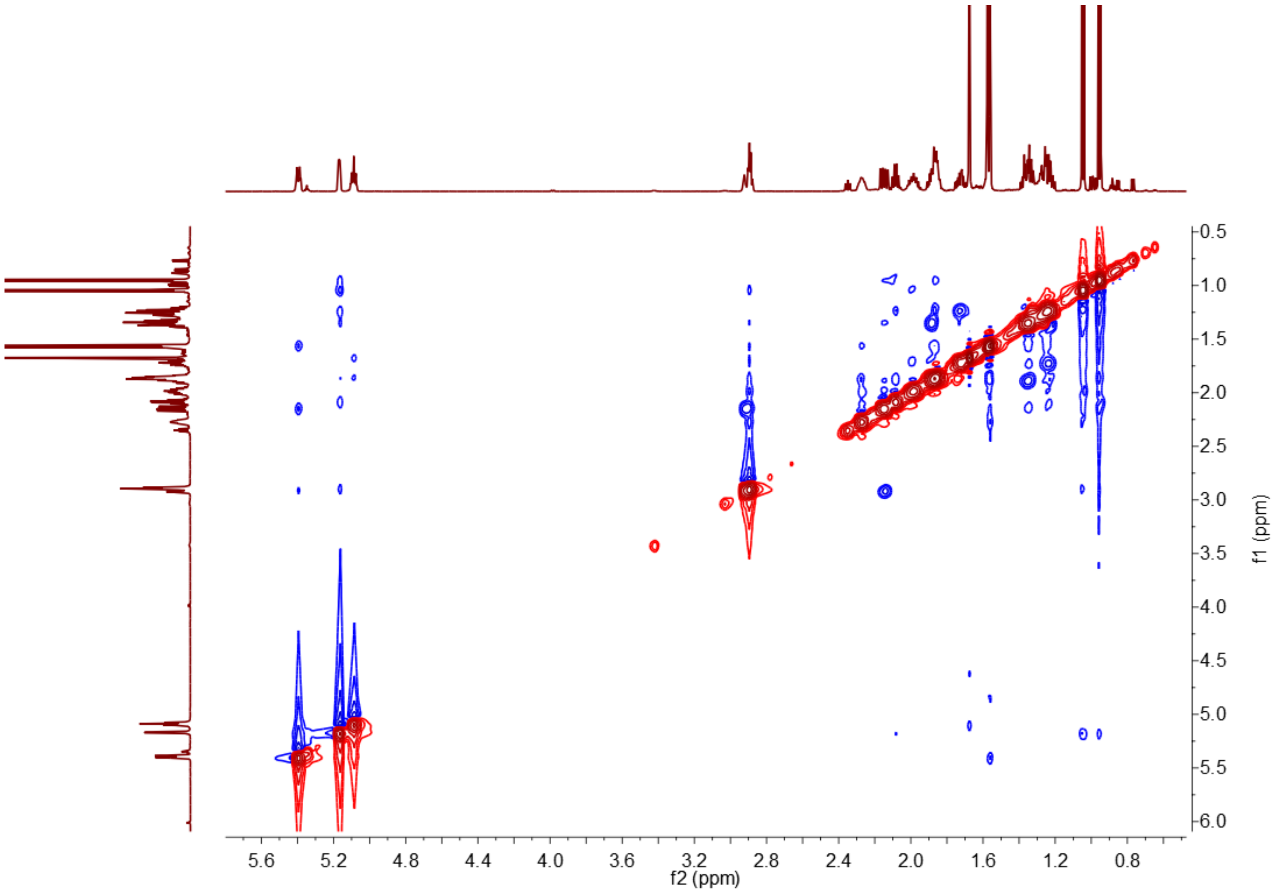

Supplementary Figure 72. The NOESY spectrum of **10** in $CDCl_3$.

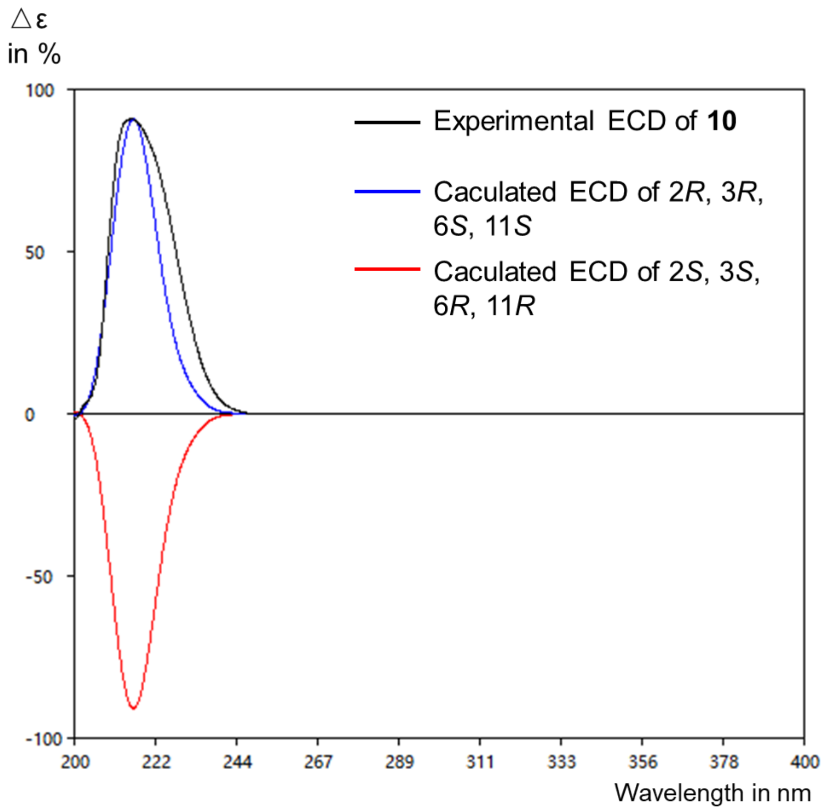

Supplementary Figure 73. The electronic circular dichroism (ECD) spectral analysis of **10** (2*R*, 3*R*, 6*S*, 11*S*).

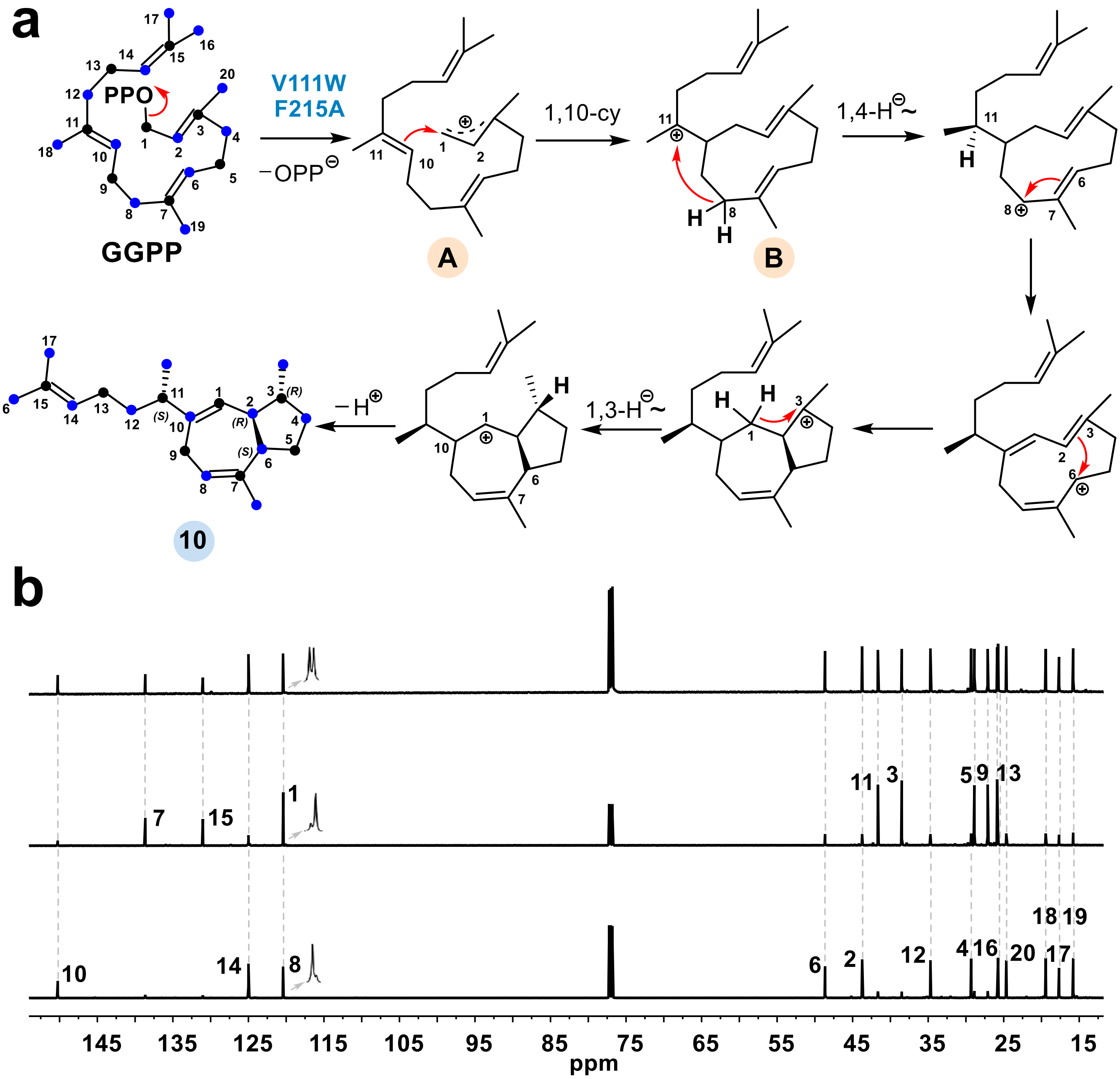

Supplementary Figure 74. The use of ^{13}C-labelled precursors for tracing **10** biosynthesis. **a**, The proposed cyclization mechanism of GGPP to form **10** by VenA^{V111W} or VenA^{F215A}. **b**, ^{13}C NMR spectra of unlabelled and differentially ^{13}C labelled **10**. (***top***) The ^{13}C NMR spectrum of unlabelled **10**; The ^{13}C NMR spectrum of the ^{13}C-labelled **10** obtained from the feeding of (1-^{13}C)-labelled sodium (***middle***) and (2-^{13}C)-labelled sodium acetate (***bottom***) to the recombinant *E. coli* strain of Eco-A^{V111W}D.

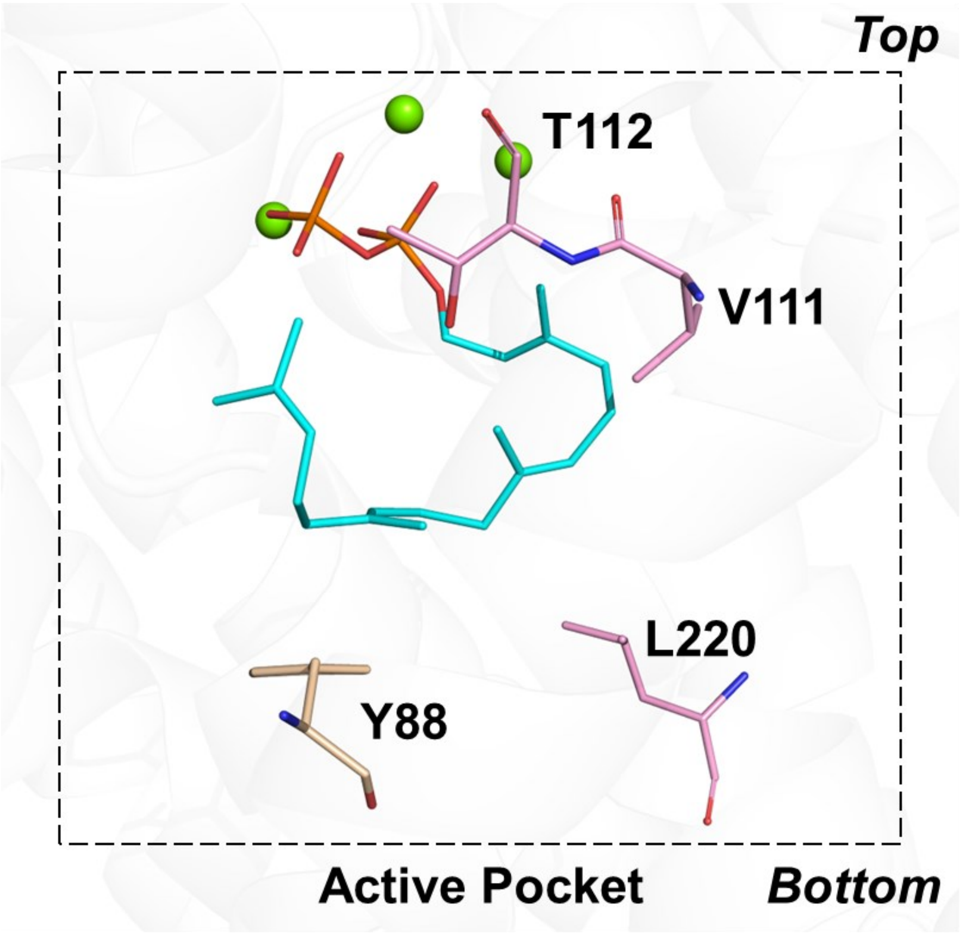

Supplementary Figure 75. The key residues located on top and bottom of the VenA active pocket.

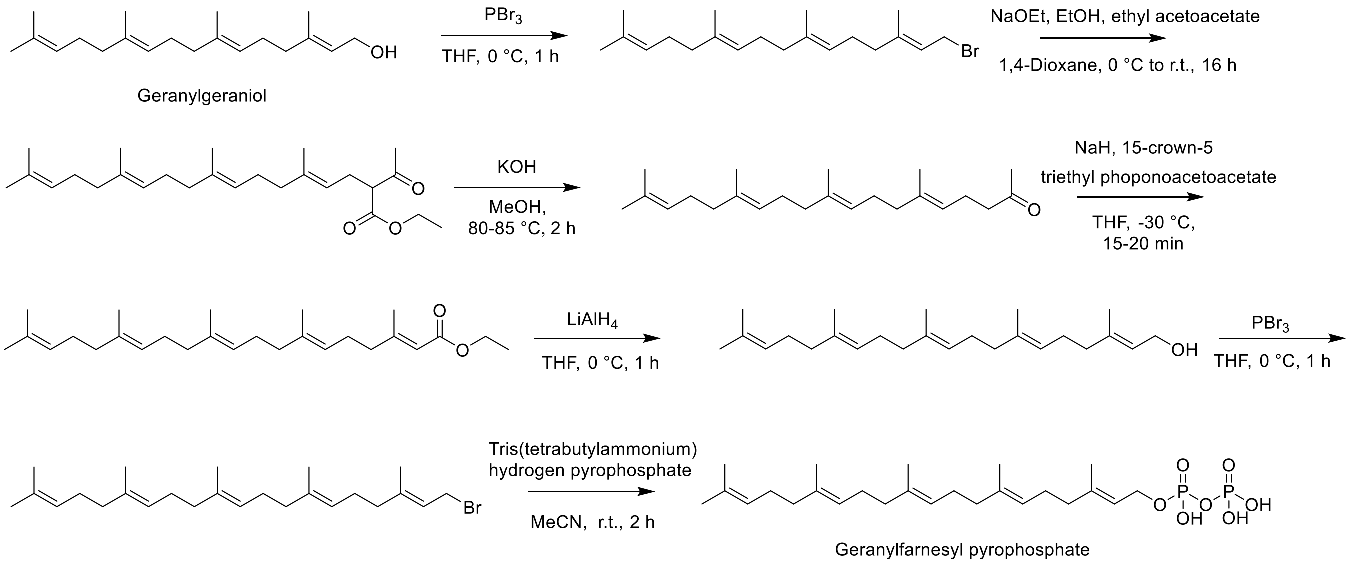

Supplementary Figure 76. Scheme for chemical synthesis of GFPP.

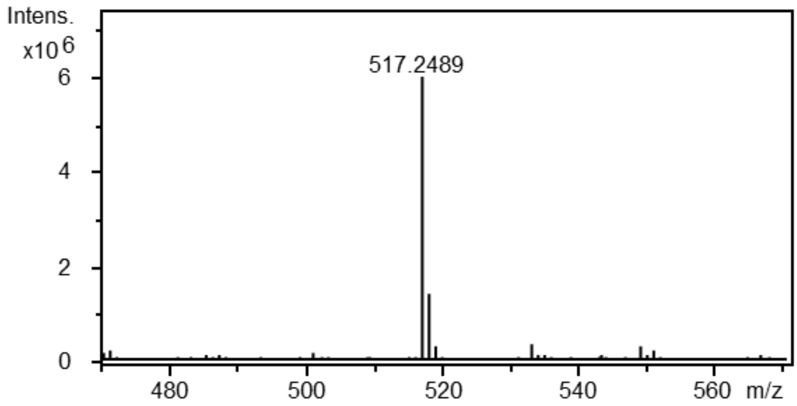

Supplementary Figure 77. The high resolution mass spectrum of synthetic GFPP ($[M-H]^-$: *calc.* 517.2490; *obs.* 517.2489).

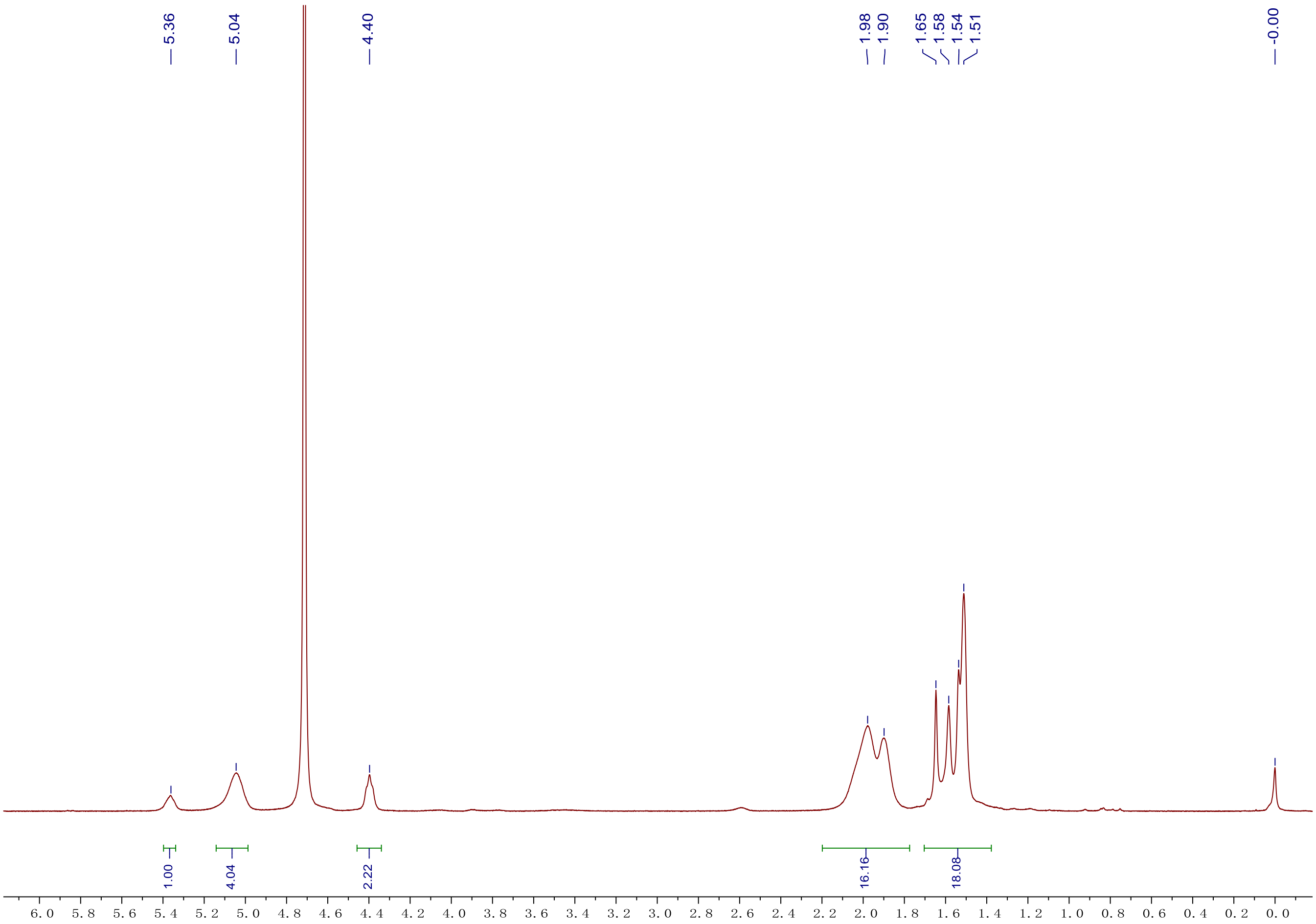

Supplementary Figure 78. The ^1H NMR spectrum of GFPP in D_2O.

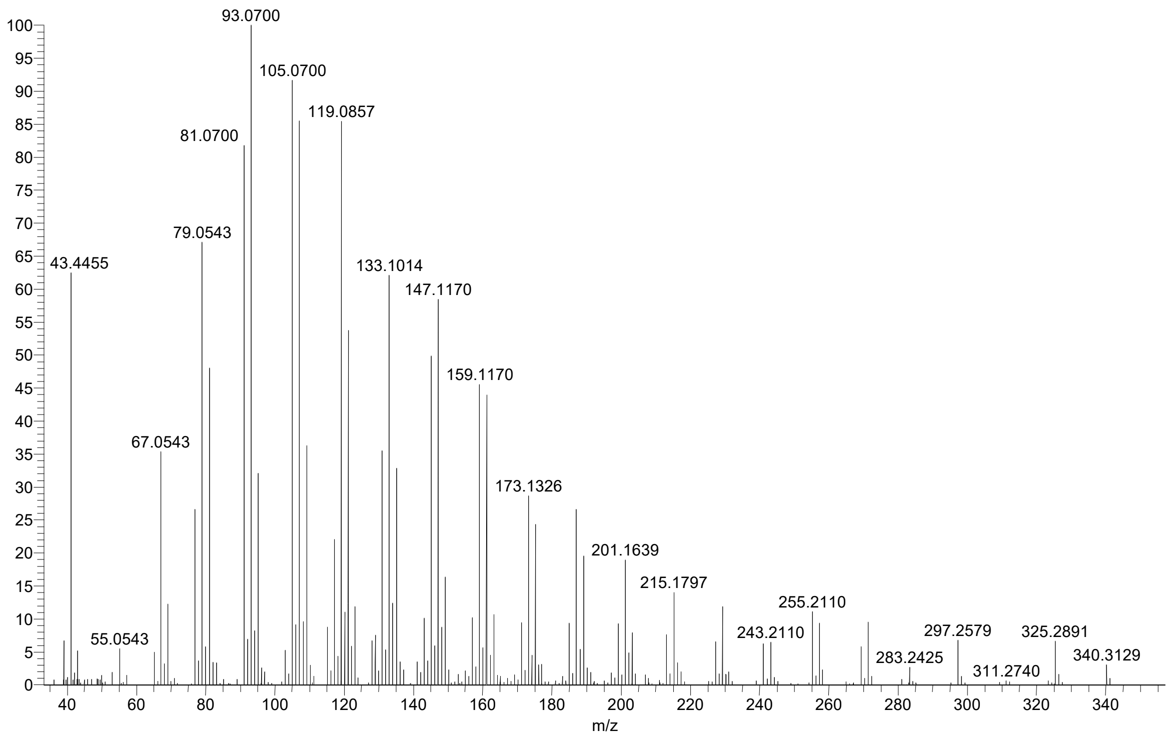

Supplementary Figure 79. The high-resolution GC mass spectrum of **19** (*calc.* 340.3125; *obs.* 340.3129).

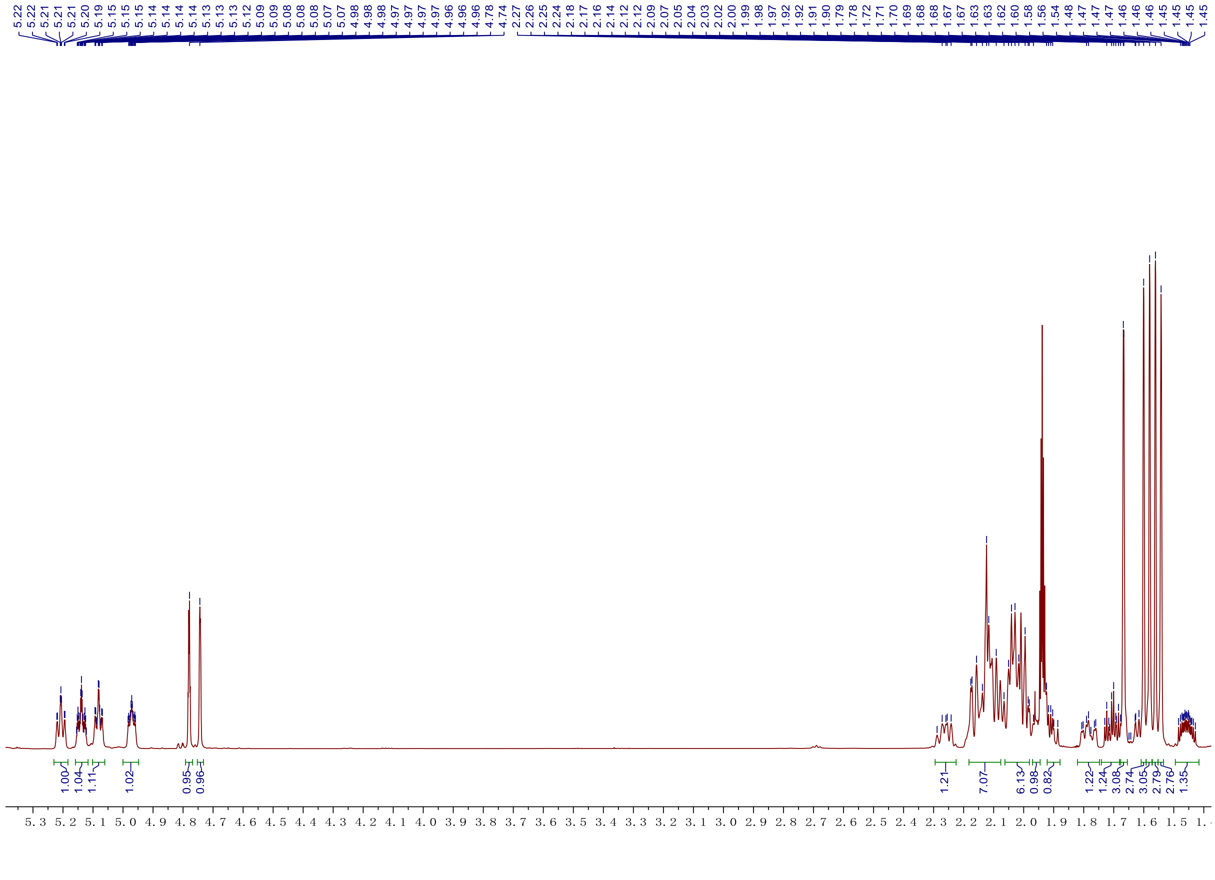

Supplementary Figure 80. The ^{1}H NMR spectrum of **19** in CD_3CN.

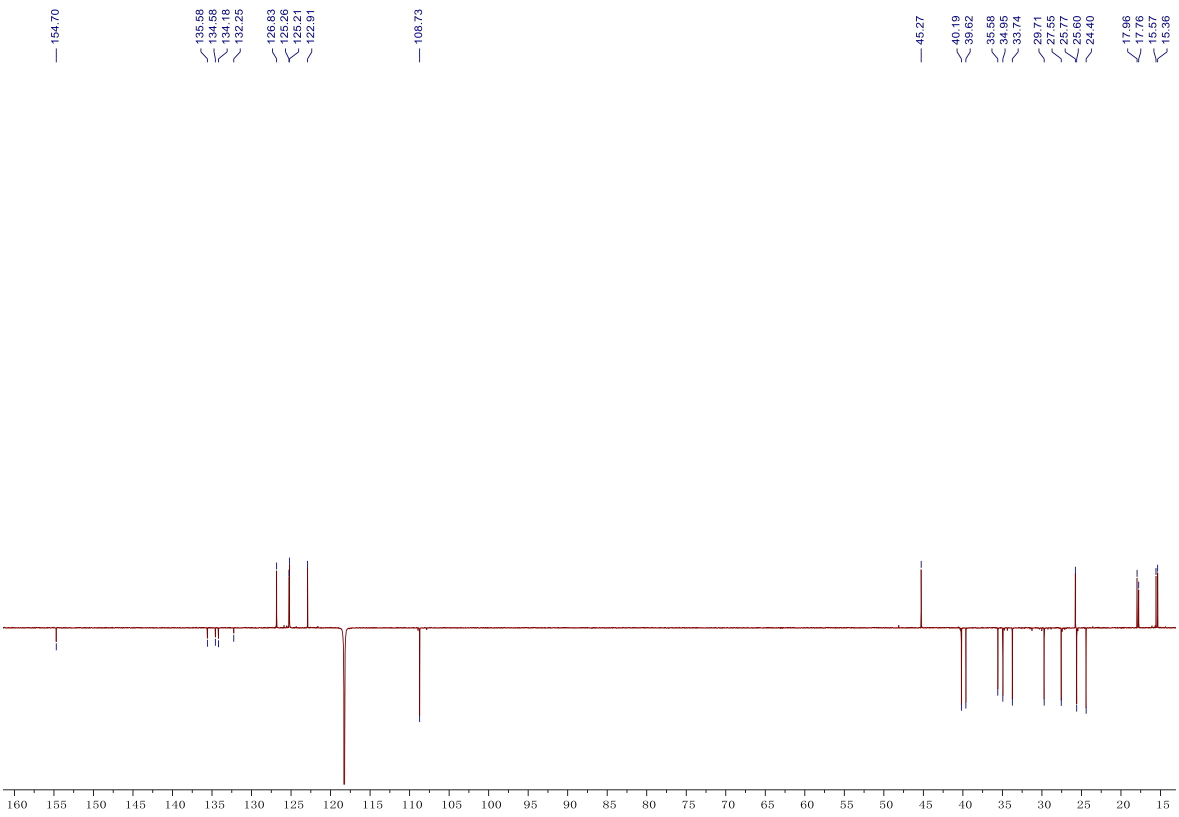

Supplementary Figure 81. The ^{13}C-DEPTQ spectrum of **19** in CD_3CN.

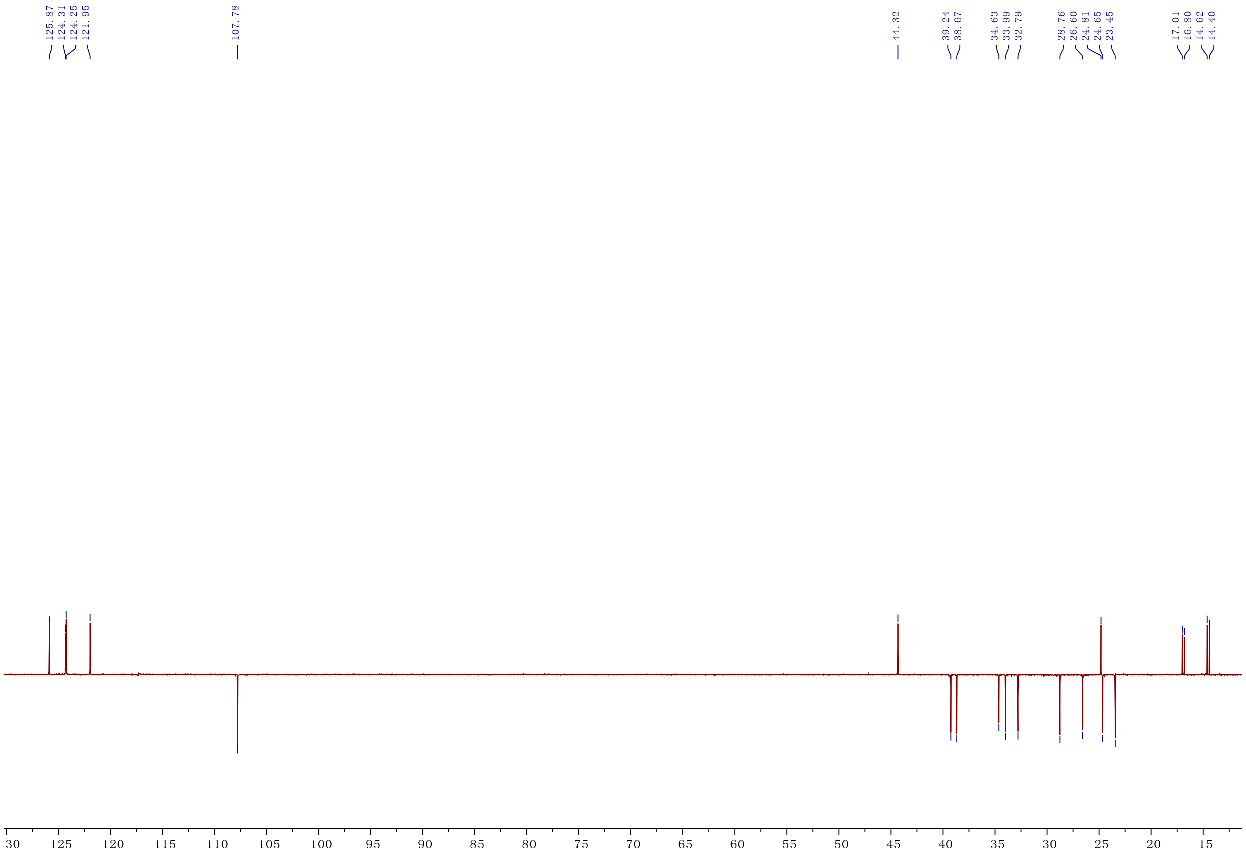

Supplementary Figure 82. The ^{13}C-DEPT 135° spectrum of **19** in CD_3CN.

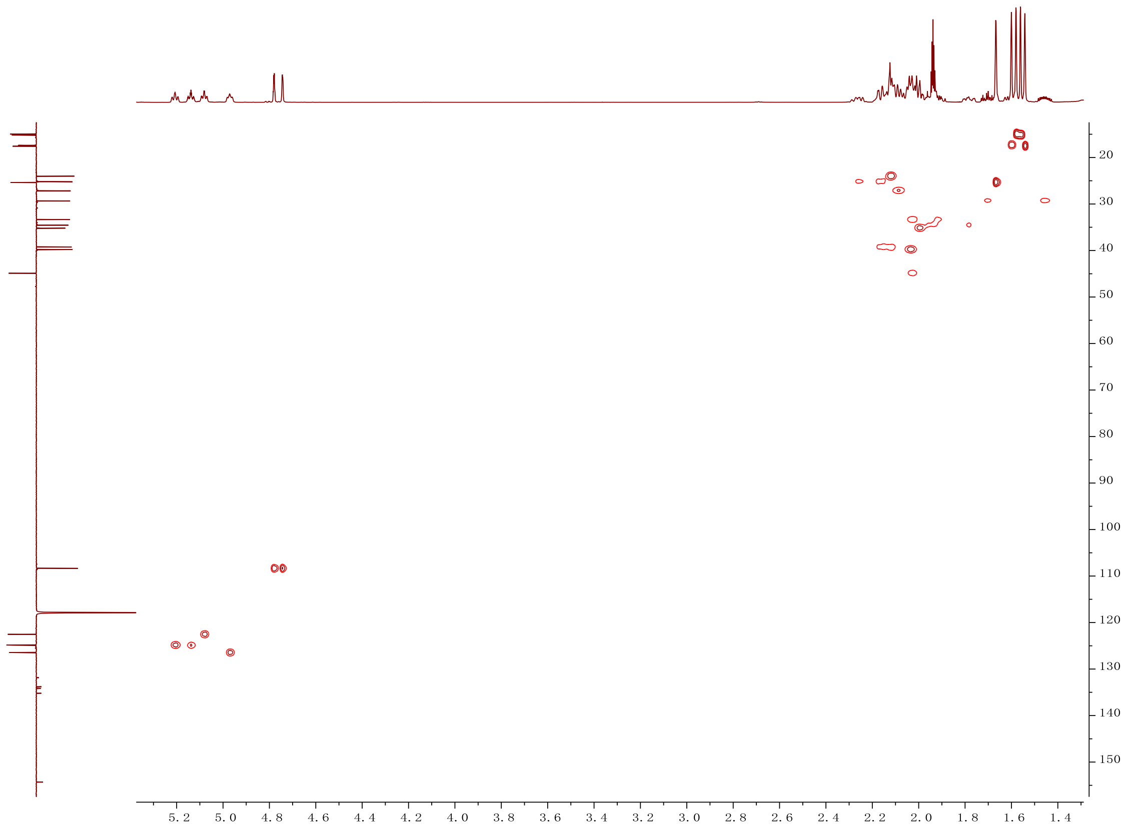

Supplementary Figure 83. The HSQC spectrum of **19** in CD_3CN.

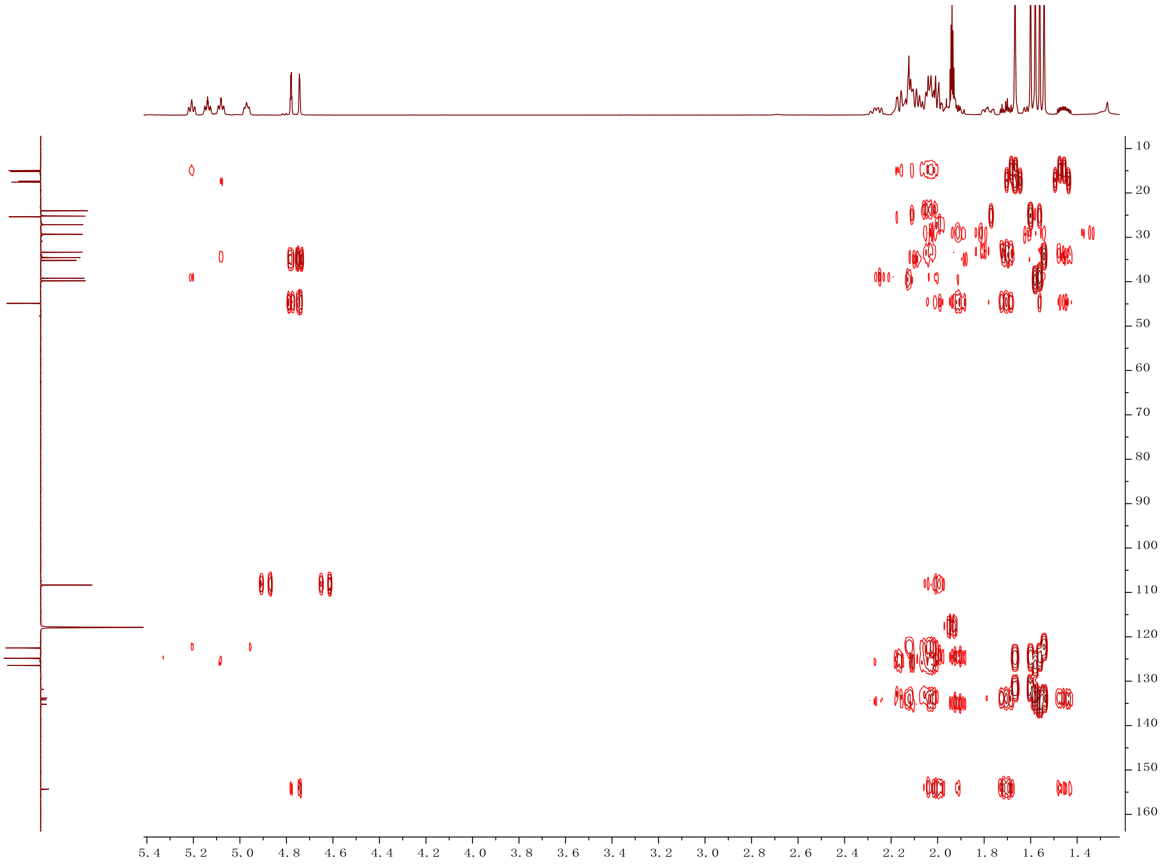

Supplementary Figure 84. The HMBC spectrum of **19** in CD_3CN.

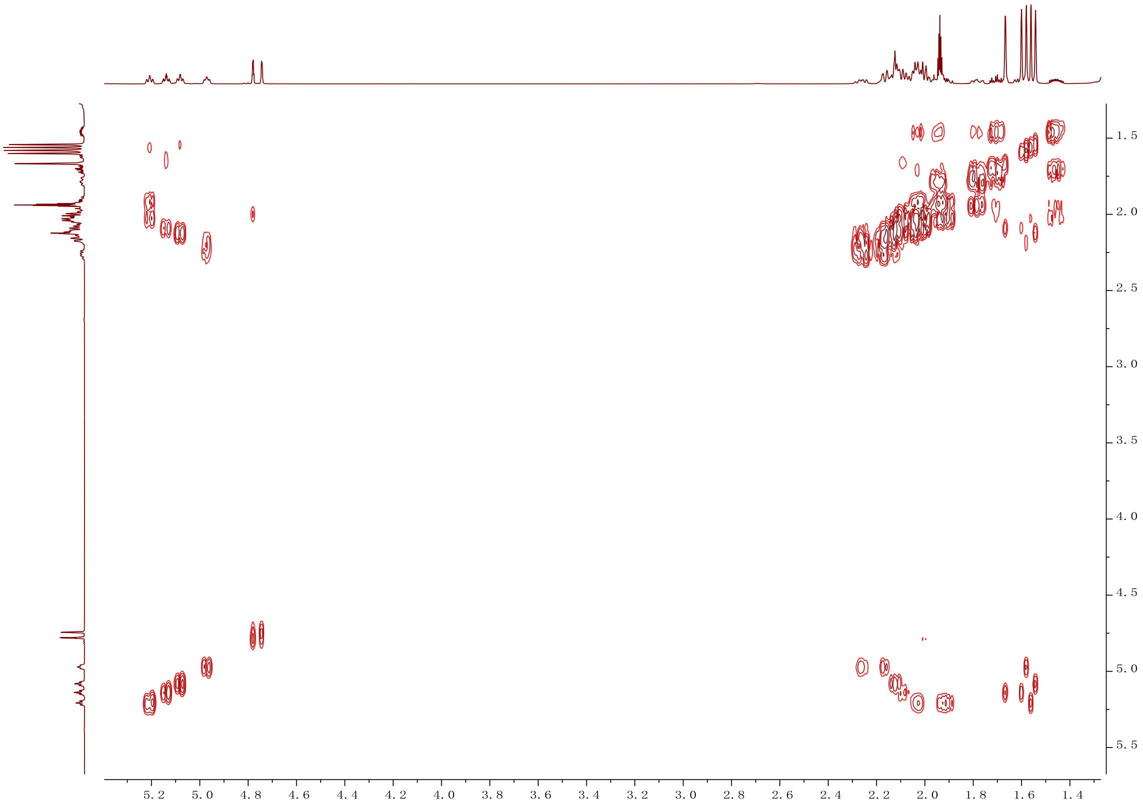

Supplementary Figure 85. The ^{1}H-^{1}H COSY spectrum of **19** in CD_3CN.

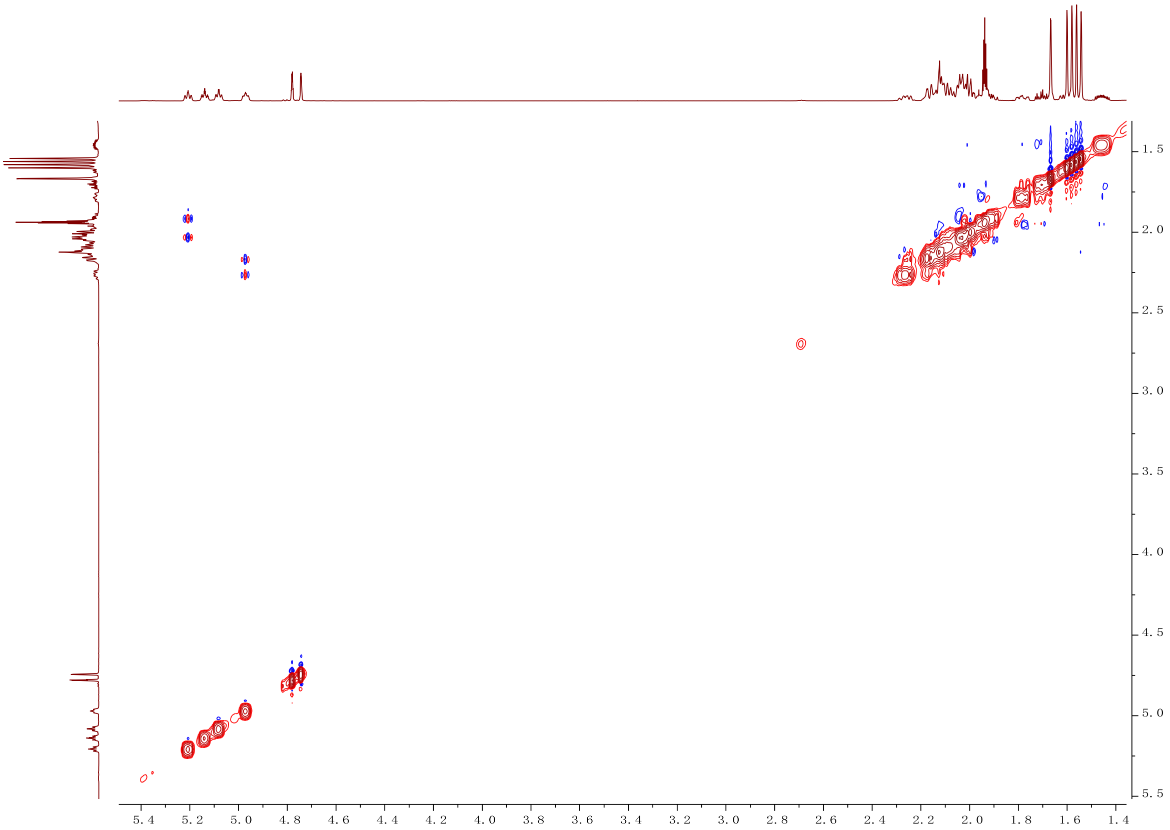

Supplementary Figure 86. The NOESY spectrum of **19** in CD_3CN.

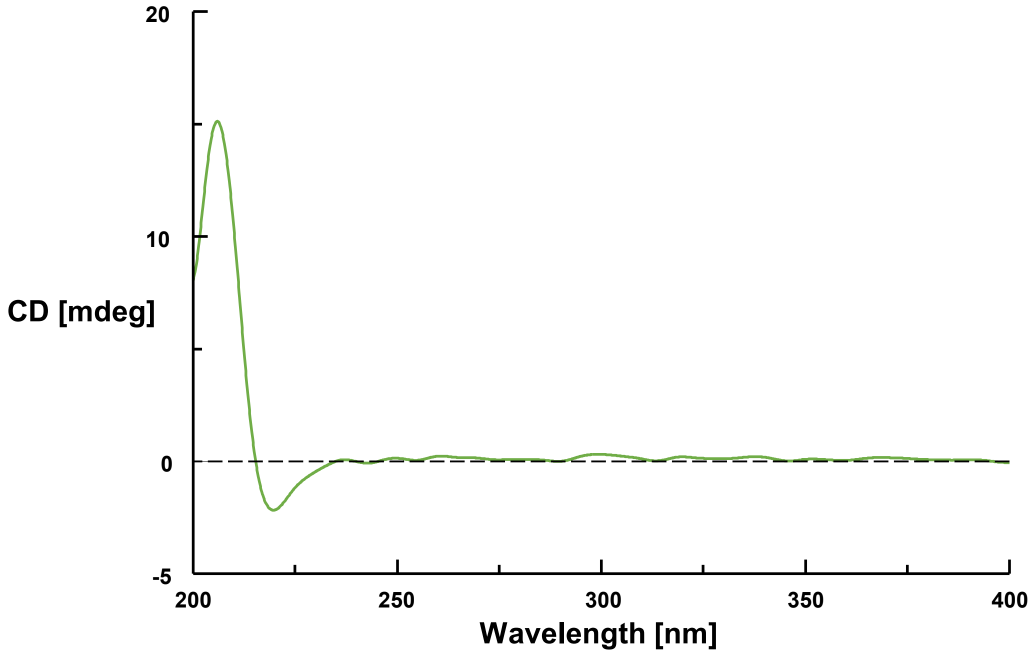

Supplementary Figure 87. The CD spectrum of **19**.

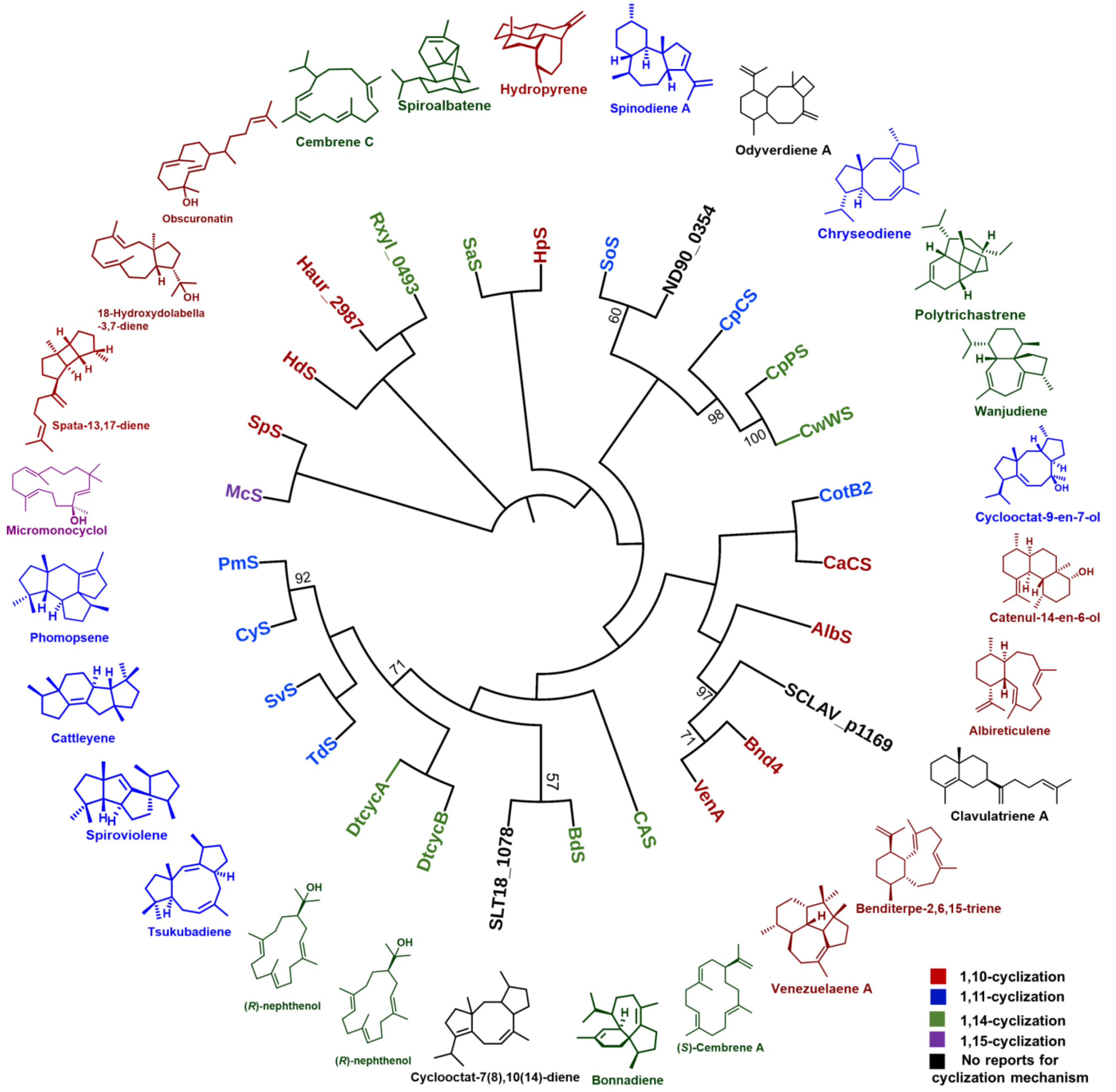

Supplementary Figure 88. The phylogenetic tree of VenA with the identified bacterial DTSs using the GGPP as natural substrate by applying the maximum likelihood method based on the Jones-Taylor-Thornton model with the replicates of 1000 times. The bootstrap consensus tree inferred from 1000 replicates is taken to represent the evolutionary history of the taxa analyzed. Bootstrap values > 50% (based on 1000 resampled trials) are given at nodes. *Note*: The first cyclization step mediated by DTSs are marked by different colors. DtcycB also catalyzed the initial 1,15-cyclization of GGPP to form another main product (4*E*,8*E*,12*E*)-2,2,5,9,13-pentamethylcyclopentadeca-4,8,12-trien-1-ol. The GenBank accession numbers of the proteins are listed as follows: VenA (venezuelaene A synthase, QGF19026.1), BdS (bonnadiene synthase, WP_030432512.1), Bnd4 (benditerpe-2,6,15-triene synthase, WP_239771469.1),

AlbS (albireticulene synthase, PAU47876.1); CaCS (catenul-14-en-6-ol synthase, WP_015792165), CAS (cembrene A synthase, WP_030430753.1), CotB2 (cyclooctat-9en-7-ol synthase, BAI44338.1), CpPS (polytrichastrene synthase, WP_073293738.1), CpCS (chryseodiene synthase, WP_073290622.1), CwWS (wanjudiene Synthase, WP_089795910.1), CyS (cattleyene synthase, WP_014150548.1), DtcycA ((*R*)-nephthenol synthase, M1V9Q0.1), DtcycB ((*R*)-nephthenol synthase, M1VDX3.1), Haur_2987 (obscuronatin synthase, WP_012190524.1), HdS (18-hydroxydolabella-3,7-diene synthase, WP_012789469.1), HpS (hydropyrene synthase, WP_003963279.1), ND90_0354 (odyverdiene A synthase, BAP82229.1), PmS (phomopsene synthase, WP_030431358.1), Rxyl_0493 (cembrene C synthase, WP_041328593.1), SCLAV_p1169 (clavulatriene A synthase, EFG04655.1), SaS (spiroalbatene synthase, WP_030426588.1), SoS (spinodiene synthase, WP_010314578.1), SpS (spata-13,17-diene synthase, WP_228430292.1), SvS (spiroviolene synthase, WP_030261827.1), SLT18_1078 (cyclooctat-7(8),10(14)-diene synthase, BAP82203.1), TdS (tsukubadiene synthase, EIF90392.1), McS (micromonocyclol synthase, WP_091046421.1).

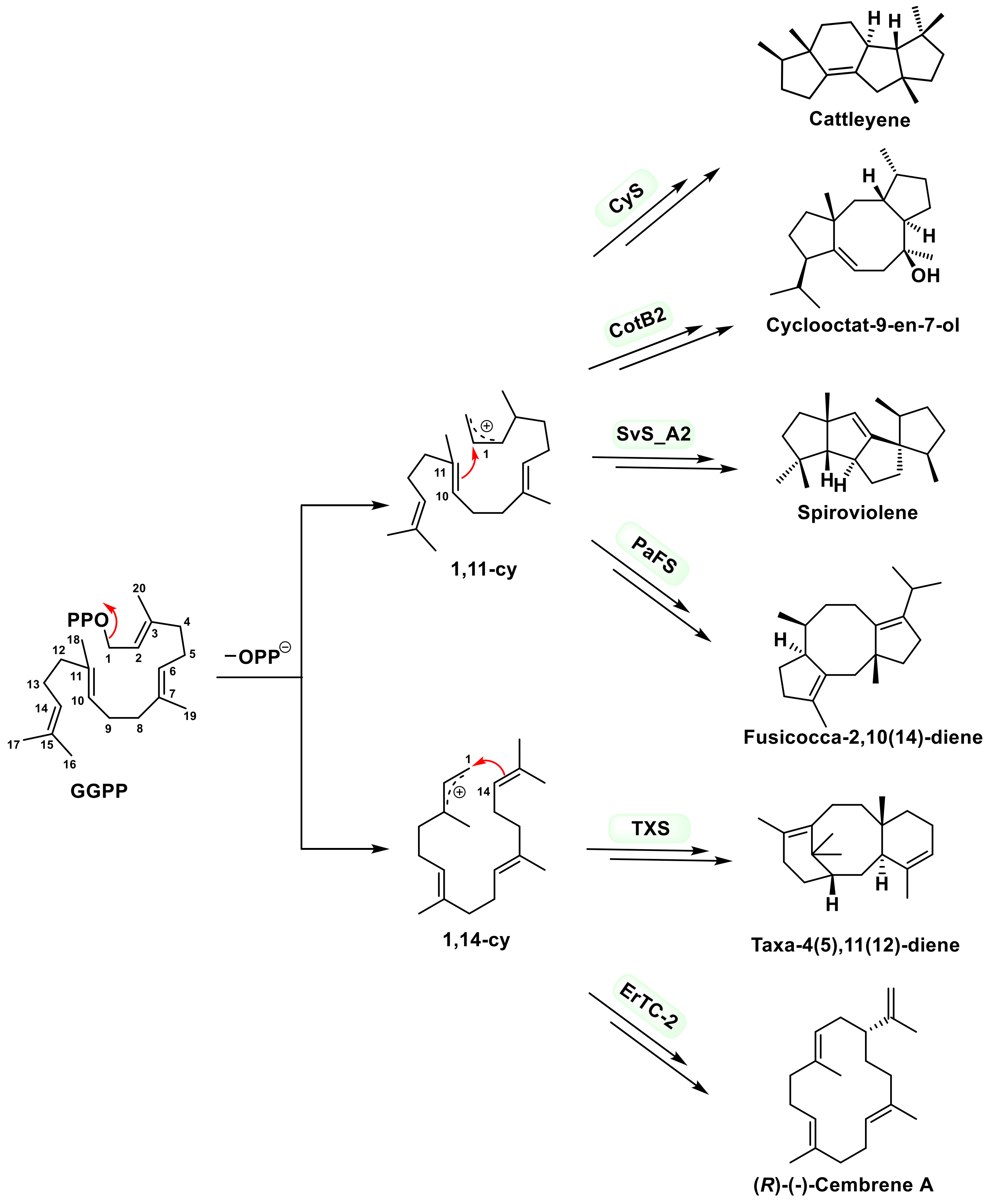

Supplementary Figure 89. The initial cyclization step of GGPP catalyzed by CyS, CotB2, SvS-A2, PaFS, TXS (taxadiene synthase) and ErTC-2.

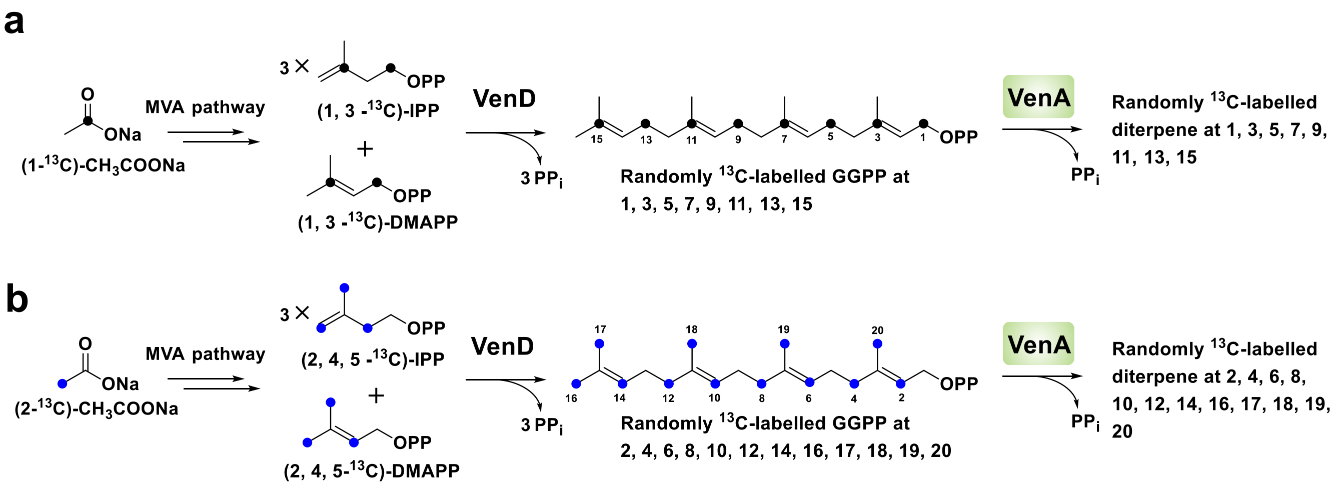

Supplementary Figure 90. The schematic strategies for generating the ^{13}C-labelled GGPP by feeding (1-^{13}C)-CH_3COONa (**a**) or (2-^{13}C)-CH_3COONa (**b**) *in vivo* for tracing diterpenes biosynthesis in this study.

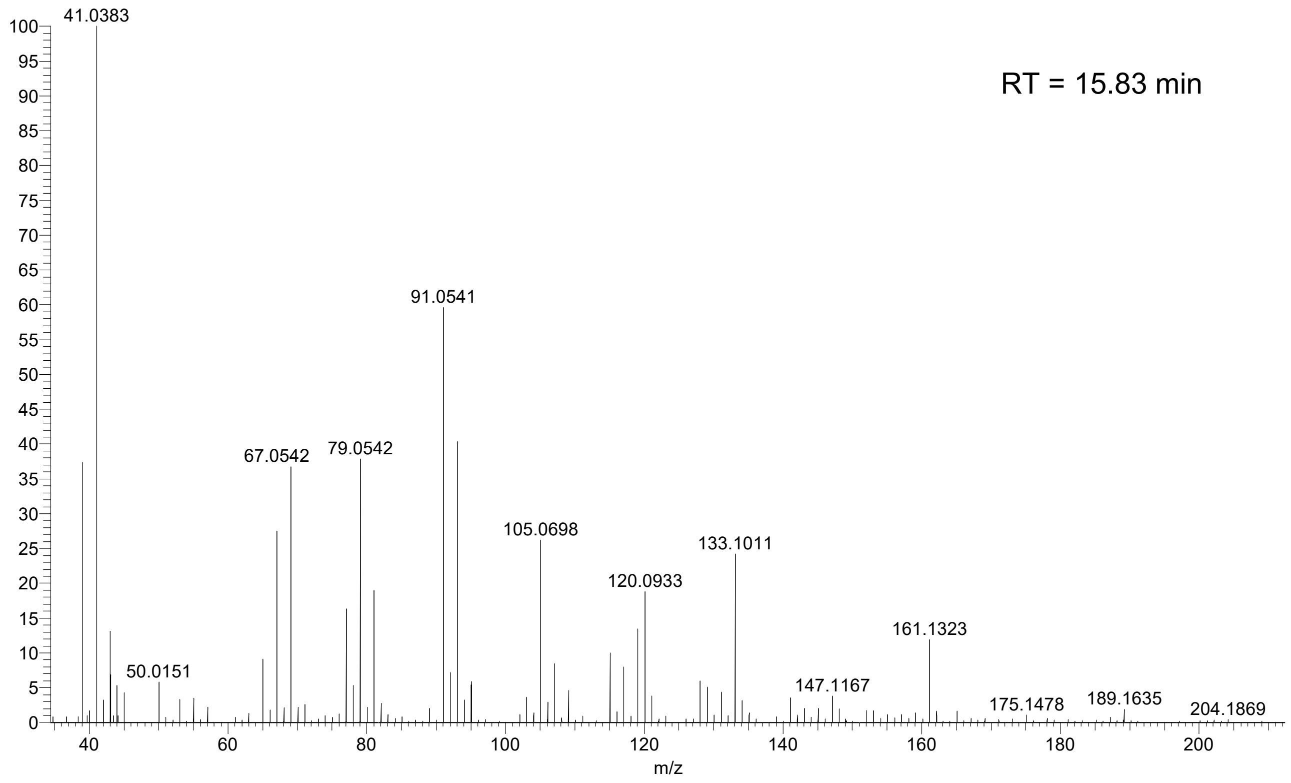

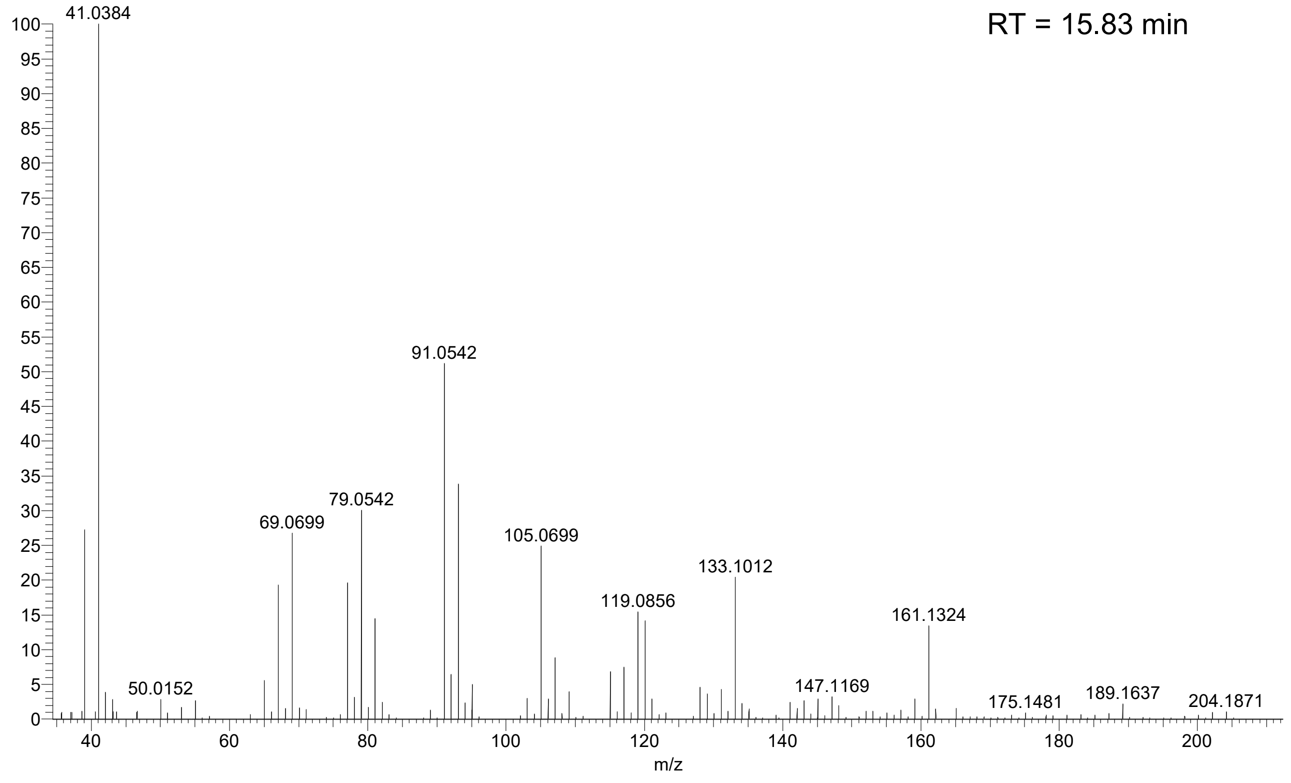

Supplementary Figure 91. The high resolution GC mass spectra of **4** (*top*) and the *β*-farnesene authentic standard (*bottom*).

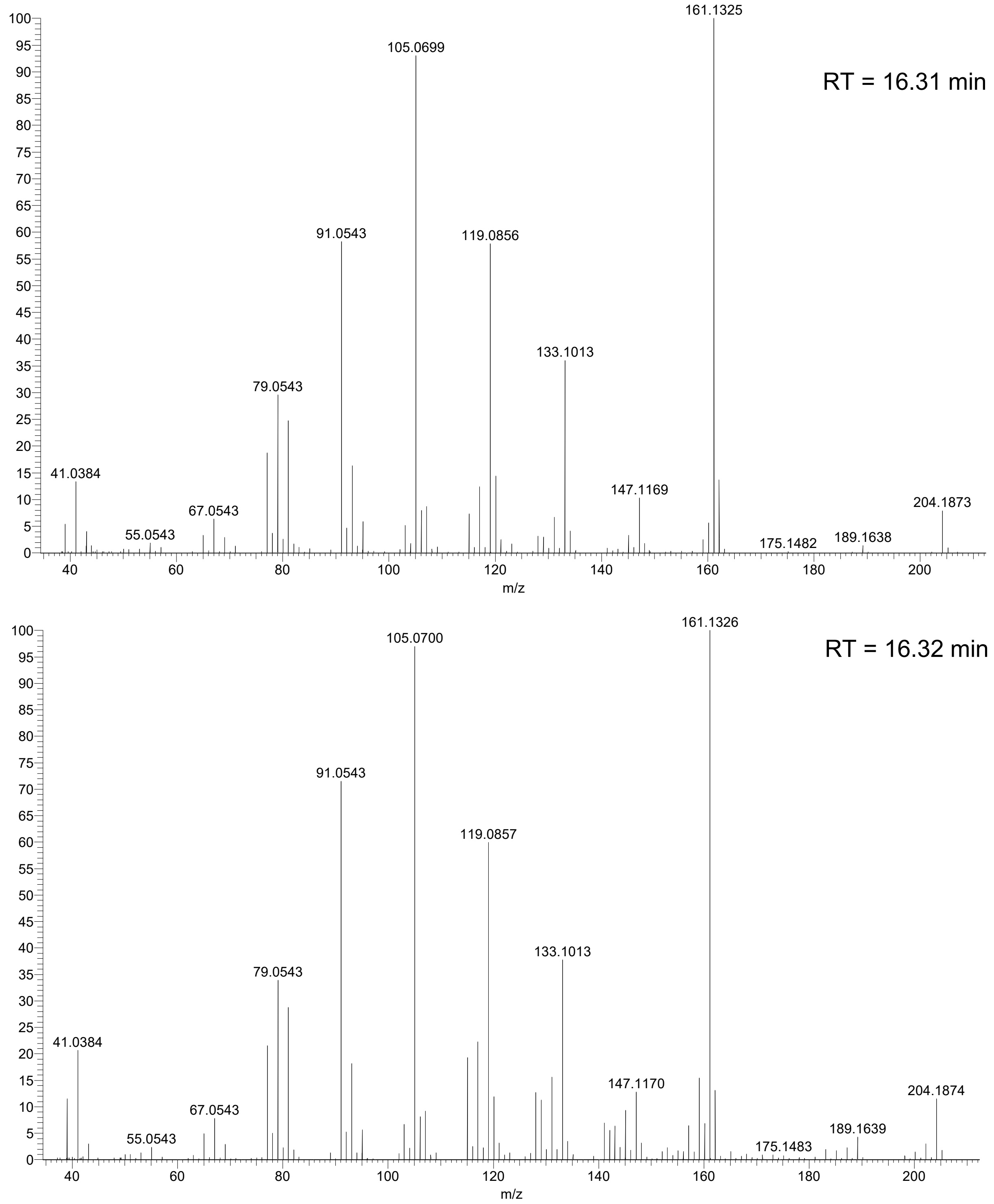

Supplementary Figure 92. The high resolution GC mass spectra of **5** (*top*) and the germacrene D authentic standard (*bottom*).

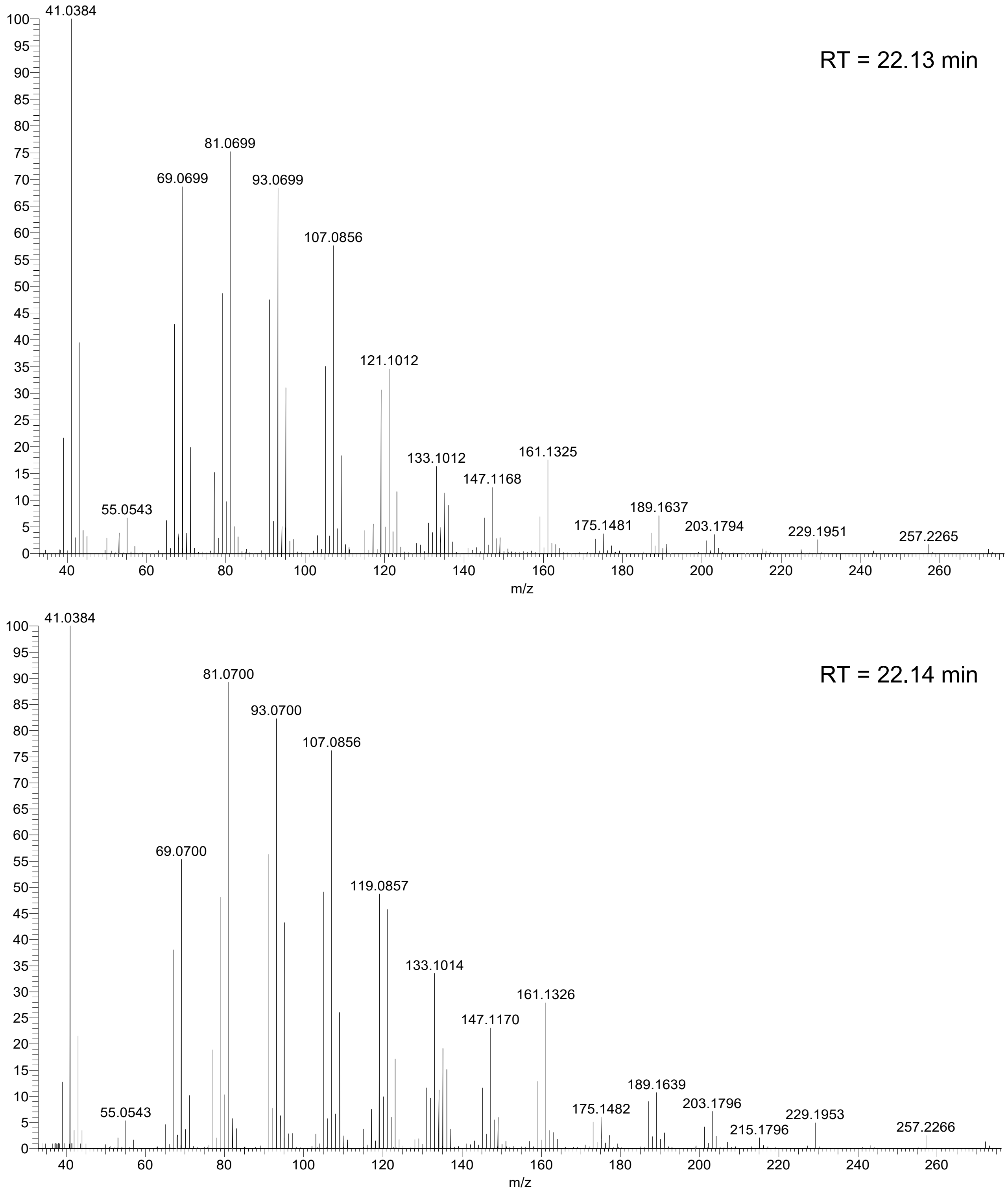

Supplementary Figure 93. The high resolution GC mass spectra of **13** (*top*) and the geranyllinalool authentic standard (*bottom*).

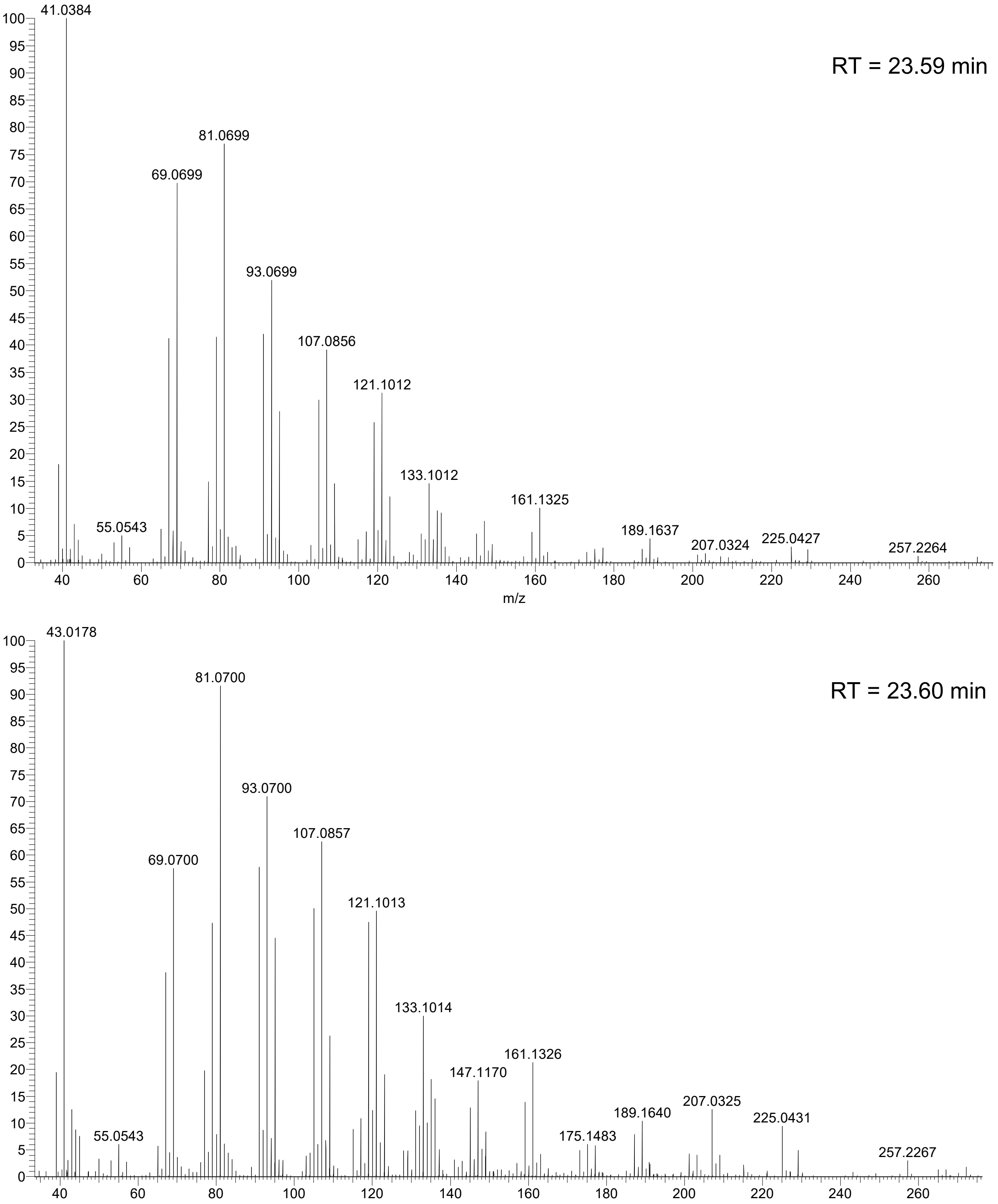

Supplementary Figure 94. The high resolution GC mass spectra of **14** (*top*) and the geranylgeraniol authentic standard (*bottom*).

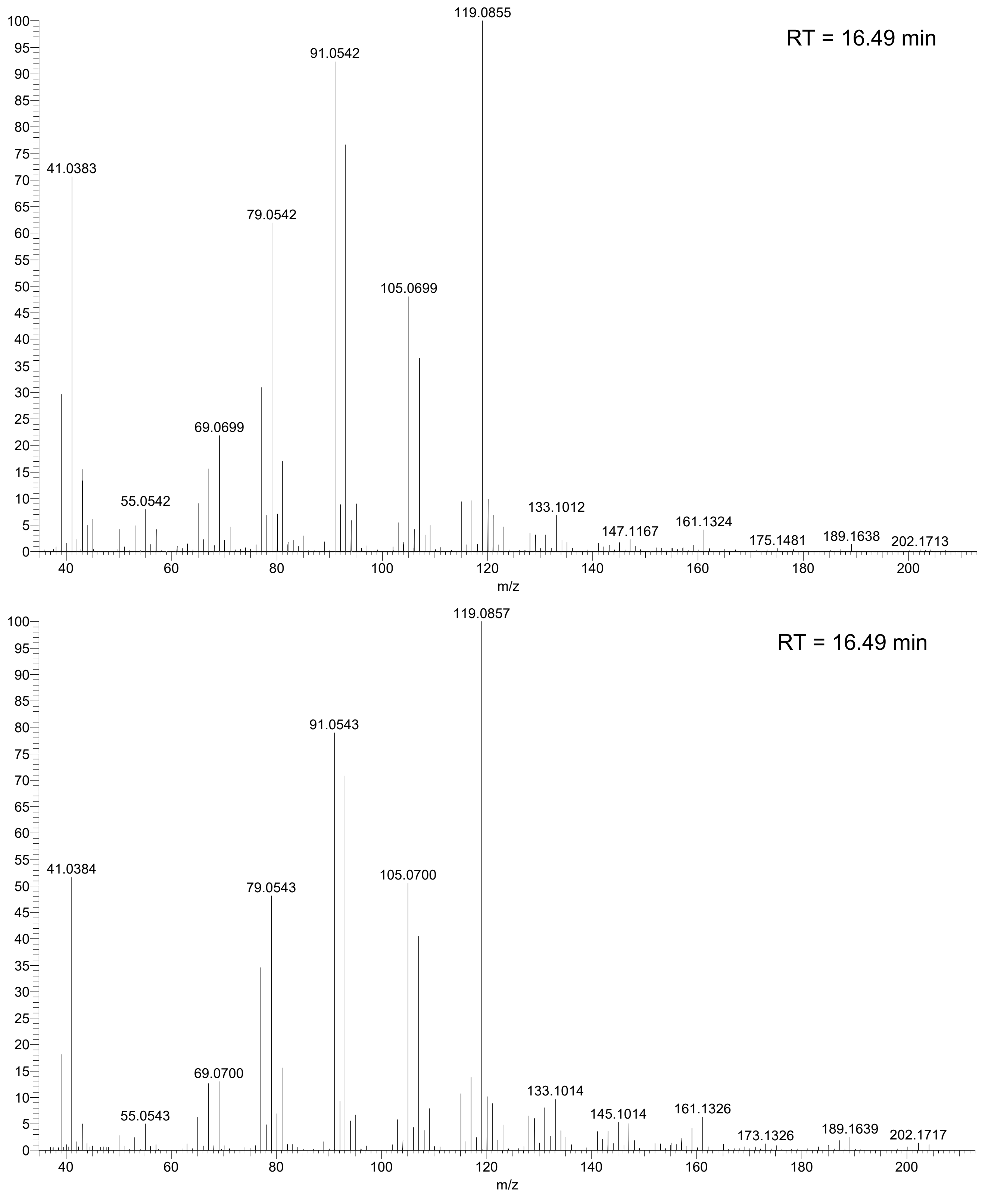

Supplementary Figure 95. The high resolution GC mass spectra of **15** (*top*) and the *α*-farnesene authentic standard (*bottom*).

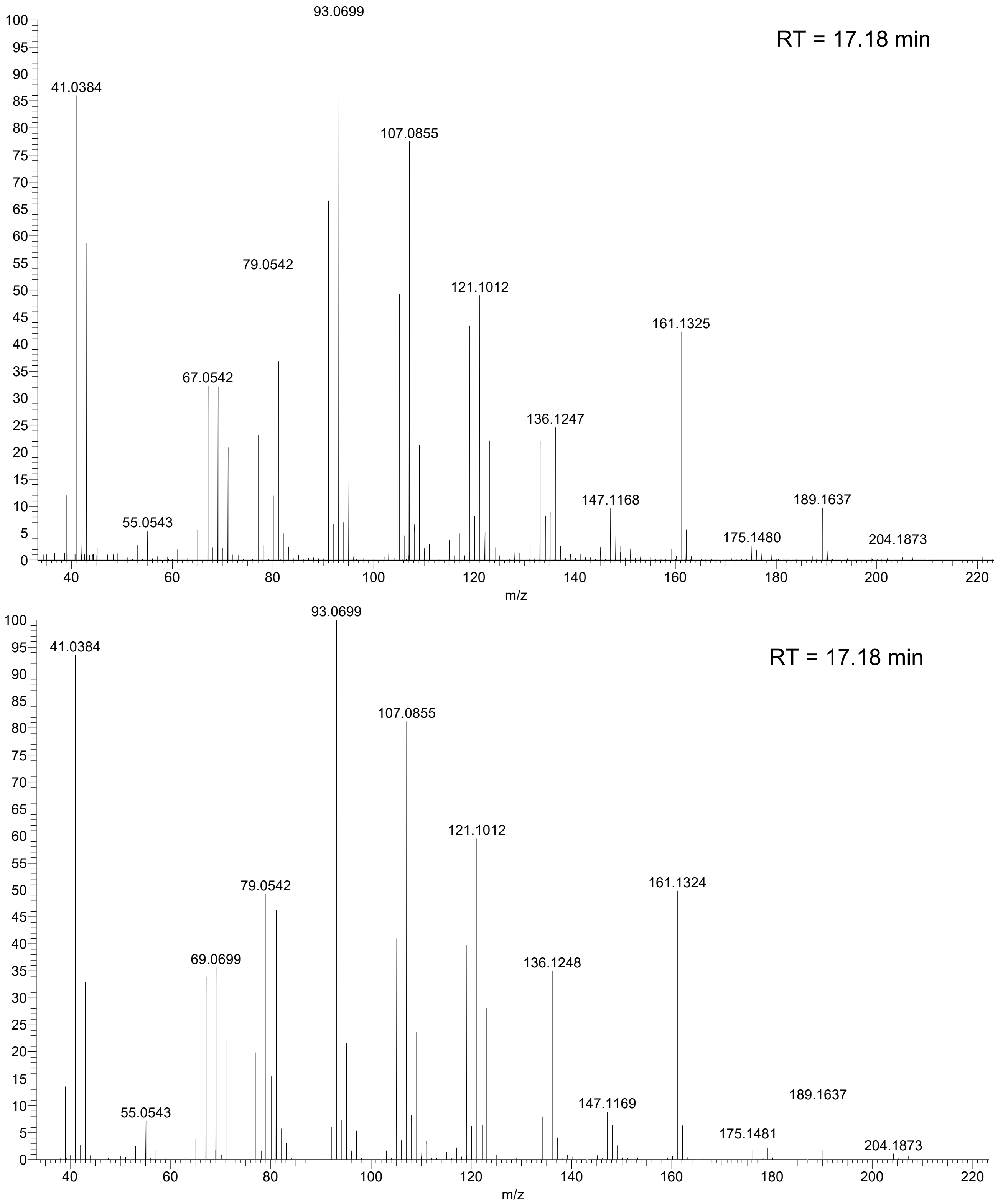

Supplementary Figure 96. The high resolution GC mass spectra of **16** and the nerolidol authentic standard (*bottom*).

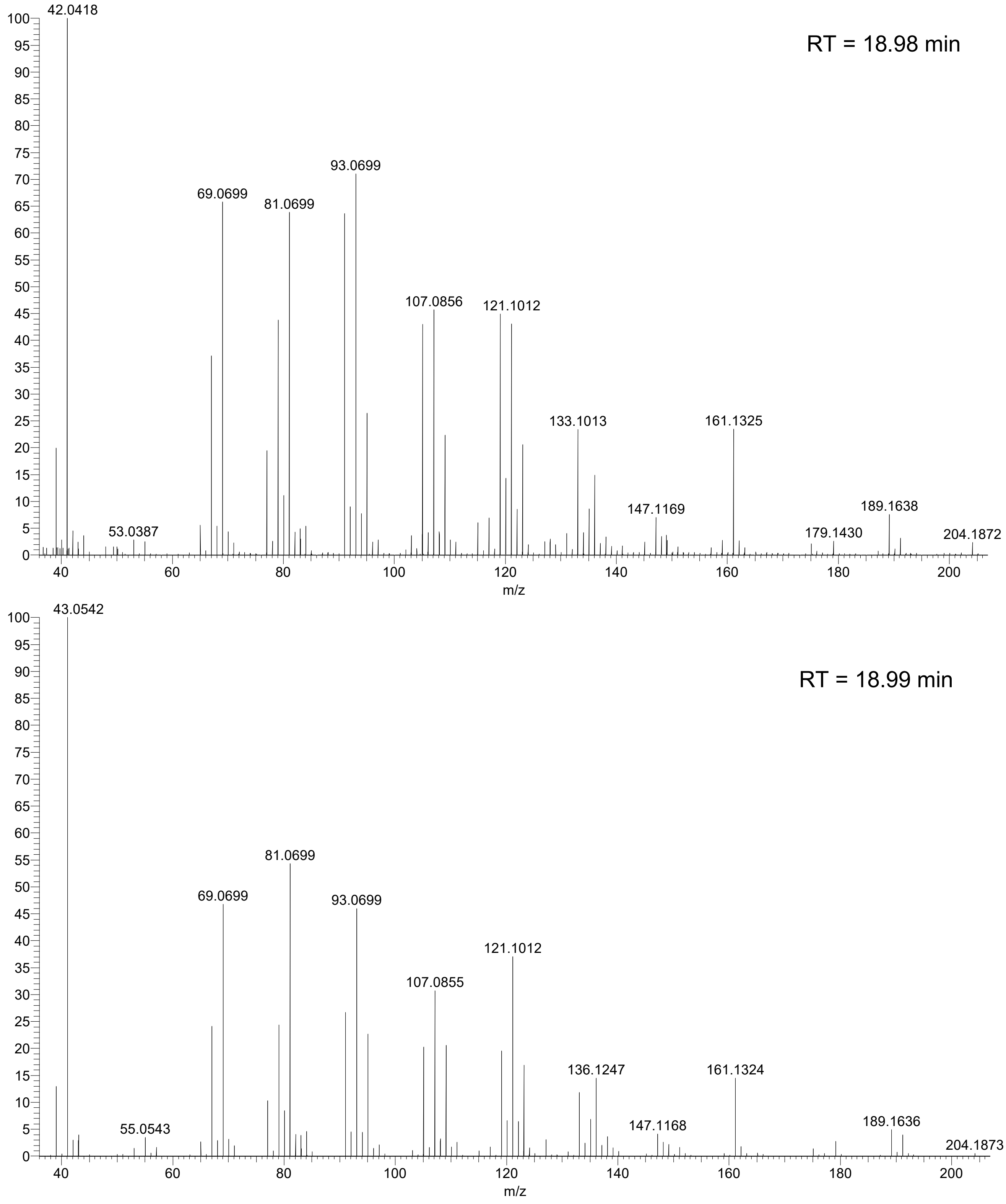

Supplementary Figure 97. The high resolution GC mass spectra of **17** and the farnesol authentic standard (*bottom*).

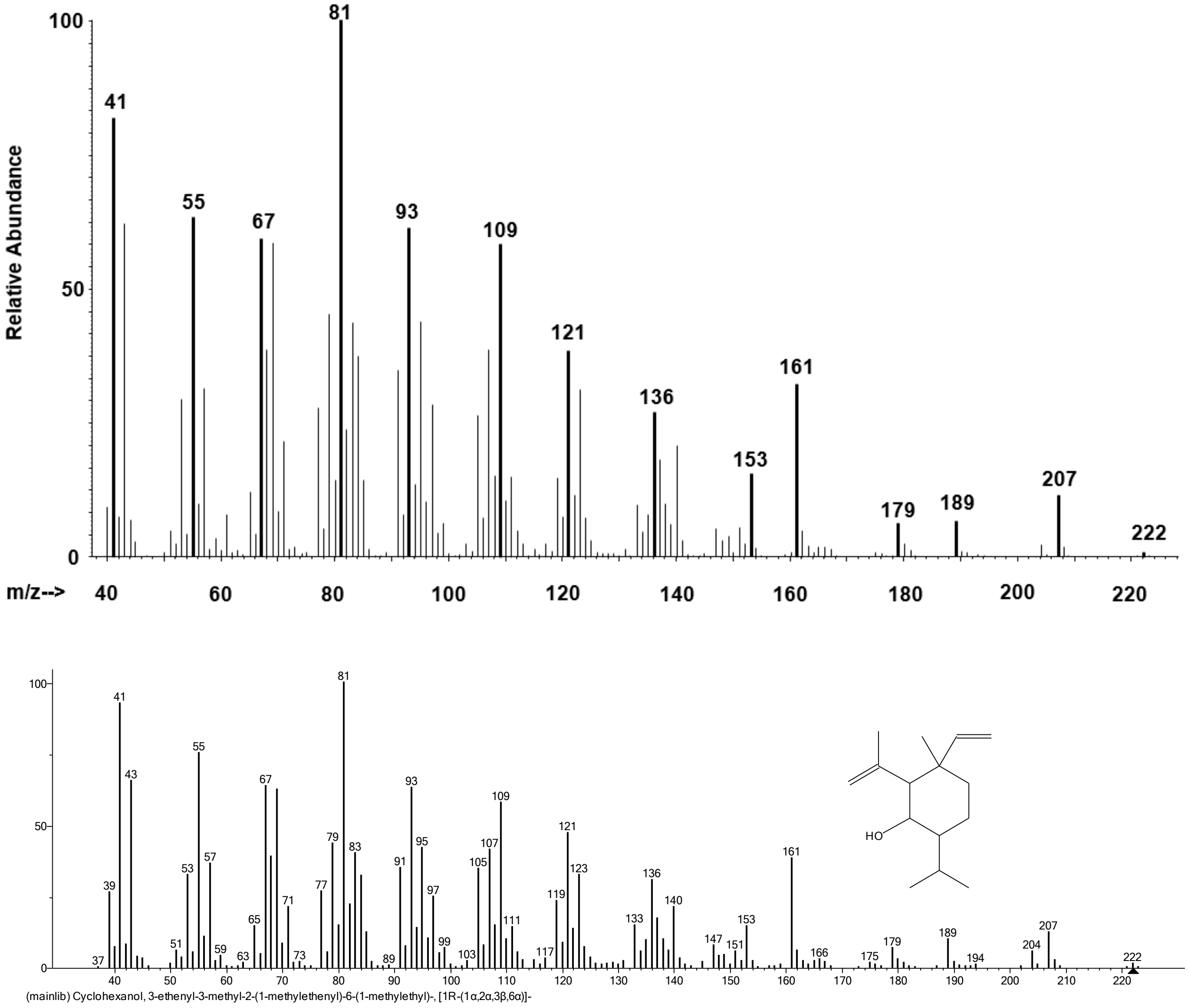

Supplementary Figure 98. The GC mass spectrum of **18*** (*top*) and the standard mass spectrum of shyobunol in the NIST library (*bottom*).

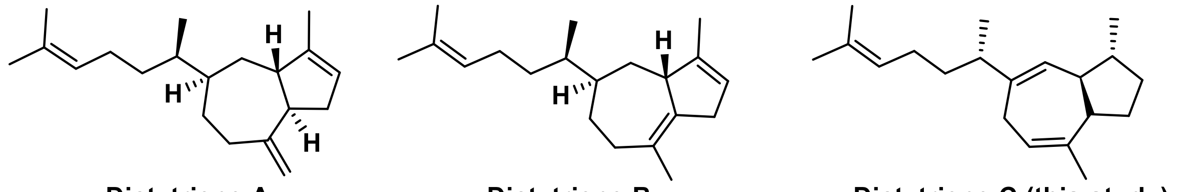

Dictytriene A **Dictytriene B** **Dictytriene C (this study)**

Supplementary Figure 99. The chemical structures of dictytrienes A, B and C.

Supplementary References

1. Kumar, S., Stecher, G., Li, M., Knyaz, C., Tamura, K. MEGA X: molecular evolutionary genetics analysis across computing platforms. *Mol. Biol. Evol.* **35**, 1547-1549 (2018).

2. Jumper, J., *et al.* Highly accurate protein structure prediction with AlphaFold. *Nature* **596**, 583-589 (2021).

3. Mitić, V. D., Ilić, M. D., Jovanović, O., Stankov-Jovanović, V. P., Marković, M. S., Stojanović, G. S. Essential oil composition of *Xanthium italicum* from Serbia. *Nat. Prod. Commun.* **14**, 1934578X19849968 (2019).

4. Li, Z., *et al.* First trans-eunicellane terpene synthase in bacteria. *Chem* **9**, 698-708 (2023).

5. Rabe, P., *et al.* Conformational analysis, thermal rearrangement, and EI-MS fragmentation mechanism of (1(10)*E*,4*E*,6*S*,7*R*)-germacradien-6-ol by ^{13}C-labeling experiments. *Angew. Chem. Int. Ed.* **54**, 13448-13451 (2015).

6. Rinkel, J., Lauterbach, L., Rabe, P., Dickschat, J. S. Two diterpene synthases for spiroalbatene and cembrene A from *Allokutzneria albata*. *Angew. Chem. Int. Ed.* **57**, 3238-3241 (2018).

7. Nobuyasu, E., Ryoichi, I., Shiro, U., Masamitsu, O., Takashi, T., Takeshi, M. New hydroazulenoid diterpenesI from the marine alga *Dictyota dichotoma*. *Chem. Lett.* **11**, 1837-1840 (1982).

8. Chen, N., Zhou, J., Li, J., Xu, J., Wu, R. Concerted cyclization of lanosterol C-ring and D-ring under human qxidosqualene cyclase catalysis: an ab initio QM/MM MD study. *J. Chem. Theory Comput.* **10**, 1109-1120 (2014).

9. Chen, Y.-G., *et al.* A cryptic plant terpene cyclase producing unconventional 18- and 14-membered macrocyclic C25 and C20 terpenoids with immunosuppressive activity. *Angew. Chem. Int. Ed.* **60**, 25468-25476 (2021).

10. Baer, P., *et al.* Induced-fit mechanism in class I terpene cyclases. *Angew. Chem. Int. Ed.* **53**, 7652-7656 (2014).

11. Li, Z., *et al.* Fragrant venezuelaenes A and B with a 5–5–6–7 tetracyclic skeleton: discovery, biosynthesis, and mechanisms of central catalysts. *ACS Catal.* **10**, 5846-5851 (2020).

12. Boys, S. F., Bernardi, F. The calculation of small molecular interactions by the differences of separate total energies. Some procedures with reduced errors. *Mol. Phys.* **19**, 553-566 (1970).

13. Hanahan, D. Studies on transformation of *Escherichia coli* with plasmids. *J. Mol. Biol.* **166**, 557-580 (1983).

14. Jeong, H., *et al.* Genome sequences of *Escherichia coli* B strains REL606 and BL21 (DE3). *J. Mol. Biol.* **394**, 644-652 (2009).

15. Yang, J., Nie, Q. Engineering *Escherichia coli* to convert acetic acid to beta-caryophyllene. *Microbial cell factories* **15**, 74-82 (2016).

16. Ioannou, E., Quesada, A., Rahman, M. M., Gibbons, S., Vagias, C., Roussis, V. Dolabellanes with antibacterial activity from the brown alga *Dilophus spiralis*. *J. Nat. Prod.* **74**, 213-222 (2011).

17. Köksal, M., Jin, Y., Coates, R. M., Croteau, R., Christianson, D. W. Taxadiene synthase structure and evolution of modular architecture in terpene biosynthesis. *Nature* **469**, 116-120 (2011).

18. Chen, M., Chou, W. K. W., Toyomasu, T., Cane, D. E., Christianson, D. W. Structure and function of fusicoccadiene synthase, a hexameric bifunctional diterpene synthase. *ACS Chem. Biol.* **11**, 889-899 (2016).

19. Driller, R., *et al.* Towards a comprehensive understanding of the structural dynamics of a bacterial diterpene synthase during catalysis. *Nat. Commun.* **9**, 3971 (2018).

20. Yuan, Y., *et al.* Efficient exploration of terpenoid biosynthetic gene clusters in filamentous fungi. *Nat. Catal.* **5**, 277-287 (2022).

21. Ronnebaum, T. A., Gardner, S. M., Christianson, D. W. An aromatic cluster in the active site of *epi-*

isozizaene synthase is an electrostatic toggle for divergent terpene cyclization pathways. *Biochemistry* **59**, 4744-4754 (2020).

22. Starks, C. M., Back, K., Chappell, J., Noel, J. P. Structural basis for cyclic terpene biosynthesis by tobacco 5-*epi*-aristolochene synthase. *Science* **277**, 1815-1820 (1997).

23. Blank, P. N., Shinsky, S. A., Christianson, D. W. Structure of sesquisabinene synthase 1, a terpenoid cyclase that generates a strained [3.1.0] bridged-bicyclic product. *ACS Chem. Biol.* **14**, 1011-1019 (2019).

24. Vedula, L. S., Zhao, Y., Coates, R. M., Koyama, T., Cane, D. E., Christianson, D. W. Exploring biosynthetic diversity with trichodiene synthase. *Arch. Biochem. Biophys.* **466**, 260-266 (2007).

25. Hyatt, D. C.*, et al.* Structure of limonene synthase, a simple model for terpenoid cyclase catalysis. *Proc. Natl. Acad. Sci. USA* **104**, 5360-5365 (2007).

26. Kumar, R. P.*, et al.* Structural characterization of early michaelis complexes in the reaction catalyzed by (+)-limonene synthase from *Citrus sinensis* using fluorinated substrate analogues. *Biochemistry* **56**, 1716-1725 (2017).

27. Karuppiah, V.*, et al.* Structural basis of catalysis in the bacterial monoterpene synthases linalool synthase and 1,8-cineole synthase. *ACS Catal.* **7**, 6268-6282 (2017).

28. Geu-Flores, F.*, et al.* An alternative route to cyclic terpenes by reductive cyclization in iridoid biosynthesis. *Nature* **492**, 138-142 (2012).

29. Tomita, T.*, et al.* Structural insights into the CotB2-catalyzed cyclization of geranylgeranyl diphosphate to the diterpene cyclooctat-9-en-7-ol. *ACS Chem. Biol.* **12**, 1621-1628 (2017).

30. Schriever, K.*, et al.* Engineering of ancestors as a tool to elucidate structure, mechanism, and specificity of extant terpene cyclase. *J. Am. Chem. Soc.* **143**, 3794-3807 (2021).
