# Supplementary material for: Molecular insights into the catalytic promiscuity of a bacterial diterpene synthase
Source: Nat Commun. 2023 Jul 6;14:4001. doi: 10.1038/s41467-023-39706-9 (PMC10325987; doi:10.1038/s41467-023-39706-9)
Supplement: Supplementary file 2 — Reporting Summary [file 41467_2023_39706_MOESM2_ESM.pdf]

nature portfolio

Corresponding author(s): Shengying Li

Last updated by author(s): Jun 4, 2023

Reporting Summary

Nature Portfolio wishes to improve the reproducibility of the work that we publish. This form provides structure for consistency and transparency in reporting. For further information on Nature Portfolio policies, see our Editorial Policies and the Editorial Policy Checklist.

Statistics

For all statistical analyses, confirm that the following items are present in the figure legend, table legend, main text, or Methods section.

n/a	Confirmed	
☐	☒	The exact sample size (*n*) for each experimental group/condition, given as a discrete number and unit of measurement
☐	☒	A statement on whether measurements were taken from distinct samples or whether the same sample was measured repeatedly
☒	☐	The statistical test(s) used AND whether they are one- or two-sided<br>*Only common tests should be described solely by name; describe more complex techniques in the Methods section.*
☒	☐	A description of all covariates tested
☒	☐	A description of any assumptions or corrections, such as tests of normality and adjustment for multiple comparisons
☐	☒	A full description of the statistical parameters including central tendency (e.g. means) or other basic estimates (e.g. regression coefficient) AND variation (e.g. standard deviation) or associated estimates of uncertainty (e.g. confidence intervals)
☒	☐	For null hypothesis testing, the test statistic (e.g. *F*, *t*, *r*) with confidence intervals, effect sizes, degrees of freedom and *P* value noted<br>*Give P values as exact values whenever suitable.*
☒	☐	For Bayesian analysis, information on the choice of priors and Markov chain Monte Carlo settings
☒	☐	For hierarchical and complex designs, identification of the appropriate level for tests and full reporting of outcomes
☒	☐	Estimates of effect sizes (e.g. Cohen's *d*, Pearson's *r*), indicating how they were calculated

Our web collection on statistics for biologists contains articles on many of the points above.

Software and code

Policy information about availability of computer code

Data collection	Agilent Openlab Control Panel 2.4.0.628, Thermo Chromeleon Console 7.0, Bruker TopSpin 4.1.4, Thermo Scientific Xcalibur 4.2 SP1, SpectraManager 2.15.01, Amber20,Gaussian 16 Revision A.03, AutoDock vina 1.2.2, PROTEUM 3, Rigaku CrysAlis Pro 40
Data analysis	Thermo Scientific Xcalibur 4.2 SP1, PyMOL program 2.5.1, AutoDock vina 1.2.2, SpectraManager 2.15.01, MEGA-X, Cytoscape 3.9.1, Refmac5,PHENIX 1.17.1, Coot 0.8.9, CCP4 7.0.071, Bruker TopSpin 4.1.4, Agilent Openlab Control Panel 2.4.0.628, Thermo Chromeleon Console 7.0

For manuscripts utilizing custom algorithms or software that are central to the research but not yet described in published literature, software must be made available to editors and reviewers. We strongly encourage code deposition in a community repository (e.g. GitHub). See the Nature Portfolio guidelines for submitting code & software for further information.

Data

Policy information about availability of data

All manuscripts must include a data availability statement. This statement should provide the following information, where applicable:

- Accession codes, unique identifiers, or web links for publicly available datasets
- A description of any restrictions on data availability
- For clinical datasets or third party data, please ensure that the statement adheres to our policy

The structural data of apo form and PPi-(Mg2+)3-bound form of VenA generated in this study have been deposited in the Protein Data Bank (PDB) database under accession codes 7Y9H and 7Y9G, respectively. The crystal structure of SvS-A2 used in this study is available in the PDB database under accession code 6TBD. The

structural data of 9 and 11 generated in this study have been deposited in the Cambridge Crystallographic Data Centre (CCDC) under accession codes 2163519 and 2184782, respectively. All other relevant data supporting the findings of this study are available in this manuscript and Supplementary Information.

Research involving human participants, their data, or biological material

Policy information about studies with human participants or human data. See also policy information about sex, gender (identity/presentation), and sexual orientation and race, ethnicity and racism.

Reporting on sex and gender	N/A
Reporting on race, ethnicity, or other socially relevant groupings	N/A
Population characteristics	N/A
Recruitment	N/A
Ethics oversight	N/A

Note that full information on the approval of the study protocol must also be provided in the manuscript.

Field-specific reporting

Please select the one below that is the best fit for your research. If you are not sure, read the appropriate sections before making your selection.

☒ Life sciences ☐ Behavioural & social sciences ☐ Ecological, evolutionary & environmental sciences

For a reference copy of the document with all sections, see nature.com/documents/nr-reporting-summary-flat.pdf

Life sciences study design

All studies must disclose on these points even when the disclosure is negative.

Sample size	Following common practice, the enzyme activities of wild type and each mutants were independently determined in triplicate.
Data exclusions	No data were artificially excluded from the analyses.
Replication	Three independent experiments were conducted, and all attempts in replication were successful. The representative results are displayed in Figure 3a. It should be noted that each crystal structure herein was determined from a single crystal.
Randomization	To determine the relative catalytic efficiency, the reactions of wild-type and mutant enzymes were allocated into experimental groups, and the corresponding boiling-inactivated enzymes were set as control experiments. Free-R flag for crystallographic data was assigned randomly to unique reflections using CCP4 program or Phenix.
Blinding	All samples were labeled and assayed, and no blinding was applied to the present study.

Reporting for specific materials, systems and methods

We require information from authors about some types of materials, experimental systems and methods used in many studies. Here, indicate whether each material, system or method listed is relevant to your study. If you are not sure if a list item applies to your research, read the appropriate section before selecting a response.

Materials & experimental systems

n/a	Involved in the study	
☒	☐	Antibodies
☒	☐	Eukaryotic cell lines
☒	☐	Palaeontology and archaeology
☒	☐	Animals and other organisms
☒	☐	Clinical data
☒	☐	Dual use research of concern
☒	☐	Plants

Methods

n/a	Involved in the study	
☒	☐	ChIP-seq
☒	☐	Flow cytometry
☒	☐	MRI-based neuroimaging
